# Adsorption of some cationic dyes onto two models of graphene oxide

**DOI:** 10.1007/s00894-023-05761-8

**Published:** 2023-11-18

**Authors:** Emma Mounra, Alhadji Malloum, Jean Jules Fifen, Jeanet Conradie

**Affiliations:** 1 Department of Physics, Faculty of Science, University of Ngaoundere, PO BOX 454, Ngaoundere, Cameroon; 2 Department of Chemistry, University of the Free State, PO BOX 339, Bloemfontein, 9300 South Africa; 3 Department of Physics, Faculty of Science, University of Maroua, PO BOX 46, Maroua, Cameroon; 4 Department of Chemistry, UiT - The Arctic University of Norway, N-9037 Tromsø, Norway

**Keywords:** Cationic dyes, Graphene oxide, Adsorption, Computational method

## Abstract

****Context**:**

The search for highly efficient adsorbent materials remains a significant requirement in the field of adsorption for wastewater treatment. Computational study can highly contribute to the identification of efficient material. In this work, we propose a computational approach to study the adsorption of four cationic basic dyes, basic blue 26 (BB26), basic green 1 (BG1), basic yellow 2 (BY2), and basic red 1 (BR1), onto two models of graphene oxide as adsorbents. The main objectives of this study are the assessment of the adsorption capacity of the graphene oxide towards basic dyes and the evaluation of the environmental and temperature effects on the adsorption capacity. Quantum theory of atoms in molecules (QTAIM) analysis has been used to understand the interactions between the dyes and graphene oxides. In addition, adsorption free energies of the dyes onto graphene oxides are calculated in gas and solvent phases for temperatures varying from 200 to 400 K. As a result, the adsorption free energy varies linearly depending on the temperature, highlighting the importance of temperature effects in the adsorption processes. Furthermore, the results indicate that the environment (through the solvation) considerably affects the calculated adsorption free energies. Overall, the results show that the two models of graphene oxide used in this work are efficient for removing dyes from wastewater.

****Methods**:**

We have optimized the complexes formed by the interaction of dyes with graphene oxides at the PW6B95-D3/def2-SVP level of theory. The SMD solvation model realizes the implicit solvation, and water is used as the solvent. Calculations are performed using the Gaussian 16 suite of program. QTAIM analysis is performed using the AIMAll program. Gibbs free energies as function of temperature are calculated using the TEMPO program.

**Supplementary Information:**

The online version contains supplementary material available at 10.1007/s00894-023-05761-8.

## Introduction

Organic compounds, dyes, antioxidants, and pharmaceutical and personal care products are the primary pollutants that cause directly and indirectly damage and threaten human health. Emerging dyes are used in many sectors, such as textile sorting, printing, dyeing, and plastic production of plastics [1]. However, they can be toxic and persistent in the environment, affecting ecosystems and human health. It is possible to develop efficient and robust adsorbents for dye adsorption. These dyes can contain functional groups, natural or derived from chemical reactions or synthesis. The theoretical study of the adsorption of emerging pollutants helps better understand the interactions between the dye molecules and the material’s surface. These results can be used to design more efficient adsorption materials to predict the efficiency of the adsorbent in de-pollution and improve the quality of the water discharged into the environment.

Previous work highlights the importance of the adsorption of basic dyes and provides avenues for developing new water treatment methods to eliminate emerging pollutants. Regti et al. [2] studied the adsorption of two cationic dyes (basic yellow and methylene blue) using both DFT (density functional theory) and experimental methods. The DFT study was realized using the B3LYP exchange and correlation functional, associated with the basis set 6-31 G(d,p). The authors reported the electrophilicity and nucleophilicity of dyes. Dastgerdi et al. [3] have studied the elimination of indigo carmine dye by a nanotube carbon functionalized using DFT. Adsorption energies show that the functionalization improves the adsorption capacity of carbon nanotubes. Huang et al. [4] performed a molecular dynamic (MD) simulation for the adsorption of methylene blue by the bituminous coal. The authors monitored the interaction and calculated the interaction energy. The free energy of adsorption was negative, indicating that the adsorption formation of methylene blue was a spontaneous process. In a recent study, Shen et al. used graphene oxide to adsorb azo dyes such as methyl orange, methyl blue, and methyl red. They found that graphene oxide effectively removed these dyes from water. Kumar et al. [5] studied the adsorption of basic 26 on nanoparticles of zinc oxide functionalized with graphene. The results showed efficient adsorption of this dye, with a strong affinity for the nanoparticles functionalized zinc oxide, offering thus a potential method for its removal from the water. Luo et al. [6] performed DFT calculations for the study of the adsorption of acid blue 25 on the cucurbit[8]uril. The authors used B3LYP/6-31+G(d) as the theoretical level of computation and the PCM solvation model to compare the UV-Vis spectrum with the experimental results. In addition, De Souza et al. [7] have studied the adsorption of BB26, BG1, BY2, and BR1 onto activated carbon using B3LYP/6-31 G(d) level of theory. According to the experimental and theoretical results, the dyes BG1 and BB26 are more reactive than BR1 and BY2. It should be noted that several studies of this type have been carried out on different types of dyes (for example, Congo red and black dye heterochrome T) and using different adsorbents (magnetic MOF, graphene, nanotubes, or graphene and functionalized nanotubes) [8, 9].

Using molecular dynamic simulations, the adsorption of dyes onto models of graphene oxide has been reported by several authors in the literature [10–18]. Adsorption of methylene blue onto graphene oxide has been studied using molecular dynamics by Hou and coworkers [16]. Recently, Gao et al. [18] estimated the adsorption energy of methylene blue and methyl orange onto reduced graphene oxide to be $-$244.3 k/J mol$^{-1}$ and $-$124.1 mol$^{-1}$, respectively. Earlier, Molla et al. [14] performed a molecular dynamics simulation of the adsorption of methylene blue and methyl orange onto graphene oxide similar to Gao et al. [18]. They concluded that methylene blue is more tightly adsorbed on graphene oxide than methyl orange. Besides, the adsorption of methylene blue onto graphene oxide is reported by Tanis et al. [17].

The exploration of the literature shows that few of the theoretical works have been reported. The search for efficient adsorbent materials remains a significant requirement in the field of adsorption for wastewater treatment. Most of the calculations in the literature are reported using electronic energy. It is necessary to use the Gibbs free energy to evaluate the adsorption process. The Gibbs free energy of adsorption considers the entropic and temperature effects on the adsorption. Despite some studies on implicit solvation, most research is carried out without considering the solvent effects on adsorption energy. In the present study, we studied the adsorption of four cationic basic dyes, BB26, BG1, BY2, and BR1, onto two models of graphene oxide as adsorbents. Calculations are performed in the gas and solvent phase using the PW6B95-D3 [19] functional by adopting the def2-SVP [20] basis set. In order to obtain an accurate description of adsorption, we propose a methodological approach to calculate the free energy of adsorption for temperatures ranging from 200 to 400 K.

## Methodology

This section starts with the presentation of the two models of graphene oxide used in this work (see Sect. 2.1, Graphene oxide models). Then, the equations used to calculate some DFT-based descriptors of the studied dyes (see Sect. 2.2, DFT-based descriptor calculations), followed by the adsorption free energy calculations (see Sect. 2.3, Adsorption free energy). Finally, the computational details used in this work are presented in Sect. 2.4 (Computational details).

### Graphene oxide models

**Fig. 1:**
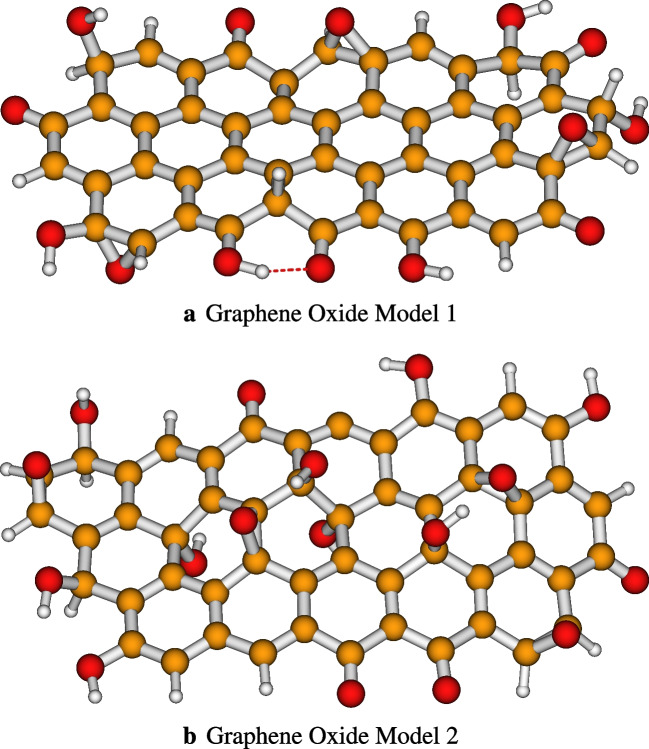
Optimized structures of the two graphene oxide models used in this work for the adsorption of four dyes. Optimization has been performed at the PW6B95-D3/def2-SVP level of theory

In the adsorption of dyes onto graphene oxide, some types of graphene oxide are effective in removing different dyes (basics, acids, reagents, and others) or even dyes of the same class, but which differ in their chemical structures. Graphene oxide (GO) has a structure similar to that of graphene. These two materials have a hexagonal carbon lattice, but the GO sheet is usually distorted where it is bound to oxygen groups [21]. The researchers have proposed many theoretical models for GO, but the precise chemical structure is still controversial. Graphene oxide or GO is strongly oxygenated by hydroxyl and epoxide groups on $sp^3$ hybridized carbons in the basal plane, as well as by carbonyl and carboxyl groups located at the level of the edges of the leaflets on the hybridized carbons $sp^2$ [22]. The functional groups on the graphene oxide surface make it very chemically reactive. It can react with different molecules and be functionalized for specific applications. The GO is much easier to use with the method of Hummers [23] and offers the potential for a production that is effective and large-scale. Based on the information presented above, two models on graphene oxide have been built (see Fig. 1). Both models used in our work stand out regarding their chemical structure based on the arrangement of oxygen atoms.

### DFT-based descriptor calculations

Chemical quantum molecular descriptors of the studied dyes have been calculated in this work to understand their reactivity. These descriptors are calculated using the frontier molecular orbitals HOMO (highest occupied molecular orbital) and LUMO (lowest unoccupied molecular orbital). The energy gap values HOMO-LUMO ($\varepsilon _{gap}$) are calculated using Eq. 1. Values of global indices such as chemical potential ($\mu$), chemical hardness ($\eta$), and electrophile index ($\omega$) were estimated in terms of frontier molecular orbital energies HOMO and LUMO, $\varepsilon _H$ and $\varepsilon _L$, using Eqs. 2, 3, and 4, respectively. Similarly, the electron affinity (*EA*) and the ionization potential (*IP*) are calculated using Eqs. 5 and 6, respectively.

1 $$\begin{aligned} \varepsilon _{gap}= & {} \varepsilon _L-\varepsilon _H, \end{aligned}$$

2 $$\begin{aligned} \mu= & {} \frac{(\varepsilon _H+\varepsilon _L)}{2}, \end{aligned}$$

3 $$\begin{aligned} \eta= & {} \frac{(\varepsilon _L-\varepsilon _H)}{2}, \end{aligned}$$

4 $$\begin{aligned} \omega= & {} \frac{\mu ^2}{2\eta }, \end{aligned}$$

5 $$\begin{aligned} EA= & {} -\varepsilon _L ,\end{aligned}$$

6 $$\begin{aligned} IP= & {} -\varepsilon _H. \end{aligned}$$

### Adsorption free energy

The free energies of the complexes (dye + GO) at different temperatures are calculated using Fifen and coworkers’ TEMPO program [24, 25]. The free energies of adsorption of dyes onto graphene oxide are calculated from 200 to 400 K for the increment of 20 K. The free energy of adsorption can be calculated by using Eq. 7.

7 $$\begin{aligned} \Delta G_{Ads}(T) =\Delta G_{GOP}(T) - \Delta G_{GO}(T) - \Delta G_P(T), \end{aligned}$$

where $\Delta G_{GOP}$(T) is the free energy of the complex graphene oxide + pollutant (dye), $\Delta G_{GO}$ (T) is the free energy of the oxide of graphene, and $\Delta G_P$(T) is the free energy of the pollutant. The adsorption free energy of Eq. 7 is calculated in the gas phase, and therefore, all the free energies involved are also calculated in the gas phase. In the case of implicit solvation, the adsorption energy is calculated using the same equation as follows:

8 $$\begin{aligned} \Delta G_{Ads}^{solv}(T) = \Delta G_{GOP}^{solv}(T) - \Delta G_{GO}^{solv}(T) - \Delta G_{P}^{solv}(T). \end{aligned}$$

The difference between Eqs. 7 and 8 is that the free energies in the latter are calculated in the implicit solvent phase to consider the environmental effects. Notably, the adsorption electronic energies in gas and solvent phases have also been calculated using similar equations to those used for the adsorption free energies.

### Computational details

In this section, we comprehensively describe the computational approach used to study the adsorption of dyes (BB26, BG1, BY2, and BR1) onto two models of graphene oxide as adsorbents. The adsorption is studied in both the gas phase and implicit solvent phase. Molecular structures of the dyes were built using the Avogadro [26] software and then Gaussview software. The structures are fully optimized at the level of theory PW6B95-D3/def2-SVP [27]. The theory PW6B95-D3 has been used in the literature, and it has shown good performance in the calculation of binding energies [28–30]. Recently, a benchmark of 16 functionals has been performed to calculate the adsorption free energy of four pollutants onto coronene [31]. It has been found that the PW6B95-D3 is among the best functionals recommended to study adsorption processes [31]. All optimizations and calculations of frequency are realized using the sequence of Gaussian programs 16 [32]. Optimization is performed using the tight option for accuracy. The ultrafine grid was used for the integral calculations. Implicit solvation is performed using the implicit model named SMD [33] (solvation model based on density), where water is used as a solvent.

A QTAIM analysis is performed on the complexes to study the interactions between the dyes and the graphene oxides. The AIMAll program performs the QTAIM analysis [34].

## Results and discussions

For the sake of the completeness of the study, the investigation started with calculating some DFT-based descriptors of the dyes to understand their reactivity. These calculations exploit the frontier molecular orbital energies. After calculating the descriptors, the structures of the complexes formed by the dyes and the two models of graphene oxide are optimized at the PW6B95-D3/def2-SVP level of theory. To understand the interaction between the dyes and the GO models, QTAIM analysis of the eight complexes is performed. Using the optimized structures of the complexes, the adsorption electronic energies and the adsorption free energies at room temperature are calculated. Finally, the effects of the temperature on the adsorption free energies of the complexes are assessed and presented.

### DFT-based descriptors of the studied dyes

**Fig. 2:**
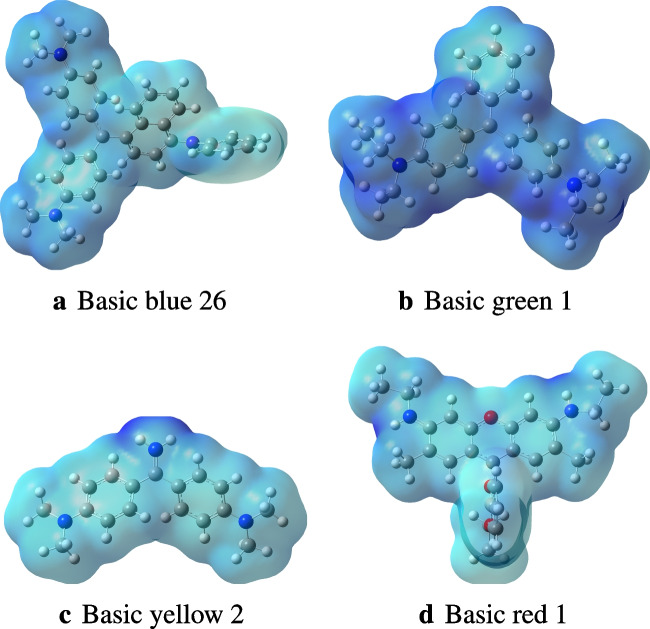
Electrostatic potential of the four dyes BB26, BG1, BY2, and BR1, determined in the gas phase, based on the electron density

Molecular electronic descriptors and their calculated values are shown in Table 1. Molecular orbitals play an important role in understanding the chemical reactivity at atomic level. The energy gap between the frontier orbitals is associated with properties such as molecular reactivity and kinetic stability [35]. To determine the most reactive sites to metallic attacks, namely electrophilic and nucleophilic attacks, a qualitative approach to the main charge transfers and atomic interactions has been carried out with the electrostatic potential model analysis (MESP). The MESP makes it possible to determine the most reactive sites towards interactions with other molecules/atoms. The MESPs of the four dyes are reported in Fig. 2. An abundance of electrons characterizes the low potential areas (red-colored surface), while high potential areas (blue-colored surface) are characterized by a relative absence of electrons (see Fig. 2).

**Table 1:** DFT-based descriptors of the four dyes computed at the PW6B95-D3/def2-SVP level of theory

	$\varepsilon _H$	$\varepsilon _L$	$\varepsilon _{gap}$	$\mu$	$\eta$	$\omega$	*EA*	*IP*
Gas phase								
BB26	$-$8.1	$-$5.4	2.7	$-$6.7	1.4	16.6	5.4	8.1
BG1	$-$8.5	$-$5.6	2.9	$-$7.0	1.5	16.9	5.6	8.5
BY2	$-$8.8	$-$5.2	3.6	$-$7.0	1.8	13.5	5.2	8.8
BR1	$-$8.5	$-$5.2	3.4	$-$6.9	1.7	13.9	5.2	8.5
Solvent								
BB26	$-$5.8	$-$2.9	2.9	$-$4.3	1.4	6.5	2.9	5.8
BG1	$-$5.8	$-$2.9	2.9	$-$4.3	1.4	6.5	2.9	5.8
BY2	$-$5.8	$-$2.1	3.7	$-$4.0	1.9	4.2	2.1	5.8
BR1	$-$5.9	$-$2.6	3.3	$-$4.2	1.7	5.3	2.6	5.9

The energy unit in the table is eV

The values of the electrophilicity index ($\omega$) for BB26 ($\omega$ = 16.6 eV) and BG1 ($\omega$ = 16.9 eV) suggest that these molecules are highly electrophile. In comparison, BY2 ($\omega$ = 13.5 eV) and BR1 ($\omega$ = 13.9 eV) are less electrophile than BB26 and BG1. Similar observations are found in the solvent solvent phase (Table 1). The electrophilicity index ($\omega$) can be interpreted as a measure of the lowering of energy due to the maximum flow of electrons between the donor and acceptor [36]. A strong and more reactive electrophile is characterized by a high value of the chemical potential ($\mu$) and of the electrophilicity index ($\omega$) [37]. The stability of a molecule and its reactivity can be related to the chemical hardness ($\eta$). The molecules BB26 ($\eta$ = 1.4) and BG1 ($\eta$ = 1.5) have low hardness values as compared to those of BY2 ($\eta$ = 1.8) and BR1 ($\eta$ = 1.7). The molecules that have low hardness values are more reactive (see Table 1). Overall, analysis of the quantum chemical descriptors of the four dyes indicates that BB26 and BG1 are more reactive than BY2 and BR1 in the gas phase. A similar conclusion is found for the calculations performed in the solvent phase. However, the values of the descriptors calculated in the solvent are smaller than those calculated in the gas phase. This is ascribed to the effects of the implicit solvation. Therefore, it is essential to consider the solvent’s effects in calculations of quantum chemical parameters. In comparing the results with those obtained in the literature, De Souza and his collaborators [38] also found that the molecules BG1 and BB26 are more reactive than BR1 and BY2 using the B3LYP/6-31 G(d) level of theory. Although we derived the same conclusion as de Souza and coworkers [38], the calculated values of the descriptors present a considerable difference due to the computational level of theory. In addition to the descriptors reported above, the frontier molecular orbitals (HOMO and LUMO) of the studied dyes are reported in Fig. S2 of the supporting information.

### Structures of the complexes GO+Dye and AIM analysis

**Fig. 3:**
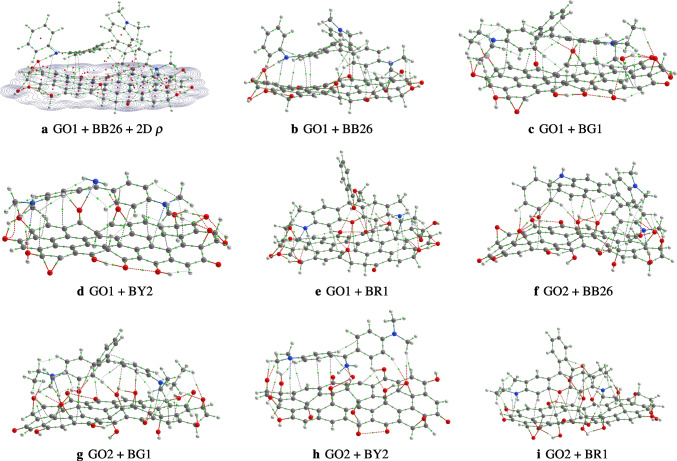
Optimized geometries of the dyes in interaction with the two models of graphene oxide. Bond paths and bond critical points of the systems are also represented based on the electron density calculated at the PW6B95-D3/def2-SVP level of theory. The geometries are reported in the gas phase

To calculate the adsorption energy, one needs the structures of the complexes formed by the combination of a dye and a GO model, in addition to the individual structures of the GO models and the dyes. Thus, we formed eight complexes by combining the GO models and the four dyes. After optimizations of the structures at the PW6B95-D3/def2-SVP level of theory, the located stable configurations are reported in Fig. 3. The reader is informed that due to the expense of the calculations, we have not been able to perform a conformation search to locate global and local minima structures on their respective potential energy surfaces. Thus, the structures reported in Fig. 3 are not necessarily the most stable configurations. Furthermore, the objective of this work is to quickly assess if the proposed graphene oxide models would be able to adsorb the four dyes. Therefore, even if the structures reported in Fig. 3 are not the global minimum energy structures, it is possible to address the objective of this work. Nevertheless, it is important to state that conformational search is necessary for the seek of accuracy of the calculated adsorption energies. Therefore, one can adopt a cheap exploration methodology to precisely locate the most stable structure in future works. A similar affordable exploration has been proposed recently by García-Hernández and coworkers [39–42].

To understand the interactions between the graphene oxide and the dyes, we performed a QTAIM analysis of the eight complexes. The structures reported in Fig. 3 are derived from the QTAIM analysis. We have reported the bond critical points (BCPs) and the bond paths (BPs) determined based on the electron density calculated at the PW6B95-D3/def2-SVP level of theory. For the complex GO1 + BB26, a 2D contour map of the electron density in the plan of the graphene oxide is reported in Fig. 3. Further QTAIM analysis data of the complexes is reported in the supporting information. The data includes the electron density $\rho$ at bond critical points, the Laplacian of the electron density, $\nabla ^2\rho$, the ellipticity, the kinetic energy, and the difference between the bond path length and the geometrical bond length. From the provided data, it is possible to identify non-covalent interactions, which mainly comprise the interactions between the dyes and the graphene oxides. A positive value of $\nabla ^2\rho$ at bond critical points represents a non-covalent bonding, while a negative value is representative of a covalent bonding [43, 44]. In addition, the data reported in the supporting information can be used to determine the strength of the non-covalent bonding in the systems. It has been found previously that the value of the electron density at a bond critical point is proportional to the strength of the corresponding bonding [45–52]. Thus, the higher the value of $\rho$ at BCP, the higher the strength of the corresponding bonding.

Analysis of Fig. 3 and the data reported in the supporting information shows that the non-covalent interactions between the dyes and the GO1 comprise CH$\cdots$O and NH$\cdots$O hydrogen bondings, CH$\cdots \pi$, $\pi \cdots \pi$, O$\cdots$O, and H$\cdots$H bonding interactions. In addition to the bondings mentioned above, the interaction between the dyes and GO2 has an OH$\cdots \pi$ bonding interaction. Based on the values of $\rho$ at BCP, it can be stated that the most strong non-covalent bondings in the complexes are the CH$\cdots$O and OH$\cdots$O hydrogen bondings. Besides, the smallest value of $\rho$ at BCP in the complexes is attributed to a $\pi \cdots \pi$ stacking interaction.

**Table 2:** Electronic and free energies (at room temperature) of dyes’ adsorption onto the two graphene oxide models

	$\Delta E_{Ads}^{mod1}$	$\Delta G_{Ads}^{mod1}$	$\Delta E_{Ads}^{mod2}$	$\Delta G_{Ads}^{mod2}$
Gas phase				
BB26	$-$217.6	$-$129.1	$-$183.5	$-$99.1
BG1	$-$189.8	$-$102.1	$-$158.7	$-$78.7
BY2	$-$201.3	$-$115.5	$-$124.7	$-$54.7
BR1	$-$230.5	$-$130.1	$-$169.2	$-$72.9
Solvent phase				
BB26	98.3	186.6	$-$166.6	$-$83.5
BG1	$-$163.3	$-$74.4	$-$126.6	$-$49.2
BY2	190.4	274.4	$-$92.4	$-$14.6
BR1	$-$169.6	$-$76.6	$-$175.1	$-$76.7

The electronic and free energies are reported in kJ mol$^{-1}$

### Adsorption energies

Using the structures reported in Fig. 3, the adsorption electronic energy and the adsorption Gibbs free energy are calculated using Eq. 7. In addition to the structures of Fig. 3, optimizing the dye structures (BB26, BG1, BY2, and BR1) and the graphene oxide structures is necessary. The numerically estimated values of the adsorption electronic energies and the adsorption free energies (at room temperature) are reported in Table 2. The electronic adsorption energy measures the interaction between the pollutants’ electron orbitals and the adsorbents. At the same time, the free energy of adsorption also considers the entropic effect of adsorption [53]. In both gas and solvent phases, the adsorption electronic and free energies obtained with GO1 are more negative than those obtained with GO2 (except for BB26 and BY2 in the solvent phase). The study has also shown that the adsorption of dyes onto graphene oxide is strongly dependent on the graphene oxide surface geometry. Elsewhere, the use of electronic energy is not the energy appropriate to accurately assess the adsorption energy due to the fact that the adsorption process takes place at temperatures other than 0 K. The electronic energy overestimates adsorbents’ adsorption capacity (see Table 2). The adsorption power must be evaluated using the free energy [54].

#### In the gas phase

For the first model of graphene oxide, the free energy values at room temperature are BB26 ($-$129.1 kJ mol$^{-1}$), BG1 ($-$102.1 kJ mol$^{-1}$), BY2 ($-$115.5 kJ mol$^{-1}$), and BR1 ($-$130.1 kJ mol$^{-1}$). It can be seen that BB26 and BR1 have the highest adsorption free energy values, suggesting a strong interaction between the dye and graphene oxide and their stabilities when adsorbed on the adsorbent. BY2 also has a relatively high adsorption free energy value. On the other hand, BG1 has the lowest adsorption free energy, indicating the lowest interaction with the adsorbent (see Table 2). For the second model of graphene oxide, the adsorption free energies are estimated as follows: BB26 ($-$99.1 kJ mol$^{-1}$), BG1 ($-$78.7 kJ mol$^{-1}$), BY2 ($-$54.7 kJ mol$^{-1}$) and BR1 ($-$72.9 kJ mol$^{-1}$). It can be observed that BB26, BG1, and BR1 have the highest adsorption free energy values, suggesting a strong interaction between the dye and the graphene oxide. BY2 has the lowest adsorption free energy value, indicating weaker interaction with the adsorbent, which may be less stable when adsorbed on graphene oxide (see Table 2). Other factors, such as the size and shape of the dyes, can also influence the stability of dyes when they are adsorbed onto the surface of an adsorbent. Graphene oxide model 1 is more efficient than model 2 for dye adsorption.

#### In the solvent phase

Adsorption electronic energies and adsorption free energies are compared to evaluate the effect of solvent and temperature on the adsorption of dyes onto graphene oxide. The results show that the adsorption free energy is more sensitive to solvent and temperature than energy adsorption electronic energy. The free energies of adsorption in the implicit solvent, $\Delta G_{Ads}^{solv}(T)$, are less negative than the free energies of adsorption reported in the gas phase, $\Delta G_{Ads}(T)$. It can be seen that the energy gap between gas and solvent phase energies of BG1 and BR1 shows that the adsorption of these dyes releases less energy into the solvent than in the gas. On the other hand, the adsorption electronic and free energies of BB26 and BY2 are found to be positive in the solvent phase (see Table 2). These positive values indicate that the adsorption does not happen spontaneously. Therefore, external energy is needed to initiate the adsorption. This result is not consistent with other findings in this work. Therefore, it is most likely that the structures of the complexes GO1+BB26 and GO1+BY2 are lying higher in energy above the fundamental global minimum energy structures on their PESs. Thus, a full conformational search would generate more stable configurations, leading to negative values of the adsorption electronic energies and free energies. Some authors have suggested cheap methodological approaches based on semiempirical methods to explore the PESs of the studied complexes [40–42]. These approaches can be used in future works to locate the global minimum energy structure that would lead to negative value of the adsorption free energy. The nature of the solvent plays an important role in the adsorption process (see Table 2).

**Fig. 4:**
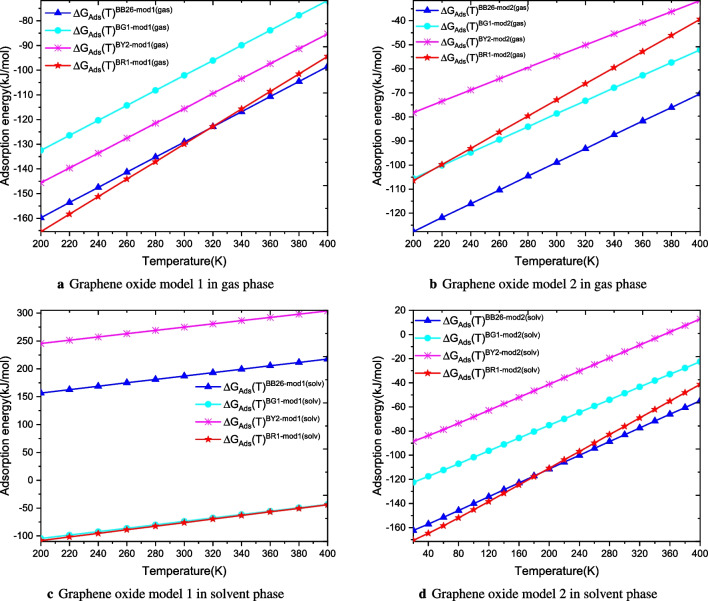
Free energies of adsorption of dyes onto graphene oxide models in both gas and solvent phases for temperatures ranging from 200 to 400 K

### Temperature effects on adsorption free energy

Temperature effects on the adsorption free energy of dyes onto graphene oxide have been assessed in both gas and solvent phases. Thus, we calculated the adsorption free energy as a function of temperature. The adsorption free energies are calculated for temperatures ranging from 200 to 400 K The change of the adsorption free energy as a function of temperature in both gas and solvent phases is provided in Fig. 4. Examination of the results reveals two general remarks for the eight complexes in gas and solvent phases: (1) the adsorption free energy of a dye onto a graphene oxide is considerably affected by the change of the temperature; (2) it is noted that the adsorption free energy varies linearly as a function of temperature (see Fig. 4).

#### In the gas phase

It appears from the results that the free energy of adsorption varies from $-$159.8 kJ mol$^{-1}$ at 200 K to $-$98.5 kJ mol$^{-1}$ at 400 K for BB26 with the first model of graphene oxide, from $-$127.7 kJ mol$^{-1}$ at 200 K to $-$70.4 kJ mol$^{-1}$ at 400 K with the second model graphene oxide. A similar variation can be noted for other dyes (see Fig. 4). We can see that the adsorption energies of all dyes increase with increasing temperature (see Fig. 4). The values of the adsorption energy of the dyes indicate their ability to interact with the surface of the graphene oxide. The higher the adsorption energy value, the stronger the interaction between dye and adsorbent. These results highlight the importance of taking into account the entropic effects in the evaluation of adsorption of pollutants.

#### In the solvent phase

The calculations show the influence of the model of solvation on the free energy of adsorption. We can see that adsorption free energies are affected significantly when switching from the gas phase to the solvent phase. It is important to note the important difference between the adsorption free energy in gas and solvent phases at different temperatures (see Fig. 4). As can be seen in Fig. 4, the plot of the adsorption free energy shows that it increases linearly with temperature. Thus, the adsorption process is entropic [54]. Therefore, it becomes crucial to consider the effects of temperature and solvent in adsorption. The adsorption energy must be evaluated using the Gibbs free energy as mentioned in our previous works [53, 54]. Elsewhere, it is found that the adsorption energy becomes positive above some temperatures. The positive value of the adsorption free energy indicates that the adsorption does not occur spontaneously [54]. Overall, the calculated adsorption energy values reveal that graphene oxide is an efficient adsorbent that removes dyes from wastewater.

### Partial conclusions and discussions

The study of the DFT-based descriptors of the dyes allowed us to conclude that the BB26 and BG1 dyes are expected to be more reactive than BY2 and BR1. This conclusion has been reported previously by de Souza and coworkers [7] at the B3LYP/6-31 G(d) level of theory. In addition, the QTAIM analysis performed in this work also allowed us to identify the non-covalent interactions between the dyes and the graphene oxides. It has been found that the non-covalent interactions comprise CH$\cdots$O and NH$\cdots$O hydrogen bondings, CH$\cdots \pi$, OH$\cdots \pi$, $\pi \cdots \pi$, O$\cdots$O, and H$\cdots$H bonding interactions. The QTAIM analysis has been performed in this work for the first time to understand the interaction between dyes and graphene oxides. After studying the complexes formed by the dyes and graphene oxides, we calculated the adsorption electronic energies and the adsorption free energies at different temperatures in both gas and solvent phases. It has been found that the adsorption electronic energy is overestimated as compared to the adsorption free energy at room temperature. This has been noted also in our previous works [53, 54]. Additionally, it has been found that the adsorption free energies calculated in the gas phase are overestimated as compared to those calculated in the solvent phase. Similar observations were made for the adsorption electronic energies. Thus, adsorption energies calculated generally in the literature are doubly overestimated: (1) overestimation from the use of electronic energy instead of Gibbs free energy and (2) overestimation from the use of gas phase instead of solvent phase. Consequently, most of the conclusions are wrongly drawn.

Exploration of the literature shows that no previous computational works have been reported about the adsorption of the four dyes studied in this work onto graphene oxide. However, experimental works have been reported previously. The adsorption of BB26 onto graphene oxide has been studied experimentally by Martins et al. [55] and de Figueiredo Neves et al. [56]. In addition, adsorption of basic red 1 (BR1) onto graphene oxide has been reported previously using experimental methods [57]. On the other hand, graphene oxide has been assessed for the adsorption of dyes using density functional theory [58–62]. Justino et al. [62] calculated the adsorption electronic energy of methylene blue and indigo carmine onto three models of graphene oxides. The adsorption electronic energies vary from $-$92.0 to $-$187.0 kJ mol$^{-1}$ and from $-$55.0 to $-$156.0 kJ mol$^{-1}$ for methylene blue and indigo carmine, respectively, depending on the graphene oxide models [62]. These values are in the same range as the adsorption electronic energies obtained in this work for BB26, BG1, BY2, and BR1 (see Table 2).

## Summary and perspectives

The aim of this study was to evaluate the adsorption performance of two models of graphene oxide for the elimination of four dyes using computational chemistry. Thus, we studied the adsorption of dyes (BB26, BG1, BY2, and BR1) onto two models of graphene oxide at the PW6B95-D3/def2-SVP level of theory. We started by calculating some DFT-based descriptors of the dyes to predict their reactivities based on the frontier molecular orbital energies. Then, a QTAIM analysis has been performed. This allowed us to understand the interactions between the dyes and the graphene oxides. It has been found that the CH$\cdots$O and OH$\cdots$O hydrogen bondings are the strongest non-covalent interactions in the systems. Finally, the adsorption free energies are calculated. We assessed solvent and temperature’s effects on adsorption’s free energy. The results show that the solvent effects considerably change the predicted value of the adsorption free energy. It has been found that the adsorption free energy calculated in the gas phase is overestimated. Similarly, we noted that the adsorption energy calculated using the electronic energy is also overestimated. We concluded that the calculation of the adsorption energy should be performed using the free energy in the solvent phase. Overall, this study shows that both models of graphene oxide are efficient in the adsorption of dyes.

This work lays a route for further theoretical investigations of graphene oxide as an adsorbent for emerging pollutants. As an introduction, this work has provided sufficient data to support the conclusion that graphene oxide can be a promising adsorbent for cationic dyes. Nevertheless, this work has limitations that need improvements in future studies. These limitations include the following:

- **Conformational search:** The studied dyes have several conformers that could coexist in the solvent phase. A complete study considering the contribution of different conformers of the dyes would be closer to reality.
- **Configurational search of the complex:** The complex formed by the interaction of a dye with a graphene oxide model has several configurations with different relative energies. The global and local minima configurations should be considered for accuracy, weighted by their Boltzmann probabilities. It is important to state that this will be an expensive investigation necessary for accuracy.
- **Environmental effects:** In the current study, only implicit solvation has been considered to consider the environmental effects. A more accurate study including a few explicit water molecules would be welcome in the literature. Similar to the previous perspective, the current suggestion is foreseen to be an expensive procedure necessary for accuracy. Boltzmann weights should be used to consider the contribution of all possible configurations.

### Supplementary Information

Below is the link to the electronic supplementary material.

Supplementary file 1 (pdf 2976 KB)

Supplementary file 2 (pdf 146 KB)

## Data Availability

The data used in this work is provided in the manuscript or in the supporting information.
