# Supplementary material for: Adsorption of some cationic dyes onto two models of graphene oxide
Source: J Mol Model. 2023 Nov 18;29(12):380. doi: 10.1007/s00894-023-05761-8 (PMC10657294; doi:10.1007/s00894-023-05761-8)
Supplement: Supplementary file 1 — (pdf 2976 KB) [file 894_2023_5761_MOESM1_ESM.pdf]

Adsorption of Some Cationic Dyes Onto Two Models of Graphene Oxide

Emma Mounra[°], Alhadji Malloum[†,◇,*], Jean Jules Fifen[°], and Jeanet Conradie[†,‡]

[°] Department of Physics, Faculty of Science, University of Ngaoundere, PO BOX 454, Ngaoundere, Cameroon.

[†] Department of Chemistry, University of the Free State, PO BOX 339, Bloemfontein 9300, South Africa.

[◇] Department of Physics, Faculty of Science, University of Maroua, PO BOX 46, Maroua, Cameroon.

[‡] Department of Chemistry, UiT - The Arctic University of Norway, N-9037 Tromsø, Norway.

August 25, 2023

SUPPORTING INFORMATION:

1 Definition of dye

The dye is defined as a product capable of dyeing a substance in a durable way. They have groups that give them color: they are called chromophores and groups that allow them to attach to chromophores. These groups are capable of transforming white light in the visible spectrum (from 380 to 750 nm) into colored light or by reflection on a body by transmission or diffusion[1].

1.1 Synthetic dyes

They are made from man-made chemicals. They are used in many consumer products such as clothing, cosmetics and textiles. From these raw materials, the intermediates are made by a series of chemical processes which, in general, correspond to the replacement of one or more hydrogen atoms of the starting material by particular elements or radicals[2]. These are compounds having molecules such as benzene as their basic structure.

Structure plays an important role in determining the coloring properties of organic compounds. In general, a typical dye molecule consists of three parts: a chromophore, an auxochrome group, and a solubilizing group. The chromophore is somehow the portion responsible for the color of the compound. The auxochrome is the part influencing the intensity of the coloring and it effectively fixes the dye on the support and finally the solubilizing group improves the solubility of the dye and thus, it can potentially be applied in aqueous medium[3]. Some types of dyes are studied in our work: see table1

1.2 Environmental effects of dyes

Dyes can be toxic to living organisms if not properly removed, which can lead to ecosystem disturbances and death of living organisms. They can also cause water and soil pollution when released into industrial wastewater. They can also have a negative impact on human health, with some dyes being classified as carcinogenic[4,5]. The production of certain dyes can also have a negative impact on the environment due to the use of toxic chemicals such as organic solvents and heavy metals.

2 The adsorption mechanism of a dye

Adsorption is a process, widely answered for dye removal also has wide applicability in wastewater treatment[6]. Separation by adsorption is based on selective adsorption (thermodynamic and/or kinetic) of pollutants (called adsorbate) by an adsorbent, thanks to specific interactions between the surface of the material and the adsorbed products[7] it is a simple mass transfer from liquid phase towards the surface of the solid, this process takes place in several stages.

External diffusion: corresponds to the transfer of the solute (a dye) from within the solution to the external surface of the grains. The transfer of external matter depends on the hydrodynamic conditions of the flow of a fluid in an adsorbent bed.

Internal diffusion: the fluid particles penetrate inside the pores. It depends on the concentration gradient of the solute. Diffusion of the surface in contact with the active sites, it corresponds to the fixing of the molecules on the pore surface.

* *E-mail: MalloumA@ufs.ac.za; Tel: +237 695 15 10 56*

Table S1 Studied dyes and their chemical structures.

Dyes	Abbreviations	Chemical structures	Formula	Class
Basic Blue 26 (Victory Blue)	BB26	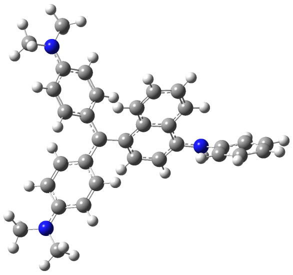	C33H32N3(1+)	Triarylmethane
Basic Green 1 (Bright Green)	BG1	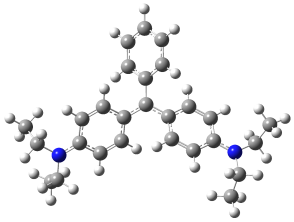	C27H33N2(1+)	Triarylmethane
Basic Yellow 2 (Auramine)	BY2	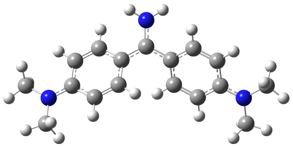	C17H22N3(1+)	Diphenylmethane
Basic Red 1 (Rhodamine 6G)	BR1	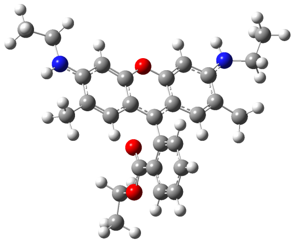	C28H31N2O3(1+)	Xanthene

3 The structure of graphene oxide

Compared to graphene, graphene oxide or GO is strongly oxygenated by hydroxyl and epoxide groups on sp^3 hybridized carbons in the basal plane, as well as carbonyl and carboxyl groups located at the edges of the sheets on the hybridized carbons sp^2 (figure 1).

Graphene oxide has many interesting physicochemical properties that make it an innovative material[8] :

Structure: Graphene oxide is composed of oxidized carbon layers, forming a sheet structure with oxygen atoms bonded to carbon atoms. It retains the sheet structure of graphene, but with a surface modified by the groups functional.

Conductivity: Although graphene oxide is an oxidized form of graphene and therefore less conductive, it still retains some electrical conductivity, which distinguishes it from other carbon oxides.

Thermal stability: Graphene oxide exhibits high thermal stability, making it resistant to high temperatures without degrading. This makes it a promising material for high temperature applications.

Reactive surface: The functional groups present on the surface of graphene oxide make it very chemically reactive. It can react with different molecules and be functionalized for specific applications.

Storage capacity: Graphene oxide has a high adsorption capacity, which means that it can absorb and hold different molecules on its surface. This makes it attractive for applications such as gas detection, water purification and energy storage.

4 functional hybrid used

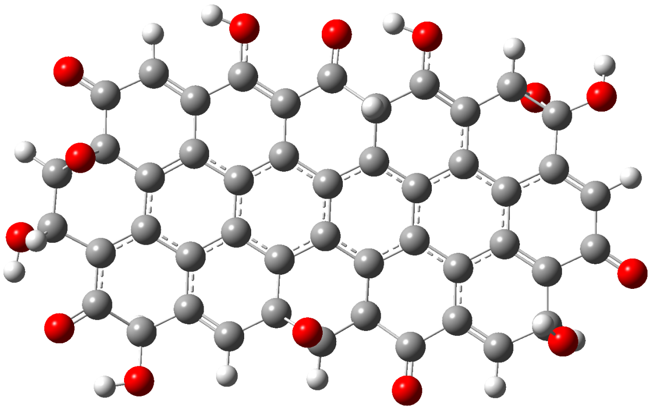

a model 1

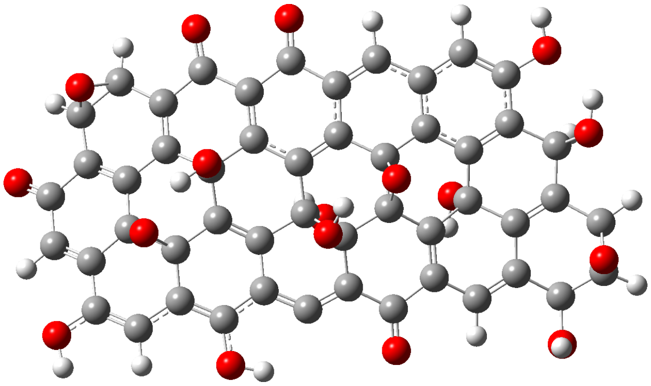

b model 2

Figure S1 Structure of graphene oxide

The functional PW6B95D3[9] refers to a specific combination of functionals in the density functional theory (DFT) method used in quantum chemistry. It combines the terms PW6 (perdew-wang 2006) and B95 (Becke 95) with the parameter D3 to include dispersion corrections according to the Grimme method. The PW6 functional is known for its good behavior towards electronic properties in various chemical systems, while the B95 functional offers a better description of ionization and electron affinity energies. Finally, the dispersion correction D3 makes it possible to account for dispersion interactions, which are essential for an accurate description of molecular systems.

Def2SVP[10] is a basis set (or slater basis) which is used to represent atomic orbitals in electrical calculations. This base set (double-zeta) includes two Gaussian functionals for internal electrons (core), four functional functions for valence electrons and two Gaussian functions for polarizable electrons. The interest of the PW6B95D3/def2SVP functional lies in the fact that it provides an accurate description of electronic interactions, taking into account both exchange and correlation interactions. It has been parameterized to be used with the SVP polarization deficient basis (def2SVP), which allows faster calculations while maintaining good precision.

5 Analysis of frontier orbitals

The energies of HOMO (Highest Occupied Molecular Orbital) and LUMO (Lowest Unoccupied Molecular Orbital) are very popular descriptors of quantum chemistry. energy of the HOMO orbital describes the ability of the molecule to donate electrons. Conversely, the energy of the LUMO orbital describes the ability of the molecule to accept electrons[11]. Frontier molecular orbitals play an important role in electrical, optical and chemical properties[12]. Figure 2 shows the HOMO and LUMO orbitals of the basic dyes tested.

References

1 M Auta and BH Hameed. Chitosan–clay composite as highly effective and low-cost adsorbent for batch and fixed-bed adsorption of methylene blue. *Chemical Engineering Journal*, 237:352–361, 2014.

2 Véronique Courilleau-Haverlant Mady Capon and Cécile Valette. Chimie des couleurs et des odeurs. *Cultures et techniques*, 1993.

3 Pradyutha ACh Ameena Husain and B Anupama. Dna binding affinities, anti-oxidant, antimicrobial and molecular docking activities of pd (ii) complexes of chromone schiff bases. *Journal of Molecular Structure*, 1254:132341, 2022.

4 Atul Kumar Mittal Nityanand Singh Maurya and Peter Cornel. Evaluation of adsorption potential of adsorbents : A case of uptake of cationic dyes. *Journal of Environmental Biology*, 29(1):31, 2007.

5 Mickael Le Bechec Sylvie Lacombe Jérôme Frayret Vanessa Peings, Albéric Andrin and Thierry Pigot. Couplage photocatalyse-oxydation par le ferrate (vi) pour le traitement du colorant rhodamine 6g. *Revue des Sciences de l'Eau*, 30(1):35–39, 2017.

6 Bahia MEROUFEL. *"Adsorption des polluants organiques et inorganiques sur des substances naturelles : Kaolin, racines de Calotropis procera et Noyaux de dattes",Science du bois et des fibres*. PhD thesis, Université de Lorraine, 2015.

7 Geetanjali Rathi Saif Ali Chaudhry Abdullah M Asiri et al. Nusrat Tara, Sharf I Siddiqui. Nano-engineered adsorbent for the removal of dyes from water : A review. *Current Analytical Chemistry*, 16(1):14–40, 2020.

8 Alexander D Todd Daniel R Dreyer and Christopher W Bielawski. Harnessing the chemistry of graphene oxide. *Chemical Society Reviews*, 43(15):5288–5301, 2014.

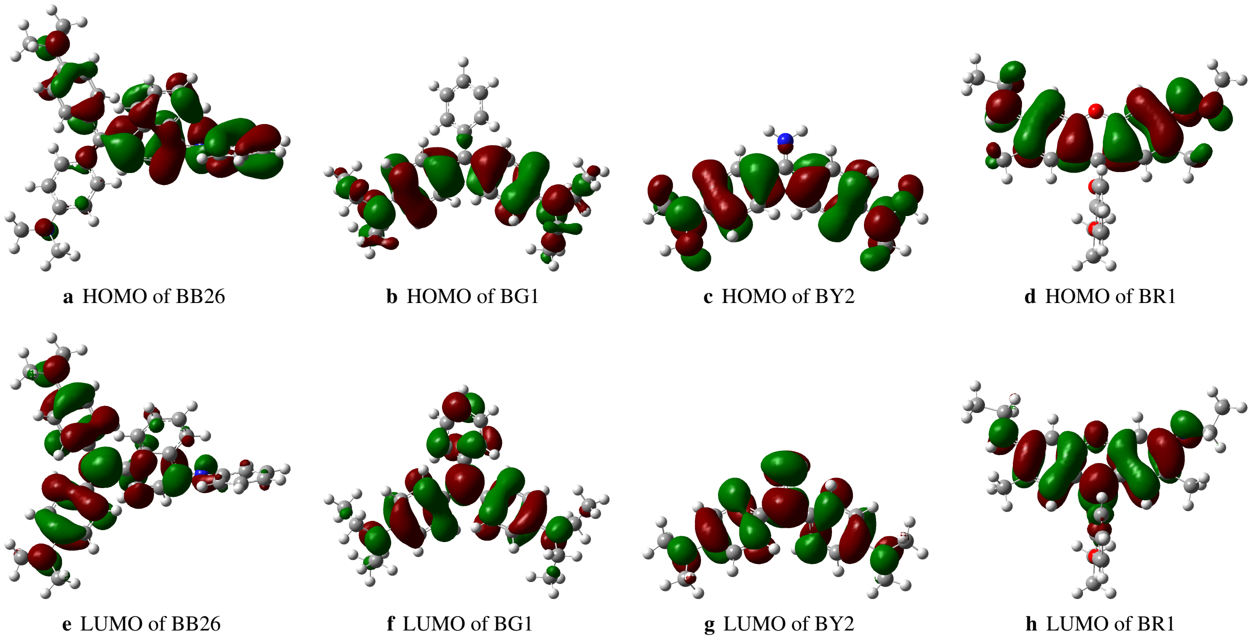

Figure S2 HOMO and LUMO frontier molecular orbitals of the four dyes investigated in this work at the PW6B95-D3/def2-SVP computational level of theory.

9 Yan Zhao and Donald G Truhlar. Design of density functionals that are broadly accurate for thermochemistry, thermochemical kinetics, and nonbonded interactions. *The Journal of Physical Chemistry A*, 109(25):5656–5667, 2005.

10 Enrico Tapavicza Raghunathan Ramakrishnan, Mia Hartmann and O Anatole Von Lilienfeld. Electronic spectra from tddft and machine learning in chemical space. *The Journal of chemical physics*, 2015.

11 Káthia Maria Honório and ABF Da Silva. An am1 study on the electron-donating and electron-accepting character of biomolecules. *International Journal of Quantum Chemistry*, 95(2):126–132, 2003.

12 Ian Fleming. Frontier orbitals and organic chemical reactions. *(No Title)*, 1976.
