# Supplementary material for: Adsorption of some cationic dyes onto two models of graphene oxide
Source: J Mol Model. 2023 Nov 18;29(12):380. doi: 10.1007/s00894-023-05761-8 (PMC10657294; doi:10.1007/s00894-023-05761-8)
Supplement: Supplementary file 2 — (pdf 146 KB) [file 894_2023_5761_MOESM2_ESM.pdf]

GO1 + BB26	Name	Atoms	Rho	DeltaRho	Ellipticity	K	BPL-GBL
1	BCP1	C1 - C2	0,2767	-0,7040	0,0619	0,2404	0,0004
2	BCP2	C2 - C3	0,3311	-0,8656	0,2653	0,3378	0,0002
3	BCP3	C3 - C4	0,2846	-0,6965	0,1094	0,2516	0,0003
4	BCP4	C1 - C5	0,2597	-0,6152	0,0608	0,2141	0,0011
5	BCP5	C4 - C6	0,2887	-0,7120	0,1225	0,2586	0,0001
6	BCP6	C5 - C6	0,3086	-0,7599	0,2269	0,2936	0,0001
7	BCP7	C9 - C124	0,0076	0,0239	2,8000	-0,0013	0,1439
8	BCP8	C3 - C7	0,2733	-0,6743	0,0956	0,2343	0,0004
9	BCP9	C7 - C8	0,2531	-0,5927	0,0523	0,2064	0,0005
10	BCP10	C9 - C10	0,3012	-0,7479	0,1815	0,2800	0,0002
11	BCP11	C4 - C10	0,2970	-0,7348	0,1618	0,2720	0,0002
12	BCP12	C8 - C9	0,2560	-0,5925	0,0387	0,2095	0,0006
13	BCP13	C7 - O37	0,4109	0,4467	0,0518	0,7182	0,0000
14	BCP14	C8 - C11	0,2609	-0,6199	0,0537	0,2154	0,0007
15	BCP15	C8 - H12	0,2649	-0,9346	0,0133	0,2772	0,0000
16	BCP16	C11 - C13	0,2448	-0,4724	0,2771	0,2060	0,0259
17	BCP17	C9 - C15	0,2986	-0,7390	0,1704	0,2753	0,0002
18	BCP18	C13 - C14	0,2718	-0,6616	0,0757	0,2301	0,0006
19	BCP19	C14 - C15	0,2981	-0,7377	0,1652	0,2738	0,0002
20	BCP20	C5 - C16	0,2844	-0,7058	0,1369	0,2516	0,0001
21	BCP21	C16 - C17	0,2897	-0,7385	0,1325	0,2601	0,0002
22	BCP22	C6 - C19	0,2832	-0,6848	0,1343	0,2493	0,0001
23	BCP23	C17 - C18	0,3251	-0,8358	0,2701	0,3270	0,0001
24	BCP24	C19 - C20	0,3052	-0,7555	0,2165	0,2872	0,0001
25	BCP25	C18 - C19	0,2857	-0,6925	0,1386	0,2531	0,0003
26	BCP26	C10 - C21	0,2834	-0,6841	0,1366	0,2494	0,0000
27	BCP27	C20 - C21	0,2853	-0,6945	0,1274	0,2522	0,0000
28	BCP28	C15 - C23	0,2866	-0,6959	0,1439	0,2548	0,0000
29	BCP29	C21 - C22	0,3101	-0,7720	0,2366	0,2960	0,0001
30	BCP30	C22 - C23	0,3000	-0,7421	0,1853	0,2774	0,0001
31	BCP31	C23 - C24	0,2948	-0,7205	0,1777	0,2684	0,0002
32	BCP32	C14 - C26	0,3032	-0,7580	0,1773	0,2831	0,0000
33	BCP33	C24 - C25	0,3054	-0,7575	0,2131	0,2873	0,0001
34	BCP34	C25 - C26	0,2867	-0,6983	0,1363	0,2546	0,0000
35	BCP35	C18 - C27	0,2712	-0,6766	0,0811	0,2293	0,0002
36	BCP36	C20 - C29	0,2834	-0,6830	0,1393	0,2502	0,0001
37	BCP37	C27 - O56	0,2721	-0,4520	0,4967	0,3788	0,0050
38	BCP38	C27 - C28	0,2612	-0,5545	0,2365	0,2270	0,0343
39	BCP39	C28 - O56	0,2447	-0,2883	0,7869	0,2984	0,0038
40	BCP40	C28 - C29	0,2715	-0,6545	0,0737	0,2300	0,0007
41	BCP41	C22 - C31	0,2585	-0,6004	0,0521	0,2127	0,0005
42	BCP42	C29 - C30	0,3280	-0,8599	0,3611	0,3362	0,0002
43	BCP43	C30 - C31	0,2634	-0,6304	0,0777	0,2197	0,0014
44	BCP44	C32 - C33	0,2797	-0,6858	0,1394	0,2442	0,0002
45	BCP45	C24 - C33	0,2755	-0,6516	0,1278	0,2373	0,0003
46	BCP46	C31 - C32	0,2511	-0,5811	0,0644	0,2038	0,0011
47	BCP47	C1 - O34	0,2779	-0,6268	0,0456	0,4009	0,0005
48	BCP48	C1 - H35	0,2629	-0,9215	0,0382	0,2705	0,0002
49	BCP49	C16 - O38	0,3816	0,0647	0,0503	0,6616	0,0000
50	BCP50	O34 - H36	0,3299	-2,3342	0,0199	0,6511	0,0003
51	BCP51	H36 - O38	0,0436	0,1419	0,0112	0,0003	0,0106

52	BCP52	C17 - H39	0,2833	-1,1060	0,0121	0,3109	0,0000
53	BCP53	O40 - H44	0,0548	0,1712	0,0120	0,0035	0,0069
54	BCP54	C32 - O40	0,3988	0,3137	0,0512	0,6942	0,0001
55	BCP55	O40 - O79	0,0177	0,0741	0,0833	-0,0019	0,0112
56	BCP56	C11 - O41	0,2704	-0,3561	0,3972	0,3925	0,0047
57	BCP57	C13 - O41	0,2389	-0,2633	0,7853	0,2749	0,0039
58	BCP58	C13 - C51	0,2807	-0,7019	0,0847	0,2462	0,0003
59	BCP59	C11 - H42	0,2867	-1,1331	0,0316	0,3154	0,0002
60	BCP60	C30 - O43	0,3160	-0,2303	0,0165	0,5142	0,0011
61	BCP61	O43 - H44	0,3189	-2,2414	0,0135	0,6282	0,0007
62	BCP62	C26 - C45	0,2962	-0,7277	0,1698	0,2705	0,0000
63	BCP63	C45 - C46	0,2896	-0,7089	0,1386	0,2595	0,0002
64	BCP64	C25 - C48	0,2919	-0,7142	0,1597	0,2635	0,0003
65	BCP65	C47 - C48	0,3135	-0,7837	0,2481	0,3021	0,0000
66	BCP66	C48 - C50	0,2752	-0,6622	0,1004	0,2373	0,0001
67	BCP67	C46 - C47	0,2876	-0,7042	0,1278	0,2565	0,0001
68	BCP68	C33 - C49	0,3168	-0,8157	0,3053	0,3128	0,0007
69	BCP69	C49 - O79	0,3160	-0,1529	0,0122	0,5133	0,0014
70	BCP70	C49 - C50	0,2795	-0,6949	0,1106	0,2437	0,0008
71	BCP71	C45 - C52	0,2839	-0,6912	0,1147	0,2505	0,0002
72	BCP72	C51 - C52	0,3331	-0,8650	0,3165	0,3428	0,0002
73	BCP73	C51 - H54	0,2826	-1,0975	0,0161	0,3095	0,0004
74	BCP74	C2 - H53	0,2845	-1,1317	0,0052	0,3143	0,0001
75	BCP75	C2 - N133	0,0065	0,0184	0,3173	-0,0006	0,1270
76	BCP76	C31 - H55	0,2619	-0,9189	0,0105	0,2740	0,0000
77	BCP77	C29 - H117	0,0070	0,0223	2,7017	-0,0010	0,0555
78	BCP78	C28 - H57	0,2845	-1,0940	0,0417	0,3091	0,0003
79	BCP79	C27 - O58	0,2940	-0,5214	0,1383	0,4568	0,0018
80	BCP80	O58 - H59	0,3533	-2,3852	0,0213	0,6638	0,0008
81	BCP81	C60 - H74	0,2689	-0,9530	0,0512	0,2797	0,0001
82	BCP82	C52 - C60	0,2629	-0,6377	0,0793	0,2188	0,0010
83	BCP83	C46 - C62	0,3018	-0,7314	0,2374	0,2840	0,0001
84	BCP84	C60 - O72	0,2802	-0,5309	0,0393	0,4195	0,0005
85	BCP85	C60 - C61	0,2625	-0,6407	0,0729	0,2182	0,0014
86	BCP86	O66 - O67	0,0113	0,0410	0,9184	-0,0005	0,4084
87	BCP87	C61 - C62	0,2953	-0,7357	0,1863	0,2701	0,0000
88	BCP88	C63 - C64	0,2535	-0,5193	0,2189	0,2185	0,0295
89	BCP89	C47 - C63	0,2727	-0,6626	0,0648	0,2316	0,0004
90	BCP90	C25 - C95	0,0075	0,0214	0,1770	-0,0009	0,0580
91	BCP91	C62 - C65	0,2583	-0,6058	0,0262	0,2121	0,0007
92	BCP92	C64 - H71	0,2864	-1,1448	0,0286	0,3164	0,0003
93	BCP93	C64 - C65	0,2674	-0,6705	0,0864	0,2251	0,0011
94	BCP94	C61 - O66	0,3886	0,2220	0,0255	0,6735	0,0000
95	BCP95	C65 - O67	0,2683	-0,5497	0,0337	0,3832	0,0006
96	BCP96	O67 - H69	0,3556	-2,3753	0,0229	0,6627	0,0005
97	BCP97	C65 - H68	0,2792	-1,0350	0,0323	0,2973	0,0002
98	BCP98	C63 - O70	0,2366	-0,2358	0,9194	0,2656	0,0042
99	BCP99	C63 - C76	0,2650	-0,6518	0,0655	0,2207	0,0002
100	BCP100	C64 - O70	0,2574	-0,2679	0,4837	0,3628	0,0047
101	BCP101	O66 - H73	0,0249	0,0885	0,6976	0,0000	0,1553
102	BCP102	O72 - H73	0,3456	-2,3438	0,0226	0,6532	0,0004
103	BCP103	H78 - H80	0,0208	0,0673	0,4514	-0,0006	0,3244
104	BCP104	C50 - C75	0,3230	-0,8199	0,2781	0,3226	0,0000

105	BCP105	C75 - C76	0,2826	-0,7095	0,1156	0,2492	0,0002
106	BCP106	C76 - O77	0,4082	0,4210	0,0731	0,7123	0,0000
107	BCP107	C75 - H78	0,2779	-1,0256	0,0137	0,2973	0,0003
108	BCP108	O79 - H80	0,3559	-2,4442	0,0143	0,6781	0,0009
109	BCP109	C91 - C94	0,2857	-0,7054	0,1489	0,2544	0,0008
110	BCP110	C81 - C82	0,3086	-0,7837	0,2085	0,2947	0,0000
111	BCP111	C82 - C83	0,3101	-0,7854	0,2249	0,2978	0,0000
112	BCP112	O66 - H108	0,0100	0,0296	0,2214	-0,0001	0,0136
113	BCP113	C81 - C84	0,3098	-0,7886	0,2109	0,2969	0,0000
114	BCP114	C62 - N97	0,0078	0,0258	0,4673	-0,0012	0,9556
115	BCP115	H68 - H108	0,0076	0,0261	0,1564	-0,0008	0,0368
116	BCP116	C84 - C85	0,3089	-0,7824	0,2138	0,2954	0,0000
117	BCP117	C83 - C86	0,3069	-0,7734	0,2479	0,2920	0,0012
118	BCP118	C86 - N97	0,2790	-0,7822	0,0539	0,3970	0,0005
119	BCP119	C85 - C86	0,3092	-0,7861	0,2489	0,2963	0,0009
120	BCP120	C87 - C88	0,3024	-0,7675	0,1689	0,2832	0,0000
121	BCP121	C88 - C89	0,3181	-0,8174	0,2493	0,3131	0,0002
122	BCP122	C51 - C90	0,0027	0,0063	0,0567	-0,0003	0,2589
123	BCP123	C86 - H104	0,0129	0,0504	0,1205	-0,0022	0,1872
124	BCP124	C87 - C90	0,3193	-0,8249	0,2432	0,3152	0,0001
125	BCP125	C91 - C92	0,2913	-0,7036	0,1776	0,2624	0,0004
126	BCP126	C90 - C91	0,2961	-0,7308	0,1715	0,2719	0,0004
127	BCP127	C89 - C92	0,2967	-0,7348	0,1736	0,2730	0,0003
128	BCP128	C92 - C93	0,2771	-0,6557	0,1407	0,2399	0,0004
129	BCP129	O70 - H98	0,0169	0,0479	0,0458	-0,0001	0,0048
130	BCP130	C94 - N97	0,3199	-0,9360	0,0931	0,5103	0,0011
131	BCP131	C48 - H99	0,0073	0,0240	0,7120	-0,0012	0,2922
132	BCP132	C94 - C95	0,3078	-0,7892	0,2195	0,2938	0,0006
133	BCP133	C93 - C96	0,3005	-0,7431	0,2022	0,2797	0,0003
134	BCP134	C95 - C96	0,3126	-0,7967	0,2353	0,3028	0,0002
135	BCP135	N97 - H98	0,3296	-1,6031	0,0379	0,4449	0,0001
136	BCP136	C95 - H99	0,2794	-1,0398	0,0235	0,3006	0,0000
137	BCP137	C96 - H100	0,2827	-1,0727	0,0083	0,3064	0,0000
138	BCP138	C88 - H101	0,2812	-1,0626	0,0093	0,3036	0,0000
139	BCP139	C89 - C124	0,0131	0,0578	7,2598	-0,0033	0,5911
140	BCP140	C89 - H102	0,2835	-1,0766	0,0147	0,3084	0,0001
141	BCP141	C87 - H103	0,2815	-1,0680	0,0114	0,3046	0,0000
142	BCP142	C90 - H104	0,2840	-1,0944	0,0107	0,3104	0,0001
143	BCP143	C83 - H105	0,2798	-1,0446	0,0153	0,3011	0,0000
144	BCP144	C81 - H106	0,2808	-1,0584	0,0131	0,3030	0,0000
145	BCP145	C82 - H107	0,2807	-1,0567	0,0112	0,3026	0,0000
146	BCP146	C85 - H108	0,2825	-1,0778	0,0158	0,3076	0,0001
147	BCP147	C84 - H109	0,2813	-1,0651	0,0105	0,3040	0,0000
148	BCP148	C93 - C110	0,2782	-0,6591	0,1453	0,2421	0,0004
149	BCP149	H55 - C112	0,0139	0,0402	0,1538	-0,0009	0,0254
150	BCP150	C110 - C111	0,2795	-0,6706	0,1304	0,2436	0,0000
151	BCP151	C111 - C112	0,2980	-0,7370	0,1829	0,2754	0,0004
152	BCP152	O43 - H144	0,0032	0,0130	0,2476	-0,0007	0,0144
153	BCP153	C112 - C113	0,3161	-0,8040	0,2595	0,3095	0,0003
154	BCP154	C111 - C114	0,2969	-0,7340	0,1779	0,2733	0,0004
155	BCP155	C114 - C115	0,3167	-0,8071	0,2591	0,3103	0,0003
156	BCP156	C113 - C116	0,2957	-0,7383	0,1848	0,2724	0,0006
157	BCP157	C115 - C116	0,2950	-0,7358	0,1837	0,2714	0,0006

158	BCP158	C112 - H130	0,0137	0,0646	2,2900	-0,0037	0,6157
159	BCP159	C112 - H117	0,2842	-1,0906	0,0091	0,3103	0,0000
160	BCP160	C113 - H118	0,2819	-1,0589	0,0243	0,3055	0,0001
161	BCP161	C114 - H119	0,2830	-1,0850	0,0075	0,3078	0,0000
162	BCP162	C115 - H120	0,2819	-1,0581	0,0234	0,3053	0,0001
163	BCP163	C110 - C121	0,2860	-0,6898	0,1613	0,2547	0,0000
164	BCP164	C121 - C122	0,2936	-0,7232	0,1622	0,2675	0,0003
165	BCP165	C16 - H137	0,0051	0,0180	11,3897	-0,0010	0,4781
166	BCP166	C122 - C123	0,3187	-0,8158	0,2641	0,3142	0,0004
167	BCP167	C3 - C125	0,0079	0,0243	1,2356	-0,0012	0,0150
168	BCP168	C2 - H136	0,0071	0,0291	0,1774	-0,0019	0,2960
169	BCP169	C7 - H128	0,0082	0,0313	1,7319	-0,0018	0,1991
170	BCP170	O41 - H127	0,0196	0,0625	0,0266	-0,0004	0,0153
171	BCP171	C121 - C124	0,2970	-0,7416	0,1528	0,2731	0,0001
172	BCP172	O37 - H136	0,0070	0,0228	0,0542	-0,0004	0,0176
173	BCP173	C123 - C126	0,2930	-0,7310	0,1711	0,2681	0,0007
174	BCP174	C124 - C125	0,3207	-0,8235	0,2641	0,3186	0,0006
175	BCP175	O34 - H135	0,0091	0,0335	0,1439	-0,0009	0,0461
176	BCP176	O34 - H138	0,0084	0,0366	0,8850	-0,0017	0,7382
177	BCP177	C125 - C126	0,2931	-0,7329	0,1692	0,2687	0,0008
178	BCP178	C124 - H127	0,2916	-1,1738	0,0053	0,3277	0,0001
179	BCP179	C125 - H128	0,2822	-1,0607	0,0255	0,3061	0,0002
180	BCP180	H128 - H136	0,0155	0,0576	0,4816	-0,0019	0,2504
181	BCP181	C123 - H129	0,2828	-1,0679	0,0232	0,3073	0,0001
182	BCP182	C122 - H130	0,2844	-1,0926	0,0061	0,3103	0,0001
183	BCP183	C132 - N133	0,2619	-0,7047	0,0245	0,3430	0,0001
184	BCP184	C126 - N133	0,3270	-0,8729	0,1119	0,5365	0,0004
185	BCP185	C131 - N133	0,2609	-0,6951	0,0339	0,3430	0,0001
186	BCP186	C132 - H139	0,2759	-1,0152	0,0365	0,2938	0,0006
187	BCP187	C132 - H138	0,2837	-1,0955	0,0286	0,3107	0,0007
188	BCP188	C131 - H134	0,2747	-1,0102	0,0379	0,2919	0,0006
189	BCP189	C131 - H135	0,2827	-1,0932	0,0310	0,3095	0,0005
190	BCP190	C131 - H136	0,2818	-1,0692	0,0337	0,3062	0,0007
191	BCP191	C132 - H137	0,2791	-1,0503	0,0347	0,3008	0,0006
192	BCP192	N140 - C141	0,2648	-0,7280	0,0393	0,3455	0,0000
193	BCP193	C116 - N140	0,3237	-0,8885	0,1155	0,5247	0,0000
194	BCP194	C145 - H148	0,2768	-1,0262	0,0360	0,2957	0,0005
195	BCP195	C141 - H142	0,2763	-1,0205	0,0368	0,2946	0,0005
196	BCP196	C141 - H143	0,2812	-1,0657	0,0311	0,3050	0,0007
197	BCP197	C141 - H144	0,2772	-1,0308	0,0352	0,2966	0,0005
198	BCP198	N140 - C145	0,2654	-0,7315	0,0385	0,3468	0,0000
199	BCP199	C145 - H147	0,2813	-1,0669	0,0309	0,3052	0,0007
200	BCP200	C145 - H146	0,2764	-1,0202	0,0365	0,2946	0,0005

GO1 + BG1

1	BCP1	C1 - C2	0,2757	-0,6999	0,0580	0,2388	0,0007
2	BCP2	C3 - C4	0,2833	-0,6897	0,1127	0,2496	0,0003
3	BCP3	C2 - C3	0,3297	-0,8571	0,2649	0,3348	0,0001
4	BCP4	C1 - C5	0,2638	-0,6336	0,0481	0,2197	0,0007
5	BCP5	C1 - H35	0,2666	-0,9507	0,0308	0,2770	0,0002
6	BCP6	C4 - C6	0,2904	-0,7188	0,1248	0,2614	0,0001
7	BCP7	C4 - C10	0,2958	-0,7292	0,1591	0,2698	0,0003

8	BCP8	C5 - C16	0,2870	-0,7171	0,1393	0,2556	0,0001
9	BCP9	C5 - C6	0,3111	-0,7728	0,2271	0,2983	0,0001
10	BCP10	C3 - C7	0,2724	-0,6694	0,0969	0,2327	0,0003
11	BCP11	C7 - C8	0,2517	-0,5868	0,0541	0,2043	0,0007
12	BCP12	C9 - C10	0,3031	-0,7573	0,1804	0,2832	0,0001
13	BCP13	C8 - C9	0,2578	-0,6006	0,0368	0,2121	0,0007
14	BCP14	C8 - C11	0,2620	-0,6240	0,0555	0,2170	0,0009
15	BCP15	C8 - H12	0,2650	-0,9338	0,0133	0,2774	0,0000
16	BCP16	C11 - C13	0,2461	-0,4799	0,2694	0,2079	0,0253
17	BCP17	C9 - C15	0,2972	-0,7334	0,1671	0,2728	0,0002
18	BCP18	C15 - C119	0,0068	0,0239	5,8808	-0,0014	0,5178
19	BCP19	C13 - C14	0,2725	-0,6631	0,0773	0,2311	0,0004
20	BCP20	C14 - C15	0,2988	-0,7408	0,1663	0,2751	0,0001
21	BCP21	C16 - O38	0,3829	0,0760	0,0518	0,6644	0,0000
22	BCP22	C6 - C19	0,2842	-0,6901	0,1320	0,2508	0,0001
23	BCP23	C16 - C17	0,2888	-0,7352	0,1309	0,2587	0,0001
24	BCP24	C18 - C19	0,2861	-0,6933	0,1421	0,2539	0,0002
25	BCP25	C17 - C18	0,3232	-0,8265	0,2685	0,3234	0,0001
26	BCP26	C19 - C20	0,3057	-0,7576	0,2171	0,2882	0,0001
27	BCP27	C10 - C21	0,2841	-0,6874	0,1370	0,2506	0,0000
28	BCP28	C20 - C21	0,2861	-0,6967	0,1317	0,2537	0,0002
29	BCP29	C15 - C23	0,2852	-0,6893	0,1455	0,2526	0,0000
30	BCP30	C21 - C22	0,3092	-0,7688	0,2348	0,2945	0,0001
31	BCP31	C22 - C23	0,3002	-0,7409	0,1925	0,2780	0,0001
32	BCP32	C23 - C24	0,2929	-0,7134	0,1743	0,2653	0,0002
33	BCP33	C14 - C26	0,3026	-0,7565	0,1736	0,2821	0,0001
34	BCP34	C25 - C26	0,2865	-0,6965	0,1396	0,2544	0,0000
35	BCP35	C24 - C25	0,3060	-0,7576	0,2209	0,2883	0,0001
36	BCP36	C18 - C27	0,2713	-0,6763	0,0852	0,2297	0,0002
37	BCP37	C21 - H103	0,0079	0,0282	3,0278	-0,0015	0,4945
38	BCP38	C27 - O58	0,2909	-0,5080	0,1381	0,4497	0,0017
39	BCP39	C20 - C29	0,2851	-0,6899	0,1416	0,2530	0,0001
40	BCP40	C28 - O56	0,2431	-0,2796	0,8011	0,2943	0,0040
41	BCP41	C27 - C28	0,2594	-0,5450	0,2427	0,2247	0,0339
42	BCP42	C28 - H57	0,2850	-1,1033	0,0410	0,3107	0,0003
43	BCP43	C28 - C29	0,2720	-0,6559	0,0803	0,2309	0,0006
44	BCP44	C29 - C30	0,3273	-0,8577	0,3609	0,3355	0,0002
45	BCP45	C22 - C31	0,2574	-0,5978	0,0468	0,2111	0,0009
46	BCP46	C32 - C33	0,2816	-0,6927	0,1465	0,2473	0,0002
47	BCP47	C30 - C31	0,2578	-0,6082	0,0733	0,2119	0,0011
48	BCP48	C24 - C33	0,2757	-0,6542	0,1238	0,2375	0,0001
49	BCP49	C31 - C32	0,2513	-0,5835	0,0620	0,2038	0,0009
50	BCP50	O40 - H44	0,0603	0,1815	0,0125	0,0063	0,0060
51	BCP51	O40 - O79	0,0193	0,0838	0,1047	-0,0024	0,0107
52	BCP52	C1 - O34	0,2724	-0,6126	0,0427	0,3838	0,0005
53	BCP53	H36 - O38	0,0303	0,0942	0,0267	0,0002	0,0253
54	BCP54	O34 - H36	0,3417	-2,4072	0,0210	0,6678	0,0004
55	BCP55	C7 - O37	0,4120	0,4639	0,0576	0,7201	0,0000
56	BCP56	C17 - H39	0,2829	-1,0965	0,0123	0,3094	0,0000
57	BCP57	C32 - O40	0,3959	0,2790	0,0462	0,6890	0,0001
58	BCP58	C11 - O41	0,2711	-0,3496	0,3905	0,3953	0,0049
59	BCP59	C13 - O41	0,2358	-0,2396	0,8565	0,2606	0,0043
60	BCP60	C51 - C52	0,3324	-0,8612	0,3172	0,3417	0,0002

61	BCP61	C11 - H42	0,2867	-1,1355	0,0311	0,3157	0,0002
62	BCP62	C30 - O43	0,3205	-0,2105	0,0164	0,5246	0,0012
63	BCP63	O43 - H44	0,3168	-2,2097	0,0128	0,6219	0,0006
64	BCP64	C45 - C46	0,2873	-0,7004	0,1323	0,2557	0,0002
65	BCP65	C26 - C45	0,2970	-0,7289	0,1755	0,2719	0,0000
66	BCP66	C47 - C48	0,3136	-0,7822	0,2528	0,3024	0,0000
67	BCP67	C25 - C48	0,2901	-0,7073	0,1580	0,2607	0,0002
68	BCP68	C48 - C50	0,2755	-0,6640	0,0994	0,2378	0,0001
69	BCP69	C46 - C47	0,2862	-0,6994	0,1225	0,2539	0,0001
70	BCP70	C49 - O79	0,3162	-0,1451	0,0119	0,5137	0,0013
71	BCP71	C33 - C49	0,3160	-0,8137	0,2987	0,3111	0,0006
72	BCP72	C50 - C75	0,3228	-0,8193	0,2764	0,3222	0,0000
73	BCP73	C49 - C50	0,2800	-0,6968	0,1115	0,2445	0,0008
74	BCP74	H78 - H80	0,0217	0,0686	0,4228	-0,0004	0,3000
75	BCP75	C51 - H54	0,2824	-1,0974	0,0171	0,3094	0,0004
76	BCP76	C13 - C51	0,2803	-0,6986	0,0842	0,2456	0,0005
77	BCP77	C52 - C60	0,2631	-0,6376	0,0845	0,2194	0,0011
78	BCP78	C45 - C52	0,2839	-0,6916	0,1127	0,2507	0,0002
79	BCP79	C2 - H53	0,2841	-1,1300	0,0048	0,3138	0,0001
80	BCP80	C31 - H55	0,2616	-0,9070	0,0113	0,2719	0,0000
81	BCP81	C27 - O56	0,2752	-0,4651	0,4776	0,3887	0,0051
82	BCP82	O58 - H59	0,3529	-2,3939	0,0212	0,6656	0,0007
83	BCP83	C60 - O72	0,2825	-0,5284	0,0350	0,4260	0,0005
84	BCP84	C46 - C62	0,3048	-0,7427	0,2425	0,2891	0,0001
85	BCP85	C60 - C61	0,2613	-0,6342	0,0762	0,2166	0,0014
86	BCP86	C61 - C62	0,2924	-0,7280	0,1675	0,2650	0,0001
87	BCP87	O66 - H69	0,0145	0,0473	0,1849	0,0001	0,1564
88	BCP88	C63 - C64	0,2511	-0,5045	0,2414	0,2150	0,0292
89	BCP89	C47 - C63	0,2724	-0,6609	0,0649	0,2310	0,0004
90	BCP90	C63 - C76	0,2659	-0,6546	0,0660	0,2219	0,0003
91	BCP91	C62 - C65	0,2586	-0,6086	0,0331	0,2126	0,0007
92	BCP92	C25 - C120	0,0066	0,0181	5,5749	-0,0008	0,4190
93	BCP93	C65 - O67	0,2697	-0,5585	0,0336	0,3864	0,0006
94	BCP94	C64 - C65	0,2661	-0,6644	0,0857	0,2229	0,0011
95	BCP95	C61 - O66	0,3921	0,2684	0,0306	0,6799	0,0000
96	BCP96	O67 - H69	0,3548	-2,3967	0,0227	0,6676	0,0005
97	BCP97	C65 - H68	0,2786	-1,0347	0,0330	0,2967	0,0002
98	BCP98	C48 - H138	0,0100	0,0298	1,3466	-0,0010	0,5261
99	BCP99	C63 - O70	0,2400	-0,2601	0,8389	0,2732	0,0031
100	BCP100	C64 - O70	0,2617	-0,2861	0,4352	0,3742	0,0046
101	BCP101	C64 - H71	0,2860	-1,1400	0,0285	0,3154	0,0002
102	BCP102	O72 - H73	0,3454	-2,3454	0,0226	0,6534	0,0004
103	BCP103	O66 - H73	0,0247	0,0879	0,7026	-0,0001	0,1543
104	BCP104	C60 - H74	0,2683	-0,9496	0,0532	0,2788	0,0001
105	BCP105	C76 - O77	0,4080	0,4192	0,0714	0,7119	0,0000
106	BCP106	C75 - C76	0,2820	-0,7073	0,1139	0,2484	0,0002
107	BCP107	C75 - H78	0,2774	-1,0194	0,0137	0,2962	0,0003
108	BCP108	O79 - H80	0,3560	-2,4404	0,0142	0,6775	0,0009
109	BCP109	O38 - H85	0,0083	0,0307	0,3812	-0,0010	0,1074
110	BCP110	O34 - H83	0,0049	0,0201	0,7895	-0,0010	0,0521
111	BCP111	C81 - C82	0,2490	-0,5793	0,0304	0,2024	0,0005
112	BCP112	C5 - C92	0,0020	0,0052	0,4821	-0,0002	0,3605
113	BCP113	C81 - H83	0,2744	-0,9955	0,0094	0,2919	0,0004

114	BCP114	C81 - H84	0,2730	-0,9870	0,0079	0,2899	0,0003
115	BCP115	H83 - H102	0,0089	0,0409	1,7161	-0,0025	0,6474
116	BCP116	C81 - H85	0,2757	-1,0157	0,0083	0,2951	0,0004
117	BCP117	C17 - H88	0,0054	0,0206	0,6012	-0,0012	0,3660
118	BCP118	O58 - H88	0,0110	0,0337	0,0699	-0,0002	0,0068
119	BCP119	C86 - C87	0,2498	-0,5824	0,0295	0,2031	0,0006
120	BCP120	C86 - H88	0,2778	-1,0362	0,0058	0,2998	0,0003
121	BCP121	C86 - H89	0,2728	-0,9779	0,0073	0,2888	0,0003
122	BCP122	C86 - H90	0,2730	-0,9850	0,0071	0,2899	0,0003
123	BCP123	H100 - H10[illegible]	0,0153	0,0599	0,5069	-0,0022	0,3540
124	BCP124	C91 - C92	0,2925	-0,7320	0,1628	0,2676	0,0006
125	BCP125	C82 - H99	0,2817	-1,0556	0,0306	0,3037	0,0006
126	BCP126	C91 - C96	0,2908	-0,7247	0,1614	0,2642	0,0008
127	BCP127	C82 - H98	0,2783	-1,0252	0,0339	0,2969	0,0007
128	BCP128	C91 - N97	0,3288	-0,8630	0,1166	0,5423	0,0001
129	BCP129	H98 - H102	0,0145	0,0644	0,8254	-0,0031	0,6240
130	BCP130	C92 - C93	0,3229	-0,8350	0,2694	0,3224	0,0006
131	BCP131	C94 - C112	0,2911	-0,7070	0,1741	0,2631	0,0001
132	BCP132	C93 - C94	0,2915	-0,7176	0,1514	0,2639	0,0002
133	BCP133	H55 - H103	0,0055	0,0208	0,4404	-0,0009	0,1172
134	BCP134	C94 - C95	0,2917	-0,7178	0,1549	0,2643	0,0004
135	BCP135	C82 - N97	0,2565	-0,6716	0,0239	0,3263	0,0001
136	BCP136	C96 - H104	0,2808	-1,0522	0,0229	0,3034	0,0002
137	BCP137	C87 - N97	0,2573	-0,6751	0,0385	0,3316	0,0002
138	BCP138	C95 - H103	0,2841	-1,0815	0,0073	0,3094	0,0001
139	BCP139	C95 - C96	0,3207	-0,8209	0,2821	0,3184	0,0004
140	BCP140	C87 - H100	0,2829	-1,0632	0,0300	0,3060	0,0006
141	BCP141	C87 - H101	0,2780	-1,0257	0,0330	0,2966	0,0007
142	BCP142	C92 - H102	0,2840	-1,0797	0,0211	0,3096	0,0002
143	BCP143	C93 - H105	0,2855	-1,1087	0,0063	0,3132	0,0001
144	BCP144	O41 - C110	0,0059	0,0200	3,0162	-0,0007	0,0641
145	BCP145	C110 - C11[illegible]	0,3023	-0,7529	0,1954	0,2830	0,0003
146	BCP146	C106 - C10[illegible]	0,3094	-0,7915	0,1941	0,2958	0,0000
147	BCP147	C111 - C11[illegible]	0,2716	-0,6546	0,0816	0,2317	0,0000
148	BCP148	C107 - C10[illegible]	0,3121	-0,7990	0,2153	0,3013	0,0000
149	BCP149	C3 - H118	0,0108	0,0326	0,0733	-0,0009	0,0176
150	BCP150	O37 - H117	0,0068	0,0264	0,6541	-0,0011	0,1822
151	BCP151	H105 - C11[illegible]	0,0138	0,0581	1,4444	-0,0031	0,6248
152	BCP152	C106 - C10[illegible]	0,3098	-0,7929	0,1951	0,2966	0,0000
153	BCP153	C109 - C11[illegible]	0,3115	-0,7954	0,2142	0,3004	0,0000
154	BCP154	C108 - C11[illegible]	0,3016	-0,7495	0,1948	0,2816	0,0005
155	BCP155	H103 - H12[illegible]	0,0129	0,0587	1,0092	-0,0033	0,7111
156	BCP156	H55 - C121	0,0070	0,0183	1,0139	-0,0006	0,4410
157	BCP157	C108 - H12[illegible]	0,0137	0,0596	1,5608	-0,0034	0,9508
158	BCP158	C112 - C11[illegible]	0,2861	-0,6912	0,1554	0,2547	0,0001
159	BCP159	C108 - H11[illegible]	0,2823	-1,0725	0,0112	0,3060	0,0000
160	BCP160	C106 - H11[illegible]	0,2822	-1,0786	0,0061	0,3060	0,0000
161	BCP161	C107 - H11[illegible]	0,2815	-1,0687	0,0108	0,3046	0,0000
162	BCP162	C109 - H11[illegible]	0,2823	-1,0801	0,0106	0,3066	0,0000
163	BCP163	C110 - H11[illegible]	0,2839	-1,0818	0,0136	0,3093	0,0000
164	BCP164	O41 - H124	0,0104	0,0329	0,0869	-0,0002	0,0157
165	BCP165	C113 - C11[illegible]	0,2929	-0,7218	0,1560	0,2662	0,0002
166	BCP166	C119 - C12[illegible]	0,3200	-0,8207	0,2659	0,3170	0,0005

167	BCP167	C113 - C121	0,2943	-0,7264	0,1655	0,2689	0,0003
168	BCP168	C121 - C122	0,3172	-0,8074	0,2666	0,3114	0,0004
169	BCP169	C120 - C123	0,2932	-0,7332	0,1709	0,2691	0,0007
170	BCP170	C122 - C123	0,2918	-0,7249	0,1718	0,2659	0,0007
171	BCP171	C119 - H124	0,2866	-1,1285	0,0060	0,3167	0,0001
172	BCP172	C120 - H125	0,2823	-1,0626	0,0229	0,3061	0,0001
173	BCP173	C121 - H126	0,2839	-1,0879	0,0081	0,3094	0,0001
174	BCP174	H127 - H131	0,0156	0,0617	0,4265	-0,0023	0,3165
175	BCP175	C122 - H127	0,2818	-1,0543	0,0254	0,3051	0,0001
176	BCP176	C75 - H135	0,0044	0,0130	1,7409	-0,0006	0,7322
177	BCP177	C75 - H139	0,0039	0,0116	1,1420	-0,0006	0,4875
178	BCP178	C123 - N128	0,3254	-0,8783	0,1124	0,5310	0,0001
179	BCP179	N128 - C136	0,2572	-0,6720	0,0241	0,3328	0,0002
180	BCP180	C129 - H132	0,2808	-1,0511	0,0298	0,3022	0,0007
181	BCP181	N128 - C129	0,2606	-0,7008	0,0243	0,3330	0,0001
182	BCP182	O40 - H133	0,0038	0,0146	0,5750	-0,0007	0,0536
183	BCP183	O79 - H135	0,0055	0,0221	0,0224	-0,0010	0,1457
184	BCP184	C129 - C130	0,2491	-0,5794	0,0329	0,2014	0,0004
185	BCP185	C129 - H131	0,2812	-1,0487	0,0312	0,3025	0,0007
186	BCP186	C130 - H134	0,2738	-0,9969	0,0041	0,2915	0,0002
187	BCP187	C130 - H133	0,2727	-0,9807	0,0058	0,2885	0,0003
188	BCP188	C130 - H135	0,2729	-0,9746	0,0056	0,2887	0,0002
189	BCP189	O70 - H141	0,0053	0,0215	0,2662	-0,0009	0,0746
190	BCP190	H125 - H138	0,0155	0,0606	0,4764	-0,0022	0,3078
191	BCP191	C136 - C137	0,2491	-0,5785	0,0295	0,2021	0,0007
192	BCP192	O70 - H138	0,0062	0,0273	1,0109	-0,0012	0,1496
193	BCP193	C136 - H139	0,2797	-1,0412	0,0341	0,3000	0,0008
194	BCP194	C136 - H138	0,2845	-1,0775	0,0321	0,3094	0,0007
195	BCP195	C137 - H140	0,2728	-0,9792	0,0060	0,2888	0,0003
196	BCP196	C137 - H141	0,2751	-1,0169	0,0029	0,2947	0,0002
197	BCP197	C137 - H142	0,2728	-0,9795	0,0065	0,2888	0,0003

GO1 + BR1

1	BCP1	C1 - C2	0,2773	-0,7071	0,0597	0,2413	0,0005
2	BCP2	C2 - C3	0,3307	-0,8646	0,2563	0,3365	0,0001
3	BCP3	C3 - C4	0,2840	-0,6937	0,1100	0,2508	0,0003
4	BCP4	C10 - C108	0,0061	0,0168	0,3163	-0,0007	0,0100
5	BCP5	C1 - C5	0,2613	-0,6222	0,0542	0,2163	0,0007
6	BCP6	C1 - H35	0,2638	-0,9330	0,0348	0,2726	0,0002
7	BCP7	C4 - C6	0,2885	-0,7114	0,1224	0,2585	0,0000
8	BCP8	C5 - C6	0,3091	-0,7625	0,2260	0,2945	0,0001
9	BCP9	C3 - C7	0,2719	-0,6669	0,0969	0,2319	0,0004
10	BCP10	C7 - C8	0,2514	-0,5853	0,0519	0,2040	0,0006
11	BCP11	C9 - C10	0,3022	-0,7519	0,1815	0,2815	0,0001
12	BCP12	C4 - C10	0,2959	-0,7309	0,1565	0,2701	0,0003
13	BCP13	C8 - C9	0,2571	-0,5977	0,0351	0,2112	0,0007
14	BCP14	C7 - O37	0,4135	0,4820	0,0612	0,7230	0,0000
15	BCP15	C15 - O111	0,0054	0,0181	0,6931	-0,0007	0,0337
16	BCP16	C8 - C11	0,2619	-0,6235	0,0558	0,2170	0,0006
17	BCP17	C8 - H12	0,2657	-0,9416	0,0140	0,2786	0,0000
18	BCP18	C11 - C13	0,2458	-0,4778	0,2711	0,2073	0,0257

19	BCP19	C9 - C15	0,2972	-0,7349	0,1626	0,2728	0,0002
20	BCP20	C13 - C14	0,2737	-0,6689	0,0773	0,2329	0,0005
21	BCP21	C14 - C15	0,2996	-0,7436	0,1680	0,2764	0,0002
22	BCP22	C5 - C16	0,2849	-0,7080	0,1377	0,2525	0,0001
23	BCP23	C16 - C17	0,2901	-0,7402	0,1337	0,2609	0,0001
24	BCP24	C6 - C19	0,2837	-0,6863	0,1361	0,2500	0,0001
25	BCP25	C17 - C18	0,3237	-0,8302	0,2647	0,3243	0,0001
26	BCP26	C19 - C20	0,3046	-0,7543	0,2116	0,2861	0,0001
27	BCP27	C18 - C19	0,2863	-0,6933	0,1441	0,2543	0,0003
28	BCP28	C10 - C21	0,2837	-0,6853	0,1373	0,2499	0,0001
29	BCP29	C22 - C104	0,0036	0,0094	1,9620	-0,0003	0,2677
30	BCP30	C21 - C22	0,3111	-0,7773	0,2361	0,2979	0,0000
31	BCP31	C20 - C21	0,2854	-0,6953	0,1268	0,2524	0,0000
32	BCP32	C15 - C23	0,2876	-0,6989	0,1489	0,2566	0,0000
33	BCP33	C22 - C23	0,2997	-0,7418	0,1818	0,2770	0,0001
34	BCP34	C23 - C24	0,2944	-0,7192	0,1757	0,2677	0,0001
35	BCP35	C14 - C26	0,3011	-0,7497	0,1714	0,2793	0,0000
36	BCP36	C24 - C25	0,3052	-0,7576	0,2092	0,2868	0,0001
37	BCP37	C25 - C26	0,2879	-0,7029	0,1392	0,2567	0,0000
38	BCP38	C18 - C27	0,2710	-0,6754	0,0828	0,2291	0,0002
39	BCP39	C20 - C29	0,2854	-0,6906	0,1434	0,2536	0,0001
40	BCP40	C27 - O56	0,2736	-0,4585	0,4889	0,3830	0,0050
41	BCP41	C27 - C28	0,2607	-0,5515	0,2380	0,2265	0,0341
42	BCP42	C28 - O56	0,2443	-0,2867	0,7879	0,2971	0,0038
43	BCP43	C28 - C29	0,2718	-0,6554	0,0756	0,2305	0,0008
44	BCP44	C22 - C31	0,2605	-0,6071	0,0571	0,2157	0,0006
45	BCP45	C29 - C30	0,3279	-0,8626	0,3510	0,3359	0,0002
46	BCP46	C30 - O43	0,3175	-0,2300	0,0184	0,5180	0,0010
47	BCP47	C30 - C31	0,2653	-0,6377	0,0803	0,2226	0,0016
48	BCP48	C32 - C33	0,2805	-0,6932	0,1299	0,2452	0,0001
49	BCP49	C24 - C33	0,2772	-0,6603	0,1238	0,2398	0,0002
50	BCP50	C31 - C32	0,2518	-0,5822	0,0697	0,2046	0,0008
51	BCP51	O40 - H44	0,0573	0,1750	0,0121	0,0048	0,0061
52	BCP52	C32 - O40	0,3972	0,3087	0,0454	0,6905	0,0001
53	BCP53	O40 - O79	0,0176	0,0746	0,0905	-0,0019	0,0116
54	BCP54	C1 - O34	0,2772	-0,6335	0,0429	0,3967	0,0006
55	BCP55	H36 - O38	0,0376	0,1223	0,0137	-0,0002	0,0167
56	BCP56	C5 - H119	0,0077	0,0253	0,2376	-0,0010	0,0926
57	BCP57	C16 - O38	0,3830	0,0637	0,0514	0,6650	0,0000
58	BCP58	O34 - H36	0,3358	-2,3866	0,0202	0,6631	0,0004
59	BCP59	C17 - H39	0,2834	-1,1070	0,0118	0,3111	0,0000
60	BCP60	C11 - O41	0,2714	-0,3596	0,3888	0,3953	0,0048
61	BCP61	C13 - O41	0,2367	-0,2477	0,8232	0,2649	0,0044
62	BCP62	C13 - C51	0,2802	-0,6980	0,0883	0,2454	0,0003
63	BCP63	C11 - H42	0,2864	-1,1327	0,0305	0,3151	0,0002
64	BCP64	O43 - H44	0,3169	-2,1921	0,0136	0,6171	0,0007
65	BCP65	C26 - C45	0,2969	-0,7285	0,1788	0,2719	0,0000
66	BCP66	C45 - C46	0,2881	-0,7012	0,1412	0,2569	0,0000
67	BCP67	C25 - C48	0,2915	-0,7145	0,1542	0,2626	0,0001
68	BCP68	C47 - C48	0,3140	-0,7854	0,2489	0,3031	0,0001
69	BCP69	C46 - C47	0,2863	-0,6997	0,1226	0,2542	0,0002
70	BCP70	C33 - C49	0,3187	-0,8232	0,3118	0,3164	0,0006
71	BCP71	C48 - C50	0,2742	-0,6596	0,0951	0,2356	0,0002

72	BCP72	C49 - C50	0,2794	-0,6936	0,1132	0,2435	0,0007
73	BCP73	C45 - C52	0,2840	-0,6900	0,1224	0,2507	0,0001
74	BCP74	C51 - C52	0,3310	-0,8531	0,3212	0,3390	0,0002
75	BCP75	C51 - H54	0,2817	-1,0836	0,0203	0,3073	0,0003
76	BCP76	C2 - H53	0,2848	-1,1437	0,0065	0,3161	0,0001
77	BCP77	C14 - C90	0,0067	0,0178	3,1357	-0,0007	0,8908
78	BCP78	C31 - H55	0,2633	-0,9610	0,0101	0,2812	0,0000
79	BCP79	C28 - H57	0,2847	-1,0988	0,0416	0,3099	0,0003
80	BCP80	C27 - O58	0,2935	-0,5140	0,1374	0,4559	0,0018
81	BCP81	O58 - H59	0,3532	-2,3881	0,0208	0,6642	0,0008
82	BCP82	C52 - C60	0,2640	-0,6433	0,0743	0,2202	0,0008
83	BCP83	C62 - C65	0,2564	-0,5981	0,0419	0,2094	0,0006
84	BCP84	C46 - C62	0,3020	-0,7318	0,2406	0,2845	0,0002
85	BCP85	C60 - O72	0,2737	-0,5005	0,0438	0,4042	0,0005
86	BCP86	C60 - C61	0,2619	-0,6382	0,0725	0,2173	0,0015
87	BCP87	O66 - H69	0,0184	0,0571	0,0679	0,0004	0,0704
88	BCP88	C61 - C62	0,2970	-0,7420	0,1945	0,2735	0,0001
89	BCP89	C63 - C64	0,2521	-0,5082	0,2408	0,2161	0,0297
90	BCP90	C47 - C63	0,2718	-0,6599	0,0565	0,2301	0,0003
91	BCP91	C65 - O67	0,2715	-0,5691	0,0327	0,3913	0,0007
92	BCP92	C64 - H71	0,2860	-1,1391	0,0291	0,3153	0,0002
93	BCP93	C64 - C65	0,2644	-0,6568	0,0824	0,2203	0,0009
94	BCP94	C61 - O66	0,3857	0,1958	0,0256	0,6674	0,0000
95	BCP95	O67 - H69	0,3538	-2,4013	0,0225	0,6688	0,0005
96	BCP96	C65 - H68	0,2752	-0,9989	0,0351	0,2891	0,0001
97	BCP97	C63 - O70	0,2421	-0,2733	0,8013	0,2799	0,0027
98	BCP98	C64 - O70	0,2617	-0,2915	0,4502	0,3734	0,0045
99	BCP99	O66 - H73	0,0243	0,0874	0,7365	0,0000	0,2766
100	BCP100	O72 - H73	0,3458	-2,3401	0,0214	0,6520	0,0003
101	BCP101	C60 - H74	0,2705	-0,9710	0,0494	0,2833	0,0001
102	BCP102	C75 - H78	0,2774	-1,0197	0,0126	0,2961	0,0003
103	BCP103	C50 - C75	0,3238	-0,8235	0,2798	0,3241	0,0000
104	BCP104	C76 - O77	0,4084	0,4315	0,0717	0,7125	0,0000
105	BCP105	C63 - C76	0,2652	-0,6524	0,0603	0,2210	0,0004
106	BCP106	C75 - C76	0,2813	-0,7050	0,1110	0,2473	0,0002
107	BCP107	H78 - H80	0,0219	0,0690	0,4302	-0,0004	0,2988
108	BCP108	C49 - O79	0,3118	-0,1645	0,0104	0,5038	0,0013
109	BCP109	O79 - H80	0,3566	-2,4333	0,0148	0,6763	0,0009
110	BCP110	C52 - N98	0,0073	0,0255	1,4662	-0,0011	0,2768
111	BCP111	O66 - H83	0,0057	0,0219	0,3964	-0,0009	0,0590
112	BCP112	C61 - N98	0,0064	0,0197	0,4774	-0,0007	0,0880
113	BCP113	O72 - H99	0,0061	0,0235	0,2697	-0,0007	0,0497
114	BCP114	O72 - H83	0,0076	0,0284	0,6989	-0,0010	0,0288
115	BCP115	O66 - H101	0,0074	0,0271	0,3838	-0,0009	0,0823
116	BCP116	C82 - N98	0,2531	-0,6458	0,0338	0,3299	0,0004
117	BCP117	C81 - C82	0,2511	-0,5896	0,0252	0,2051	0,0008
118	BCP118	C81 - H83	0,2756	-1,0071	0,0086	0,2946	0,0002
119	BCP119	C81 - H84	0,2745	-1,0043	0,0072	0,2930	0,0002
120	BCP120	C81 - H85	0,2733	-0,9827	0,0084	0,2894	0,0003
121	BCP121	C62 - H93	0,0055	0,0156	0,2824	-0,0005	0,0203
122	BCP122	O70 - H93	0,0099	0,0351	0,2532	-0,0009	0,0701
123	BCP123	C86 - C91	0,2828	-0,6917	0,1448	0,2506	0,0008
124	BCP124	C86 - C87	0,3203	-0,8134	0,2855	0,3164	0,0003

125	BCP125	C87 - C88	0,2931	-0,7256	0,1542	0,2668	0,0002
126	BCP126	C88 - C89	0,2982	-0,7567	0,1871	0,2768	0,0017
127	BCP127	O41 - H97	0,0101	0,0393	0,1390	-0,0010	0,0433
128	BCP128	C90 - C91	0,2997	-0,7482	0,2151	0,2790	0,0002
129	BCP129	C91 - N98	0,3268	-0,9175	0,1009	0,5344	0,0006
130	BCP130	C89 - C90	0,3194	-0,8386	0,3109	0,3209	0,0008
131	BCP131	C82 - H100	0,2817	-1,0509	0,0337	0,3032	0,0004
132	BCP132	H95 - H100	0,0106	0,0448	0,9343	-0,0024	0,4840
133	BCP133	C92 - H95	0,2722	-0,9737	0,0122	0,2880	0,0003
134	BCP134	C86 - C92	0,2539	-0,5916	0,0299	0,2089	0,0003
135	BCP135	C92 - H93	0,2774	-1,0288	0,0096	0,2990	0,0003
136	BCP136	C92 - H94	0,2765	-1,0161	0,0092	0,2962	0,0005
137	BCP137	C87 - H96	0,2822	-1,0744	0,0129	0,3061	0,0000
138	BCP138	C90 - H97	0,2804	-1,0642	0,0281	0,3044	0,0001
139	BCP139	N98 - H99	0,3355	-1,6102	0,0416	0,4485	0,0002
140	BCP140	H93 - H101	0,0102	0,0446	1,2099	-0,0025	0,5796
141	BCP141	C82 - H101	0,2814	-1,0614	0,0348	0,3033	0,0003
142	BCP142	C88 - C102	0,3019	-0,7472	0,1947	0,2818	0,0001
143	BCP143	H55 - C105	0,0052	0,0163	0,2536	-0,0008	0,0345
144	BCP144	C102 - C10[illegible]	0,2969	-0,7282	0,1820	0,2732	0,0003
145	BCP145	O41 - O111	0,0086	0,0328	0,0584	-0,0006	0,0145
146	BCP146	C89 - O111	0,2873	-0,0135	0,0115	0,4459	0,0027
147	BCP147	C103 - C10[illegible]	0,2978	-0,7491	0,2028	0,2764	0,0017
148	BCP148	C20 - C107	0,0046	0,0125	0,4071	-0,0005	0,4381
149	BCP149	C29 - H123	0,0047	0,0166	1,8874	-0,0008	0,0424
150	BCP150	C103 - C10[illegible]	0,2916	-0,7164	0,1613	0,2639	0,0002
151	BCP151	C18 - H123	0,0052	0,0169	0,7112	-0,0008	0,0140
152	BCP152	C105 - C10[illegible]	0,3195	-0,8063	0,2969	0,3153	0,0001
153	BCP153	C6 - N112	0,0064	0,0189	1,6796	-0,0005	0,0252
154	BCP154	O38 - H116	0,0073	0,0248	1,4209	-0,0008	0,1749
155	BCP155	C106 - C10[illegible]	0,2851	-0,7017	0,1455	0,2538	0,0005
156	BCP156	C104 - O11[illegible]	0,2887	-0,0245	0,0124	0,4492	0,0028
157	BCP157	C104 - C10[illegible]	0,3166	-0,8272	0,2990	0,3148	0,0006
158	BCP158	C107 - C10[illegible]	0,3023	-0,7572	0,2262	0,2841	0,0003
159	BCP159	C105 - H10[illegible]	0,2808	-1,0600	0,0148	0,3034	0,0000
160	BCP160	C2 - H119	0,0057	0,0183	1,2191	-0,0008	0,2964
161	BCP161	C108 - H11[illegible]	0,2799	-1,0516	0,0306	0,3028	0,0000
162	BCP162	C107 - N11[illegible]	0,3298	-0,8853	0,1022	0,5455	0,0001
163	BCP163	O34 - C114	0,0059	0,0268	3,5717	-0,0016	0,1113
164	BCP164	O34 - H119	0,0063	0,0243	0,6882	-0,0009	0,0386
165	BCP165	C114 - H11[illegible]	0,2730	-0,9799	0,0105	0,2890	0,0004
166	BCP166	N112 - C11[illegible]	0,2585	-0,6653	0,0404	0,3471	0,0001
167	BCP167	C113 - C11[illegible]	0,2512	-0,5901	0,0204	0,2055	0,0007
168	BCP168	C114 - H11[illegible]	0,2747	-1,0022	0,0126	0,2930	0,0004
169	BCP169	C113 - H11[illegible]	0,2784	-1,0307	0,0365	0,2973	0,0003
170	BCP170	C114 - H11[illegible]	0,2758	-1,0201	0,0074	0,2959	0,0002
171	BCP171	C113 - H11[illegible]	0,2782	-1,0260	0,0343	0,2966	0,0004
172	BCP172	N112 - H12[illegible]	0,3375	-1,6154	0,0440	0,4511	0,0001
173	BCP173	C27 - H123	0,0050	0,0182	4,0604	-0,0009	0,4193
174	BCP174	C121 - H12[illegible]	0,2703	-0,9645	0,0100	0,2848	0,0002
175	BCP175	C106 - C12[illegible]	0,2563	-0,5992	0,0350	0,2111	0,0001
176	BCP176	C121 - H12[illegible]	0,2753	-1,0079	0,0062	0,2941	0,0003
177	BCP177	C121 - H12[illegible]	0,2713	-0,9734	0,0104	0,2866	0,0002

178	BCP178	C102 - O135	0,0137	0,0470	0,7260	-0,0012	0,0548
179	BCP179	C102 - C125	0,2595	-0,6168	0,0276	0,2137	0,0021
180	BCP180	C125 - C126	0,3033	-0,7490	0,2216	0,2861	0,0001
181	BCP181	C126 - C127	0,3108	-0,7959	0,2046	0,2988	0,0000
182	BCP182	C127 - C128	0,3107	-0,7977	0,1949	0,2982	0,0000
183	BCP183	C127 - H131	0,2822	-1,0804	0,0065	0,3062	0,0000
184	BCP184	C125 - C130	0,2991	-0,7259	0,2167	0,2769	0,0008
185	BCP185	C128 - C129	0,3129	-0,8048	0,2043	0,3025	0,0000
186	BCP186	C128 - H132	0,2822	-1,0794	0,0098	0,3062	0,0000
187	BCP187	C129 - C130	0,3051	-0,7626	0,1949	0,2875	0,0000
188	BCP188	C129 - H133	0,2847	-1,1103	0,0085	0,3126	0,0000
189	BCP189	C130 - C136	0,2735	-0,6899	0,1258	0,2363	0,0002
190	BCP190	C126 - H134	0,2820	-1,0722	0,0147	0,3056	0,0000
191	BCP191	H55 - O135	0,0227	0,0788	0,1063	-0,0012	0,0130
192	BCP192	O43 - H143	0,0051	0,0229	2,4271	-0,0012	0,2137
193	BCP193	O40 - H143	0,0067	0,0275	0,9875	-0,0012	0,1569
194	BCP194	O79 - H144	0,0075	0,0235	0,0548	-0,0003	0,0008
195	BCP195	C136 - O137	0,3183	-0,2666	0,0070	0,5203	0,0023
196	BCP196	O135 - C136	0,4085	0,2271	0,0845	0,7213	0,0003
197	BCP197	O137 - C139	0,2307	-0,1892	0,0210	0,3173	0,0016
198	BCP198	C138 - C139	0,2586	-0,6274	0,0336	0,2156	0,0007
199	BCP199	C138 - H140	0,2743	-0,9974	0,0069	0,2919	0,0003
200	BCP200	C138 - H141	0,2740	-0,9946	0,0070	0,2914	0,0003
201	BCP201	C138 - H142	0,2736	-0,9947	0,0050	0,2913	0,0002
202	BCP202	O40 - H144	0,0066	0,0272	1,1254	-0,0012	0,1973
203	BCP203	C139 - H143	0,2825	-1,0708	0,0455	0,3049	0,0005
204	BCP204	C139 - H144	0,2852	-1,1083	0,0409	0,3118	0,0006

GO1 + BY2

1	BCP1	C1 - C2	0,2748	-0,6953	0,0638	0,2373	0,0004
2	BCP2	C3 - C4	0,2846	-0,6934	0,1155	0,2518	0,0006
3	BCP3	C2 - H53	0,2833	-1,1178	0,0038	0,3115	0,0001
4	BCP4	C2 - C3	0,3305	-0,8601	0,2731	0,3368	0,0004
5	BCP5	C1 - C5	0,2605	-0,6189	0,0530	0,2151	0,0005
6	BCP6	C4 - C6	0,2917	-0,7242	0,1267	0,2636	0,0001
7	BCP7	C4 - C10	0,2953	-0,7290	0,1540	0,2689	0,0001
8	BCP8	C5 - C16	0,2864	-0,7133	0,1431	0,2549	0,0001
9	BCP9	C5 - C6	0,3092	-0,7648	0,2241	0,2948	0,0000
10	BCP10	C3 - C7	0,2744	-0,6822	0,0892	0,2363	0,0002
11	BCP11	C7 - C8	0,2515	-0,5859	0,0462	0,2046	0,0002
12	BCP12	C9 - C10	0,3020	-0,7506	0,1848	0,2814	0,0001
13	BCP13	C8 - C9	0,2538	-0,5825	0,0419	0,2069	0,0004
14	BCP14	C8 - C11	0,2646	-0,6366	0,0525	0,2202	0,0007
15	BCP15	C9 - C15	0,2976	-0,7356	0,1666	0,2736	0,0003
16	BCP16	C8 - H12	0,2670	-0,9512	0,0152	0,2803	0,0001
17	BCP17	C11 - C13	0,2454	-0,4793	0,2595	0,2065	0,0272
18	BCP18	C13 - C14	0,2717	-0,6619	0,0705	0,2299	0,0006
19	BCP19	C14 - C15	0,2990	-0,7411	0,1686	0,2754	0,0002
20	BCP20	H36 - O38	0,0365	0,1178	0,0141	-0,0001	0,0190
21	BCP21	C16 - O38	0,3823	0,0629	0,0506	0,6632	0,0000
22	BCP22	C6 - C19	0,2840	-0,6889	0,1332	0,2505	0,0001

23	BCP23	C16 - C17	0,2899	-0,7399	0,1322	0,2604	0,0000
24	BCP24	C18 - C19	0,2863	-0,6935	0,1442	0,2542	0,0003
25	BCP25	C17 - H39	0,2828	-1,0979	0,0122	0,3094	0,0000
26	BCP26	C17 - C18	0,3240	-0,8308	0,2675	0,3247	0,0001
27	BCP27	C19 - C20	0,3034	-0,7497	0,2083	0,2839	0,0000
28	BCP28	C10 - C21	0,2841	-0,6867	0,1386	0,2507	0,0000
29	BCP29	C20 - C21	0,2851	-0,6923	0,1305	0,2519	0,0001
30	BCP30	C15 - C23	0,2860	-0,6927	0,1461	0,2540	0,0000
31	BCP31	C21 - C22	0,3084	-0,7651	0,2338	0,2930	0,0001
32	BCP32	C22 - C23	0,3006	-0,7405	0,1983	0,2787	0,0001
33	BCP33	C23 - C24	0,2924	-0,7117	0,1721	0,2644	0,0002
34	BCP34	C14 - C26	0,3013	-0,7504	0,1733	0,2797	0,0000
35	BCP35	C25 - C26	0,2864	-0,6972	0,1368	0,2542	0,0000
36	BCP36	C24 - C33	0,2752	-0,6511	0,1257	0,2368	0,0001
37	BCP37	C24 - C25	0,3068	-0,7607	0,2230	0,2899	0,0001
38	BCP38	C18 - C27	0,2713	-0,6782	0,0764	0,2295	0,0002
39	BCP39	C27 - O58	0,2913	-0,5123	0,1353	0,4504	0,0018
40	BCP40	C20 - C29	0,2852	-0,6899	0,1434	0,2532	0,0001
41	BCP41	C27 - C28	0,2627	-0,5621	0,2310	0,2291	0,0347
42	BCP42	C28 - H57	0,2848	-1,1011	0,0408	0,3103	0,0003
43	BCP43	C28 - C29	0,2708	-0,6521	0,0671	0,2290	0,0007
44	BCP44	C29 - C30	0,3276	-0,8595	0,3579	0,3358	0,0001
45	BCP45	C22 - C31	0,2570	-0,5960	0,0459	0,2106	0,0007
46	BCP46	C32 - C33	0,2798	-0,6852	0,1433	0,2444	0,0002
47	BCP47	C22 - H106	0,0065	0,0217	4,2037	-0,0010	0,1771
48	BCP48	C30 - C31	0,2604	-0,6201	0,0696	0,2153	0,0012
49	BCP49	C33 - C49	0,3157	-0,8115	0,3015	0,3106	0,0006
50	BCP50	C31 - C32	0,2489	-0,5747	0,0554	0,2006	0,0007
51	BCP51	O40 - H44	0,0557	0,1744	0,0140	0,0038	0,0070
52	BCP52	C23 - C83	0,0082	0,0242	3,8831	-0,0011	0,1702
53	BCP53	C32 - O40	0,3995	0,3212	0,0545	0,6958	0,0001
54	BCP54	O40 - O79	0,0189	0,0808	0,0945	-0,0022	0,0108
55	BCP55	C1 - O34	0,2719	-0,6081	0,0414	0,3839	0,0005
56	BCP56	C1 - H35	0,2668	-0,9561	0,0335	0,2778	0,0002
57	BCP57	O34 - H36	0,3355	-2,3677	0,0201	0,6581	0,0003
58	BCP58	C7 - O37	0,4108	0,4873	0,0508	0,7157	0,0000
59	BCP59	C11 - O41	0,2697	-0,3314	0,3936	0,3935	0,0046
60	BCP60	C13 - O41	0,2351	-0,2350	0,8514	0,2663	0,0047
61	BCP61	C11 - H42	0,2858	-1,1194	0,0322	0,3127	0,0002
62	BCP62	C30 - O43	0,3202	-0,2177	0,0190	0,5241	0,0012
63	BCP63	O43 - H44	0,3196	-2,2603	0,0130	0,6330	0,0007
64	BCP64	C52 - C60	0,2627	-0,6360	0,0828	0,2187	0,0010
65	BCP65	C45 - C46	0,2872	-0,6995	0,1340	0,2554	0,0001
66	BCP66	C26 - C45	0,2976	-0,7314	0,1772	0,2728	0,0000
67	BCP67	C47 - C48	0,3155	-0,7901	0,2572	0,3059	0,0001
68	BCP68	C25 - C48	0,2891	-0,7049	0,1521	0,2589	0,0002
69	BCP69	C48 - C50	0,2742	-0,6588	0,0970	0,2356	0,0002
70	BCP70	C46 - C47	0,2857	-0,6985	0,1201	0,2532	0,0002
71	BCP71	C49 - O79	0,3175	-0,1471	0,0105	0,5168	0,0014
72	BCP72	C50 - C75	0,3239	-0,8237	0,2786	0,3241	0,0000
73	BCP73	C49 - C50	0,2788	-0,6916	0,1103	0,2426	0,0007
74	BCP74	H78 - H80	0,0218	0,0682	0,4211	-0,0004	0,2912
75	BCP75	C51 - H54	0,2822	-1,0923	0,0181	0,3086	0,0004

76	BCP76	C13 - C51	0,2803	-0,6997	0,0847	0,2458	0,0003
77	BCP77	C45 - C52	0,2841	-0,6915	0,1155	0,2508	0,0001
78	BCP78	C51 - C52	0,3326	-0,8624	0,3168	0,3420	0,0002
79	BCP79	C31 - H55	0,2610	-0,8948	0,0107	0,2697	0,0000
80	BCP80	C27 - O56	0,2742	-0,4610	0,4893	0,3847	0,0051
81	BCP81	C28 - O56	0,2430	-0,2778	0,8210	0,2920	0,0040
82	BCP82	O58 - H59	0,3530	-2,3860	0,0208	0,6636	0,0007
83	BCP83	C60 - O72	0,2822	-0,5285	0,0360	0,4252	0,0005
84	BCP84	C62 - C65	0,2576	-0,6044	0,0366	0,2112	0,0007
85	BCP85	C46 - C62	0,3052	-0,7434	0,2442	0,2896	0,0001
86	BCP86	C60 - C61	0,2621	-0,6373	0,0777	0,2177	0,0016
87	BCP87	C61 - C62	0,2918	-0,7263	0,1643	0,2640	0,0001
88	BCP88	O66 - H69	0,0160	0,0514	0,1241	0,0002	0,1241
89	BCP89	C63 - C64	0,2529	-0,5138	0,2300	0,2173	0,0297
90	BCP90	C47 - C63	0,2721	-0,6609	0,0591	0,2307	0,0003
91	BCP91	C63 - C76	0,2655	-0,6536	0,0606	0,2213	0,0003
92	BCP92	C64 - C65	0,2652	-0,6603	0,0841	0,2215	0,0010
93	BCP93	C64 - H71	0,2863	-1,1454	0,0290	0,3164	0,0002
94	BCP94	C26 - C82	0,0053	0,0162	0,9914	-0,0007	0,1345
95	BCP95	C65 - O67	0,2705	-0,5656	0,0334	0,3883	0,0007
96	BCP96	C61 - O66	0,3922	0,2700	0,0304	0,6801	0,0000
97	BCP97	O67 - H69	0,3544	-2,4066	0,0226	0,6699	0,0005
98	BCP98	C65 - H68	0,2781	-1,0297	0,0333	0,2956	0,0001
99	BCP99	C64 - O70	0,2596	-0,2787	0,4652	0,3686	0,0046
100	BCP100	C63 - O70	0,2397	-0,2570	0,8483	0,2730	0,0032
101	BCP101	O66 - H73	0,0246	0,0876	0,6964	-0,0001	0,1599
102	BCP102	O72 - H73	0,3454	-2,3461	0,0226	0,6535	0,0004
103	BCP103	C60 - H74	0,2683	-0,9506	0,0531	0,2790	0,0001
104	BCP104	C75 - C76	0,2812	-0,7051	0,1096	0,2472	0,0003
105	BCP105	C76 - O77	0,4091	0,4365	0,0741	0,7139	0,0000
106	BCP106	C75 - H78	0,2779	-1,0260	0,0112	0,2972	0,0003
107	BCP107	O79 - H80	0,3560	-2,4451	0,0139	0,6785	0,0009
108	BCP108	C25 - N96	0,0055	0,0163	2,1351	-0,0004	0,0115
109	BCP109	C46 - H98	0,0052	0,0178	0,4179	-0,0010	0,2400
110	BCP110	C81 - C82	0,2951	-0,7386	0,1774	0,2715	0,0007
111	BCP111	H55 - H103	0,0031	0,0130	1,2862	-0,0008	0,1383
112	BCP112	C82 - C83	0,2962	-0,7398	0,1906	0,2733	0,0007
113	BCP113	O41 - C85	0,0098	0,0355	6,1088	-0,0013	0,7182
114	BCP114	C83 - C84	0,3152	-0,7994	0,2631	0,3077	0,0003
115	BCP115	O41 - H112	0,0173	0,0637	0,2095	-0,0010	0,0555
116	BCP116	C84 - C85	0,2990	-0,7397	0,1932	0,2772	0,0003
117	BCP117	C81 - C86	0,3191	-0,8198	0,2594	0,3149	0,0003
118	BCP118	C85 - C86	0,2980	-0,7361	0,1805	0,2748	0,0004
119	BCP119	O37 - N111	0,0070	0,0234	0,2469	-0,0007	0,0261
120	BCP120	C85 - C87	0,2811	-0,6903	0,1484	0,2493	0,0001
121	BCP121	C3 - C89	0,0076	0,0254	0,7247	-0,0014	0,2158
122	BCP122	O37 - H108	0,0057	0,0210	0,4540	-0,0007	0,0596
123	BCP123	C87 - C88	0,2856	-0,7032	0,1673	0,2573	0,0002
124	BCP124	O34 - H109	0,0104	0,0311	0,0162	-0,0002	0,0122
125	BCP125	O34 - H117	0,0089	0,0269	0,2431	-0,0003	0,0105
126	BCP126	C5 - C90	0,0065	0,0171	2,2867	-0,0007	0,5539
127	BCP127	C88 - C89	0,2949	-0,7213	0,1810	0,2698	0,0003
128	BCP128	O38 - H117	0,0072	0,0300	1,0887	-0,0016	0,7727

129	BCP129	C89 - C90	0,3196	-0,8188	0,2679	0,3167	0,0005
130	BCP130	C17 - N113	0,0057	0,0156	1,1468	-0,0004	0,1260
131	BCP131	C90 - C91	0,2940	-0,7362	0,1669	0,2698	0,0006
132	BCP132	C88 - C92	0,2965	-0,7319	0,1791	0,2723	0,0003
133	BCP133	C91 - C93	0,2938	-0,7331	0,1752	0,2690	0,0007
134	BCP134	C92 - C93	0,3181	-0,8134	0,2653	0,3133	0,0004
135	BCP135	O70 - H99	0,0124	0,0354	0,0197	0,0001	0,0088
136	BCP136	C82 - N96	0,3230	-0,9153	0,1113	0,5198	0,0004
137	BCP137	C94 - H105	0,0142	0,0687	1,0831	-0,0037	0,9345
138	BCP138	C48 - H101	0,0075	0,0280	0,2458	-0,0014	0,1254
139	BCP139	O70 - H100	0,0054	0,0213	0,2683	-0,0007	0,0253
140	BCP140	C94 - N96	0,2625	-0,7044	0,0262	0,3456	0,0002
141	BCP141	C95 - N96	0,2659	-0,7368	0,0381	0,3456	0,0001
142	BCP142	C94 - H97	0,2746	-1,0067	0,0385	0,2914	0,0006
143	BCP143	C94 - H98	0,2789	-1,0401	0,0358	0,2997	0,0007
144	BCP144	C94 - H99	0,2830	-1,1024	0,0288	0,3112	0,0007
145	BCP145	C95 - H100	0,2810	-1,0715	0,0296	0,3053	0,0005
146	BCP146	C95 - H102	0,2750	-1,0138	0,0353	0,2926	0,0005
147	BCP147	H101 - H103	0,0141	0,0563	0,6150	-0,0023	0,3120
148	BCP148	C95 - H101	0,2785	-1,0295	0,0346	0,2984	0,0005
149	BCP149	C83 - H103	0,2809	-1,0415	0,0256	0,3031	0,0001
150	BCP150	C86 - H104	0,2824	-1,0694	0,0078	0,3058	0,0001
151	BCP151	C81 - H105	0,2831	-1,0723	0,0222	0,3080	0,0001
152	BCP152	C84 - H106	0,2837	-1,0828	0,0082	0,3092	0,0000
153	BCP153	H106 - H107	0,0139	0,0610	0,6664	-0,0031	0,5642
154	BCP154	C92 - H107	0,2841	-1,0871	0,0074	0,3096	0,0001
155	BCP155	C89 - H108	0,2816	-1,0548	0,0099	0,3038	0,0002
156	BCP156	C90 - H109	0,2838	-1,0979	0,0204	0,3114	0,0001
157	BCP157	C93 - H110	0,2822	-1,0602	0,0244	0,3059	0,0001
158	BCP158	C87 - N111	0,3365	-0,8182	0,0607	0,5710	0,0002
159	BCP159	H108 - H122	0,0156	0,0606	0,4852	-0,0019	0,4702
160	BCP160	N111 - H112	0,3324	-1,6607	0,0345	0,4573	0,0001
161	BCP161	C91 - N113	0,3262	-0,8892	0,1160	0,5327	0,0002
162	BCP162	C114 - H115	0,2752	-1,0150	0,0382	0,2929	0,0006
163	BCP163	H109 - H117	0,0157	0,0587	0,5087	-0,0018	0,2549
164	BCP164	N113 - C114	0,2611	-0,6918	0,0317	0,3459	0,0001
165	BCP165	C114 - H116	0,2809	-1,0663	0,0340	0,3046	0,0008
166	BCP166	C114 - H117	0,2833	-1,0895	0,0320	0,3100	0,0006
167	BCP167	O58 - H120	0,0037	0,0163	0,2845	-0,0009	0,1207
168	BCP168	C18 - H119	0,0046	0,0169	1,2261	-0,0010	0,3037
169	BCP169	C118 - H121	0,2747	-1,0080	0,0364	0,2917	0,0005
170	BCP170	N113 - C118	0,2657	-0,7307	0,0329	0,3485	0,0002
171	BCP171	H110 - C118	0,0136	0,0707	2,7997	-0,0041	0,9320
172	BCP172	C118 - H119	0,2783	-1,0361	0,0336	0,2985	0,0006
173	BCP173	C118 - H120	0,2813	-1,0766	0,0294	0,3061	0,0007
174	BCP174	N111 - H122	0,3377	-1,6513	0,0354	0,4572	0,0001

GO2 + BB26

1	BCP1	C1 - C2	0,3154	-0,8140	0,2799	0,3085	0,0011
2	BCP2	C10 - O49	0,2344	-0,2259	0,8339	0,2632	0,0047
3	BCP3	C3 - C4	0,3052	-0,7608	0,2006	0,2869	0,0001
4	BCP4	C2 - H71	0,2774	-1,0236	0,0193	0,2968	0,0000

5	BCP5	C2 - C3	0,3022	-0,7523	0,1980	0,2821	0,0001
6	BCP6	C1 - C5	0,3056	-0,7775	0,2350	0,2890	0,0012
7	BCP7	C1 - O47	0,2832	-0,2259	0,0201	0,4375	0,0009
8	BCP8	C4 - C6	0,3114	-0,7850	0,2094	0,2982	0,0001
9	BCP9	C4 - C10	0,2712	-0,6615	0,0537	0,2301	0,0008
10	BCP10	C5 - C23	0,2582	-0,6149	0,0441	0,2119	0,0003
11	BCP11	C5 - C6	0,3098	-0,7687	0,2262	0,2956	0,0001
12	BCP12	C3 - C7	0,2847	-0,7065	0,0958	0,2519	0,0001
13	BCP13	C7 - C8	0,3253	-0,8414	0,2489	0,3261	0,0003
14	BCP14	C9 - C10	0,2718	-0,6665	0,0360	0,2304	0,0004
15	BCP15	C8 - C9	0,2894	-0,7135	0,1181	0,2598	0,0002
16	BCP16	C8 - C11	0,2676	-0,6485	0,0974	0,2258	0,0003
17	BCP17	C11 - C12	0,2690	-0,6495	0,1034	0,2277	0,0001
18	BCP18	C9 - C13	0,3252	-0,8309	0,2837	0,3255	0,0001
19	BCP19	C12 - C14	0,3213	-0,8070	0,2717	0,3191	0,0001
20	BCP20	C13 - C14	0,2809	-0,6743	0,1153	0,2459	0,0004
21	BCP21	C15 - O86	0,4083	0,4136	0,0740	0,7130	0,0000
22	BCP22	C12 - C15	0,2693	-0,6568	0,0900	0,2280	0,0003
23	BCP23	C19 - H85	0,2864	-1,1267	0,0334	0,3142	0,0002
24	BCP24	C15 - C16	0,2719	-0,6676	0,0917	0,2320	0,0004
25	BCP25	C14 - C18	0,2532	-0,6053	0,0154	0,2047	0,0007
26	BCP26	C16 - C19	0,2744	-0,6694	0,0741	0,2345	0,0008
27	BCP27	C16 - C17	0,3289	-0,8410	0,2918	0,3336	0,0001
28	BCP28	C17 - C22	0,2785	-0,6653	0,1178	0,2423	0,0009
29	BCP29	C17 - C18	0,2499	-0,5810	0,0123	0,2001	0,0004
30	BCP30	C20 - C21	0,2759	-0,6752	0,0809	0,2367	0,0006
31	BCP31	C19 - C20	0,2395	-0,4417	0,3493	0,1988	0,0246
32	BCP32	C21 - C22	0,3257	-0,8284	0,2858	0,3272	0,0001
33	BCP33	C18 - C32	0,2549	-0,5978	0,0072	0,2071	0,0005
34	BCP34	C23 - H54	0,2754	-1,0113	0,0368	0,2915	0,0003
35	BCP35	C23 - C24	0,2640	-0,6435	0,0584	0,2197	0,0008
36	BCP36	C6 - C26	0,2594	-0,6284	0,0112	0,2131	0,0009
37	BCP37	C24 - C25	0,3317	-0,8467	0,3319	0,3396	0,0001
38	BCP38	C26 - C27	0,2524	-0,5886	0,0141	0,2038	0,0015
39	BCP39	C25 - C26	0,2604	-0,6239	0,0098	0,2141	0,0002
40	BCP40	C10 - C28	0,2566	-0,5362	0,1861	0,2203	0,0308
41	BCP41	C27 - C28	0,2722	-0,6601	0,0557	0,2312	0,0004
42	BCP42	C28 - O49	0,2534	-0,3252	0,5295	0,3331	0,0040
43	BCP43	C25 - C36	0,2761	-0,6603	0,0987	0,2388	0,0005
44	BCP44	C13 - C30	0,2537	-0,5935	0,0377	0,2057	0,0008
45	BCP45	C28 - C29	0,2414	-0,5499	0,0603	0,1889	0,0002
46	BCP46	C29 - C30	0,2403	-0,5515	0,0744	0,1882	0,0001
47	BCP47	C30 - C31	0,2629	-0,6347	0,0353	0,2178	0,0001
48	BCP48	C22 - C34	0,2721	-0,6670	0,0709	0,2315	0,0005
49	BCP49	C31 - C32	0,3301	-0,8331	0,3337	0,3375	0,0002
50	BCP50	C33 - C34	0,2403	-0,4539	0,2659	0,1993	0,0254
51	BCP51	C34 - C35	0,2709	-0,6612	0,0730	0,2295	0,0005
52	BCP52	C32 - C33	0,2712	-0,6554	0,0822	0,2303	0,0004
53	BCP53	C35 - C82	0,3310	-0,8572	0,3001	0,3395	0,0001
54	BCP54	C35 - C46	0,2826	-0,7092	0,1329	0,2478	0,0007
55	BCP55	C36 - C61	0,2551	-0,6022	0,0658	0,2076	0,0008
56	BCP56	C27 - C38	0,3315	-0,8533	0,3035	0,3389	0,0000
57	BCP57	C36 - C37	0,3308	-0,8529	0,3141	0,3386	0,0002

58	BCP58	C38 - C39	0,2725	-0,6742	0,0865	0,2327	0,0003
59	BCP59	C37 - H75	0,2834	-1,1058	0,0161	0,3113	0,0001
60	BCP60	C37 - C38	0,2792	-0,6795	0,0923	0,2444	0,0001
61	BCP61	C39 - C40	0,2737	-0,6745	0,1149	0,2346	0,0009
62	BCP62	C29 - C40	0,2469	-0,5633	0,0504	0,1975	0,0019
63	BCP63	C31 - C42	0,2723	-0,6373	0,0992	0,2340	0,0030
64	BCP64	C29 - O50	0,2748	-0,5337	0,0302	0,4047	0,0011
65	BCP65	C40 - C41	0,3317	-0,8464	0,2079	0,3459	0,0037
66	BCP66	C41 - C42	0,3183	-0,8131	0,1706	0,3164	0,0050
67	BCP67	C44 - C45	0,2898	-0,7047	0,1616	0,2614	0,0002
68	BCP68	C46 - O79	0,3028	-0,2044	0,0058	0,4832	0,0010
69	BCP69	C33 - C44	0,2712	-0,6553	0,0806	0,2306	0,0002
70	BCP70	C43 - O58	0,3089	-0,2463	0,0138	0,4972	0,0010
71	BCP71	C42 - C43	0,2942	-0,7418	0,1762	0,2671	0,0010
72	BCP72	C43 - C44	0,3221	-0,8376	0,3105	0,3187	0,0012
73	BCP73	C45 - C46	0,3242	-0,8405	0,3412	0,3283	0,0006
74	BCP74	C45 - H78	0,2788	-1,0470	0,0224	0,3006	0,0000
75	BCP75	O79 - H80	0,3558	-2,4274	0,0160	0,6724	0,0008
76	BCP76	O47 - H48	0,3558	-2,4281	0,0189	0,6732	0,0007
77	BCP77	O49 - O50	0,0250	0,0943	0,5615	-0,0004	0,4148
78	BCP78	O50 - H51	0,3461	-2,3745	0,0203	0,6600	0,0007
79	BCP79	O50 - H113	0,0083	0,0263	0,0813	-0,0003	0,0131
80	BCP80	C11 - O52	0,4067	0,3643	0,0701	0,7109	0,0000
81	BCP81	O52 - O86	0,0154	0,0530	0,1247	-0,0003	0,0044
82	BCP82	O47 - H55	0,0205	0,0736	0,0278	-0,0005	0,0660
83	BCP83	C23 - O53	0,2621	-0,5246	0,0246	0,3678	0,0007
84	BCP84	O53 - H55	0,3563	-2,4241	0,0221	0,6751	0,0006
85	BCP85	C20 - O56	0,2584	-0,3712	0,5022	0,3449	0,0047
86	BCP86	C19 - O56	0,2627	-0,3804	0,4622	0,3605	0,0049
87	BCP87	C20 - H57	0,2865	-1,1323	0,0334	0,3150	0,0003
88	BCP88	C41 - H59	0,0225	0,0724	1,0952	-0,0004	0,1707
89	BCP89	O58 - H59	0,3397	-2,3024	0,0168	0,6400	0,0006
90	BCP90	C39 - O60	0,4023	0,3560	0,0509	0,7008	0,0000
91	BCP91	C24 - C63	0,2685	-0,6507	0,0667	0,2257	0,0007
92	BCP92	C61 - C62	0,2645	-0,6485	0,0064	0,2202	0,0009
93	BCP93	C62 - C63	0,2565	-0,5295	0,2288	0,2216	0,0311
94	BCP94	C63 - H65	0,2864	-1,1149	0,0334	0,3129	0,0002
95	BCP95	C62 - O64	0,2583	-0,3154	0,5586	0,3563	0,0037
96	BCP96	C63 - O64	0,2495	-0,3085	0,7169	0,3184	0,0034
97	BCP97	C62 - H66	0,2828	-1,0826	0,0351	0,3057	0,0002
98	BCP98	C61 - O67	0,2691	-0,5221	0,0187	0,3893	0,0009
99	BCP99	C61 - H68	0,2794	-1,0490	0,0368	0,2995	0,0001
100	BCP100	O67 - H69	0,3537	-2,3054	0,0226	0,6485	0,0006
101	BCP101	C33 - O70	0,2457	-0,3158	0,5817	0,3028	0,0042
102	BCP102	C34 - O70	0,2510	-0,3367	0,5160	0,3228	0,0043
103	BCP103	C7 - H72	0,2835	-1,1152	0,0015	0,3114	0,0003
104	BCP104	C26 - O73	0,2515	-0,5187	0,0250	0,3240	0,0008
105	BCP105	O73 - H74	0,3552	-2,2934	0,0227	0,6434	0,0008
106	BCP106	C30 - O76	0,2580	-0,5061	0,0082	0,3553	0,0009
107	BCP107	O76 - H77	0,3573	-2,3364	0,0228	0,6542	0,0008
108	BCP108	C21 - C81	0,2707	-0,6607	0,0952	0,2300	0,0001
109	BCP109	C81 - C82	0,2768	-0,6930	0,0938	0,2397	0,0002
110	BCP110	C81 - O83	0,4043	0,3507	0,0694	0,7059	0,0000

111	BCP111	C82 - H84	0,2833	-1,1106	0,0077	0,3111	0,0001
112	BCP112	C18 - O87	0,2597	-0,5753	0,0215	0,3400	0,0007
113	BCP113	O87 - C90	0,0059	0,0226	0,3716	-0,0012	0,4653
114	BCP114	O70 - H88	0,0227	0,0719	0,1006	0,0005	0,0521
115	BCP115	O87 - H88	0,3482	-2,3967	0,0221	0,6656	0,0007
116	BCP116	O70 - H115	0,0079	0,0335	1,2387	-0,0013	0,0937
117	BCP117	O70 - H114	0,0058	0,0260	3,2743	-0,0014	0,5980
118	BCP118	C44 - H115	0,0077	0,0230	1,5837	-0,0008	0,7311
119	BCP119	C89 - C90	0,3088	-0,7841	0,2114	0,2954	0,0000
120	BCP120	C90 - C91	0,3112	-0,7922	0,2192	0,2997	0,0001
121	BCP121	C89 - C92	0,3086	-0,7824	0,2113	0,2948	0,0000
122	BCP122	C91 - C94	0,3077	-0,7776	0,2411	0,2938	0,0011
123	BCP123	C92 - C93	0,3088	-0,7799	0,2229	0,2953	0,0000
124	BCP124	C93 - C94	0,3084	-0,7828	0,2483	0,2944	0,0010
125	BCP125	C95 - C98	0,3208	-0,8335	0,2396	0,3177	0,0001
126	BCP126	O87 - C95	0,0047	0,0163	0,4438	-0,0008	0,0367
127	BCP127	C8 - H109	0,0072	0,0251	0,8871	-0,0014	0,1328
128	BCP128	O49 - C97	0,0109	0,0474	1,6452	-0,0021	0,5417
129	BCP129	H51 - C100	0,0094	0,0289	0,4392	-0,0008	0,0322
130	BCP130	C95 - C96	0,3038	-0,7751	0,1671	0,2858	0,0000
131	BCP131	C96 - C97	0,3173	-0,8146	0,2444	0,3115	0,0001
132	BCP132	C98 - C99	0,2964	-0,7323	0,1694	0,2723	0,0000
133	BCP133	C94 - H112	0,0125	0,0493	0,9424	-0,0020	0,1133
134	BCP134	C99 - C102	0,2871	-0,7077	0,1583	0,2561	0,0008
135	BCP135	C99 - C100	0,2888	-0,6920	0,1769	0,2585	0,0003
136	BCP136	C97 - C100	0,2942	-0,7226	0,1733	0,2689	0,0001
137	BCP137	C101 - C118	0,2784	-0,6706	0,1162	0,2423	0,0004
138	BCP138	C100 - C101	0,2784	-0,6571	0,1513	0,2421	0,0006
139	BCP139	C102 - N105	0,3113	-0,9723	0,0863	0,4732	0,0014
140	BCP140	C94 - N105	0,2818	-0,8109	0,0573	0,3985	0,0003
141	BCP141	C102 - C103	0,3148	-0,8135	0,2477	0,3064	0,0006
142	BCP142	C103 - C104	0,3103	-0,7936	0,2094	0,2981	0,0003
143	BCP143	C101 - C104	0,3067	-0,7666	0,2187	0,2907	0,0007
144	BCP144	N105 - H106	0,3368	-1,5896	0,0459	0,4462	0,0003
145	BCP145	C103 - H107	0,2790	-1,0351	0,0221	0,2995	0,0000
146	BCP146	C104 - H108	0,2838	-1,0890	0,0075	0,3090	0,0000
147	BCP147	C96 - H109	0,2821	-1,0682	0,0094	0,3052	0,0000
148	BCP148	H110 - C129	0,0125	0,0437	0,6481	-0,0018	0,1242
149	BCP149	C97 - H110	0,2846	-1,0900	0,0131	0,3110	0,0001
150	BCP150	C95 - H111	0,2824	-1,0800	0,0090	0,3065	0,0000
151	BCP151	C98 - H112	0,2849	-1,0993	0,0103	0,3119	0,0001
152	BCP152	C91 - H113	0,2820	-1,0763	0,0145	0,3066	0,0001
153	BCP153	C89 - H114	0,2812	-1,0632	0,0143	0,3040	0,0000
154	BCP154	C90 - H115	0,2820	-1,0605	0,0120	0,3050	0,0000
155	BCP155	C93 - H116	0,2791	-1,0389	0,0173	0,2998	0,0000
156	BCP156	C92 - H117	0,2808	-1,0584	0,0110	0,3029	0,0000
157	BCP157	O49 - C118	0,0098	0,0366	0,1538	-0,0013	0,0089
158	BCP158	C118 - C129	0,2895	-0,7109	0,1469	0,2603	0,0001
159	BCP159	C118 - C119	0,2862	-0,6966	0,1438	0,2547	0,0000
160	BCP160	O64 - H126	0,0070	0,0297	1,3151	-0,0015	0,7317
161	BCP161	C119 - C120	0,2960	-0,7333	0,1672	0,2717	0,0004
162	BCP162	C120 - C121	0,3183	-0,8154	0,2575	0,3134	0,0003
163	BCP163	H69 - N148	0,0192	0,0556	0,0393	-0,0010	0,0051

164	BCP164	C38 - C123	0,0074	0,0220	0,6023	-0,0010	0,0610
165	BCP165	O60 - H128	0,0061	0,0221	1,2443	-0,0009	0,1780
166	BCP166	H128 - H156	0,0143	0,0665	1,1993	-0,0034	1,0634
167	BCP167	O50 - H127	0,0086	0,0254	0,0659	-0,0002	0,0061
168	BCP168	C119 - C122	0,2965	-0,7357	0,1661	0,2724	0,0003
169	BCP169	C122 - C123	0,3178	-0,8140	0,2512	0,3123	0,0003
170	BCP170	O64 - H150	0,0099	0,0342	0,0435	-0,0005	0,0085
171	BCP171	C121 - C124	0,2965	-0,7445	0,1784	0,2742	0,0007
172	BCP172	C123 - C124	0,2972	-0,7454	0,1849	0,2755	0,0007
173	BCP173	C120 - H125	0,2833	-1,0843	0,0078	0,3082	0,0000
174	BCP174	C121 - H126	0,2843	-1,0856	0,0180	0,3103	0,0001
175	BCP175	C122 - H127	0,2834	-1,1019	0,0083	0,3103	0,0001
176	BCP176	H75 - H156	0,0049	0,0193	0,2432	-0,0012	0,2382
177	BCP177	C123 - H128	0,2834	-1,0760	0,0213	0,3087	0,0001
178	BCP178	C129 - C130	0,2970	-0,7376	0,1668	0,2733	0,0003
179	BCP179	C132 - C133	0,3193	-0,8185	0,2640	0,3152	0,0002
180	BCP180	C1 - H137	0,0059	0,0216	0,5365	-0,0013	0,2185
181	BCP181	O47 - H147	0,0054	0,0191	0,2140	-0,0007	0,0083
182	BCP182	C5 - H138	0,0062	0,0192	1,0392	-0,0010	1,1772
183	BCP183	O53 - H137	0,0074	0,0245	0,0644	-0,0006	0,0189
184	BCP184	C130 - H138	0,2844	-1,1041	0,0058	0,3114	0,0001
185	BCP185	C120 - H138	0,0117	0,0553	3,4382	-0,0035	0,7481
186	BCP186	C134 - N141	0,3221	-0,8652	0,1049	0,5230	0,0000
187	BCP187	C130 - C131	0,3183	-0,8168	0,2522	0,3134	0,0003
188	BCP188	C129 - C132	0,2967	-0,7367	0,1682	0,2727	0,0003
189	BCP189	C131 - C134	0,2957	-0,7400	0,1823	0,2734	0,0006
190	BCP190	C133 - C134	0,2941	-0,7341	0,1784	0,2702	0,0007
191	BCP191	C132 - H135	0,2830	-1,0869	0,0061	0,3079	0,0000
192	BCP192	C133 - H136	0,2820	-1,0591	0,0231	0,3056	0,0001
193	BCP193	C131 - H137	0,2833	-1,0726	0,0229	0,3084	0,0002
194	BCP194	O53 - H138	0,0036	0,0157	2,7686	-0,0009	0,1233
195	BCP195	C139 - H144	0,2765	-1,0246	0,0363	0,2952	0,0005
196	BCP196	C140 - H145	0,2765	-1,0245	0,0347	0,2952	0,0005
197	BCP197	C139 - N141	0,2647	-0,7273	0,0394	0,3457	0,0000
198	BCP198	C140 - N141	0,2674	-0,7479	0,0419	0,3478	0,0000
199	BCP199	C139 - H142	0,2774	-1,0310	0,0358	0,2968	0,0005
200	BCP200	C139 - H143	0,2811	-1,0632	0,0315	0,3047	0,0007
201	BCP201	C140 - H146	0,2813	-1,0655	0,0300	0,3050	0,0006
202	BCP202	C140 - H147	0,2760	-1,0095	0,0362	0,2934	0,0005
203	BCP203	N148 - C149	0,2608	-0,7046	0,0355	0,3326	0,0002
204	BCP204	C124 - N148	0,3167	-0,9208	0,0886	0,4997	0,0007
205	BCP205	C149 - H152	0,2750	-1,0119	0,0368	0,2923	0,0006
206	BCP206	H126 - C149	0,0130	0,0621	1,6419	-0,0034	0,8511
207	BCP207	C149 - H150	0,2816	-1,0766	0,0330	0,3064	0,0005
208	BCP208	C149 - H151	0,2805	-1,0584	0,0331	0,3036	0,0007
209	BCP209	O60 - H156	0,0050	0,0176	0,0962	-0,0007	0,0197
210	BCP210	C153 - H154	0,2750	-1,0117	0,0365	0,2923	0,0006
211	BCP211	N148 - C153	0,2617	-0,7098	0,0359	0,3352	0,0002
212	BCP212	C153 - H155	0,2805	-1,0594	0,0327	0,3036	0,0007
213	BCP213	C153 - H156	0,2799	-1,0631	0,0329	0,3030	0,0006

GO2 + BG1

1	BCP1	C10 - O49	0,2337	-0,2242	0,8102	0,2606	0,0048
2	BCP2	C1 - C2	0,3144	-0,8089	0,2778	0,3065	0,0010
3	BCP3	C3 - C4	0,3053	-0,7601	0,2040	0,2871	0,0002
4	BCP4	C2 - H71	0,2777	-1,0281	0,0195	0,2974	0,0000
5	BCP5	C2 - C3	0,3013	-0,7481	0,1981	0,2805	0,0001
6	BCP6	C1 - C5	0,3042	-0,7735	0,2241	0,2860	0,0010
7	BCP7	C1 - O47	0,2886	-0,2242	0,0103	0,4500	0,0010
8	BCP8	C4 - C6	0,3105	-0,7805	0,2095	0,2965	0,0002
9	BCP9	C4 - C10	0,2719	-0,6645	0,0520	0,2316	0,0009
10	BCP10	C5 - C23	0,2569	-0,6096	0,0435	0,2102	0,0003
11	BCP11	C5 - C6	0,3106	-0,7716	0,2284	0,2971	0,0001
12	BCP12	C3 - C7	0,2844	-0,7048	0,0968	0,2515	0,0000
13	BCP13	C7 - C8	0,3237	-0,8359	0,2414	0,3228	0,0002
14	BCP14	C9 - C10	0,2733	-0,6744	0,0320	0,2328	0,0004
15	BCP15	C8 - C9	0,2897	-0,7138	0,1215	0,2606	0,0002
16	BCP16	C8 - C11	0,2679	-0,6486	0,1010	0,2260	0,0001
17	BCP17	C11 - C12	0,2683	-0,6470	0,1013	0,2268	0,0001
18	BCP18	C9 - C13	0,3250	-0,8302	0,2835	0,3253	0,0001
19	BCP19	C12 - C14	0,3214	-0,8064	0,2767	0,3197	0,0001
20	BCP20	C13 - C14	0,2823	-0,6784	0,1214	0,2482	0,0005
21	BCP21	C12 - C15	0,2706	-0,6615	0,0924	0,2299	0,0001
22	BCP22	C15 - C16	0,2710	-0,6641	0,0909	0,2306	0,0004
23	BCP23	C14 - C18	0,2552	-0,6122	0,0135	0,2073	0,0008
24	BCP24	C16 - C19	0,2744	-0,6698	0,0711	0,2345	0,0007
25	BCP25	C16 - C17	0,3287	-0,8401	0,2915	0,3333	0,0001
26	BCP26	C19 - C20	0,2407	-0,4495	0,3319	0,2004	0,0251
27	BCP27	C17 - C22	0,2790	-0,6677	0,1176	0,2430	0,0008
28	BCP28	C17 - C18	0,2521	-0,5902	0,0115	0,2030	0,0006
29	BCP29	C19 - H85	0,2866	-1,1306	0,0331	0,3148	0,0002
30	BCP30	C20 - C21	0,2756	-0,6743	0,0787	0,2363	0,0006
31	BCP31	C20 - H57	0,2867	-1,1351	0,0332	0,3155	0,0003
32	BCP32	C18 - C32	0,2549	-0,5979	0,0070	0,2071	0,0005
33	BCP33	C21 - C81	0,2709	-0,6615	0,0958	0,2303	0,0001
34	BCP34	C21 - C22	0,3261	-0,8306	0,2861	0,3280	0,0001
35	BCP35	C28 - O49	0,2512	-0,3223	0,5440	0,3247	0,0041
36	BCP36	C23 - C24	0,2640	-0,6444	0,0588	0,2197	0,0007
37	BCP37	C6 - C26	0,2593	-0,6277	0,0131	0,2128	0,0008
38	BCP38	C24 - C25	0,3318	-0,8472	0,3314	0,3398	0,0001
39	BCP39	C28 - C29	0,2429	-0,5554	0,0544	0,1907	0,0002
40	BCP40	C26 - C27	0,2512	-0,5834	0,0162	0,2024	0,0016
41	BCP41	C25 - C26	0,2610	-0,6259	0,0096	0,2150	0,0002
42	BCP42	C10 - C28	0,2564	-0,5359	0,1849	0,2199	0,0306
43	BCP43	C27 - C28	0,2711	-0,6558	0,0538	0,2297	0,0004
44	BCP44	C13 - C30	0,2536	-0,5924	0,0421	0,2054	0,0006
45	BCP45	C27 - C38	0,3289	-0,8408	0,2976	0,3338	0,0001
46	BCP46	C29 - C30	0,2397	-0,5480	0,0737	0,1874	0,0001
47	BCP47	C30 - C31	0,2613	-0,6280	0,0386	0,2157	0,0002
48	BCP48	C31 - C42	0,2709	-0,6310	0,0983	0,2321	0,0034
49	BCP49	C31 - C32	0,3301	-0,8329	0,3347	0,3376	0,0002
50	BCP50	C22 - C34	0,2717	-0,6653	0,0686	0,2309	0,0005
51	BCP51	C81 - C82	0,2767	-0,6921	0,0944	0,2396	0,0002
52	BCP52	C33 - C34	0,2433	-0,4714	0,2429	0,2030	0,0267
53	BCP53	C32 - C33	0,2710	-0,6553	0,0790	0,2301	0,0004

54	BCP54	C34 - C35	0,2704	-0,6594	0,0693	0,2287	0,0005
55	BCP55	C35 - C46	0,2829	-0,7091	0,1368	0,2483	0,0007
56	BCP56	C35 - C82	0,3305	-0,8549	0,2992	0,3386	0,0001
57	BCP57	C25 - C36	0,2756	-0,6577	0,0995	0,2381	0,0005
58	BCP58	C36 - C61	0,2554	-0,6037	0,0721	0,2080	0,0008
59	BCP59	C36 - C37	0,3309	-0,8524	0,3158	0,3389	0,0002
60	BCP60	C29 - O50	0,2801	-0,5443	0,0317	0,4183	0,0010
61	BCP61	C37 - H75	0,2834	-1,1093	0,0174	0,3118	0,0001
62	BCP62	C37 - C38	0,2788	-0,6766	0,0961	0,2440	0,0001
63	BCP63	C38 - C39	0,2724	-0,6738	0,0870	0,2325	0,0003
64	BCP64	C29 - C40	0,2476	-0,5665	0,0487	0,1984	0,0019
65	BCP65	C39 - C40	0,2742	-0,6776	0,1105	0,2353	0,0009
66	BCP66	C40 - C41	0,3325	-0,8507	0,2162	0,3478	0,0044
67	BCP67	C41 - C42	0,3212	-0,8261	0,1735	0,3221	0,0049
68	BCP68	C44 - C45	0,2886	-0,6996	0,1606	0,2594	0,0002
69	BCP69	C33 - C44	0,2718	-0,6587	0,0756	0,2317	0,0002
70	BCP70	C41 - H59	0,0224	0,0736	1,2716	-0,0005	0,2118
71	BCP71	C42 - C43	0,2929	-0,7383	0,1710	0,2650	0,0010
72	BCP72	C43 - C44	0,3237	-0,8437	0,3189	0,3222	0,0013
73	BCP73	C46 - O79	0,3010	-0,2137	0,0082	0,4789	0,0010
74	BCP74	C45 - C46	0,3248	-0,8414	0,3474	0,3292	0,0005
75	BCP75	O47 - H48	0,3556	-2,4427	0,0179	0,6761	0,0007
76	BCP76	O49 - H51	0,0312	0,1107	0,2110	0,0000	0,0945
77	BCP77	O49 - C101	0,0104	0,0398	0,8265	-0,0016	0,0502
78	BCP78	O50 - H51	0,3477	-2,3864	0,0217	0,6652	0,0005
79	BCP79	C11 - O52	0,4070	0,3675	0,0733	0,7114	0,0000
80	BCP80	O52 - O86	0,0145	0,0504	0,1995	-0,0003	0,0069
81	BCP81	O47 - H55	0,0217	0,0756	0,0221	-0,0003	0,0436
82	BCP82	C23 - O53	0,2684	-0,5554	0,0312	0,3826	0,0008
83	BCP83	C23 - H54	0,2741	-0,9989	0,0364	0,2887	0,0002
84	BCP84	O53 - H55	0,3552	-2,4413	0,0222	0,6788	0,0006
85	BCP85	C19 - O56	0,2603	-0,3691	0,4828	0,3538	0,0050
86	BCP86	C20 - O56	0,2570	-0,3635	0,5157	0,3415	0,0047
87	BCP87	C18 - O87	0,2560	-0,5477	0,0175	0,3337	0,0006
88	BCP88	C43 - O58	0,3077	-0,2458	0,0101	0,4944	0,0010
89	BCP89	O58 - H59	0,3399	-2,3107	0,0168	0,6420	0,0006
90	BCP90	C39 - O60	0,4034	0,3689	0,0536	0,7029	0,0000
91	BCP91	C61 - O67	0,2677	-0,4933	0,0034	0,3887	0,0011
92	BCP92	C24 - C63	0,2684	-0,6507	0,0648	0,2255	0,0006
93	BCP93	C61 - C62	0,2642	-0,6458	0,0150	0,2197	0,0009
94	BCP94	C62 - C63	0,2567	-0,5305	0,2283	0,2220	0,0307
95	BCP95	C62 - O64	0,2583	-0,3276	0,5701	0,3534	0,0037
96	BCP96	C63 - O64	0,2494	-0,3099	0,7065	0,3184	0,0034
97	BCP97	C63 - H65	0,2871	-1,1264	0,0324	0,3149	0,0002
98	BCP98	C62 - H66	0,2827	-1,0847	0,0362	0,3059	0,0002
99	BCP99	C37 - H107	0,0057	0,0172	0,1873	-0,0009	0,9356
100	BCP100	C61 - H68	0,2794	-1,0512	0,0387	0,2998	0,0001
101	BCP101	O67 - H69	0,3582	-2,2757	0,0234	0,6412	0,0007
102	BCP102	C33 - O70	0,2429	-0,2933	0,6184	0,2962	0,0045
103	BCP103	C34 - O70	0,2481	-0,3177	0,5545	0,3153	0,0044
104	BCP104	C7 - H72	0,2838	-1,1200	0,0019	0,3121	0,0003
105	BCP105	C26 - O73	0,2524	-0,5203	0,0234	0,3277	0,0009
106	BCP106	O73 - H74	0,3552	-2,2874	0,0224	0,6419	0,0008

107	BCP107	C30 - O76	0,2609	-0,5118	0,0113	0,3643	0,0009
108	BCP108	O76 - H77	0,3572	-2,3389	0,0225	0,6547	0,0008
109	BCP109	C45 - H78	0,2785	-1,0436	0,0225	0,3000	0,0000
110	BCP110	O79 - H80	0,3559	-2,4231	0,0163	0,6716	0,0008
111	BCP111	C81 - O83	0,4045	0,3500	0,0698	0,7063	0,0000
112	BCP112	C82 - H84	0,2834	-1,1111	0,0081	0,3112	0,0001
113	BCP113	C15 - O86	0,4075	0,4016	0,0729	0,7117	0,0000
114	BCP114	O87 - C121	0,0062	0,0211	1,4386	-0,0007	0,0608
115	BCP115	O70 - H88	0,0254	0,0833	0,0917	0,0002	0,0426
116	BCP116	C22 - H150	0,0035	0,0113	0,9530	-0,0006	0,3523
117	BCP117	O70 - H150	0,0053	0,0240	1,0521	-0,0013	0,3117
118	BCP118	O87 - H88	0,3478	-2,4022	0,0222	0,6674	0,0007
119	BCP119	O64 - H92	0,0051	0,0208	0,3216	-0,0009	0,0465
120	BCP120	C38 - H106	0,0044	0,0152	10,4031	-0,0007	0,2050
121	BCP121	C36 - H106	0,0054	0,0186	7,5207	-0,0010	0,8112
122	BCP122	O64 - H107	0,0044	0,0202	0,4908	-0,0012	0,3223
123	BCP123	C38 - N105	0,0036	0,0110	0,5785	-0,0004	0,0122
124	BCP124	H69 - H107	0,0033	0,0141	0,6457	-0,0009	0,0625
125	BCP125	C89 - C90	0,2487	-0,5776	0,0288	0,2014	0,0008
126	BCP126	C89 - H91	0,2734	-0,9828	0,0074	0,2898	0,0003
127	BCP127	C89 - H92	0,2750	-1,0143	0,0040	0,2944	0,0002
128	BCP128	C89 - H93	0,2735	-0,9839	0,0065	0,2901	0,0003
129	BCP129	C95 - N105	0,2587	-0,6839	0,0215	0,3338	0,0002
130	BCP130	H93 - H97	0,0052	0,0202	0,3271	-0,0011	0,1109
131	BCP131	C99 - N105	0,3269	-0,8738	0,1097	0,5361	0,0000
132	BCP132	O60 - H109	0,0042	0,0179	1,4995	-0,0010	0,2798
133	BCP133	C37 - H109	0,0026	0,0090	2,0009	-0,0006	0,1206
134	BCP134	C94 - C95	0,2487	-0,5777	0,0329	0,2009	0,0005
135	BCP135	C94 - H96	0,2729	-0,9805	0,0048	0,2889	0,0003
136	BCP136	C90 - N105	0,2572	-0,6781	0,0265	0,3277	0,0001
137	BCP137	C94 - H97	0,2733	-0,9829	0,0053	0,2897	0,0003
138	BCP138	C94 - H98	0,2735	-0,9925	0,0040	0,2908	0,0002
139	BCP139	C95 - H108	0,2823	-1,0645	0,0308	0,3051	0,0007
140	BCP140	H106 - H11(	0,0141	0,0614	0,8198	-0,0029	0,5554
141	BCP141	C99 - C104	0,2906	-0,7232	0,1627	0,2641	0,0008
142	BCP142	C99 - C100	0,2945	-0,7391	0,1711	0,2711	0,0006
143	BCP143	C100 - C101	0,3216	-0,8294	0,2656	0,3200	0,0005
144	BCP144	C100 - H11(	0,2830	-1,0712	0,0218	0,3078	0,0002
145	BCP145	O50 - C102	0,0115	0,0461	0,4848	-0,0018	0,3189
146	BCP146	C102 - C10[illegible]	0,2927	-0,7256	0,1455	0,2655	0,0003
147	BCP147	C101 - C10[illegible]	0,2925	-0,7201	0,1570	0,2658	0,0002
148	BCP148	C103 - C10[illegible]	0,3210	-0,8228	0,2779	0,3188	0,0004
149	BCP149	C90 - H106	0,2794	-1,0408	0,0337	0,2995	0,0006
150	BCP150	C90 - H107	0,2815	-1,0494	0,0334	0,3029	0,0007
151	BCP151	O60 - H108	0,0041	0,0179	6,8747	-0,0011	0,6338
152	BCP152	H108 - H11[illegible]	0,0140	0,0644	0,6819	-0,0033	0,7026
153	BCP153	C95 - H109	0,2801	-1,0474	0,0320	0,3010	0,0008
154	BCP154	C103 - H13[illegible]	0,0132	0,0601	1,2710	-0,0035	0,6408
155	BCP155	C103 - H111	0,2833	-1,0850	0,0063	0,3083	0,0001
156	BCP156	C104 - H11[illegible]	0,2818	-1,0579	0,0235	0,3052	0,0001
157	BCP157	C101 - H11[illegible]	0,2858	-1,1135	0,0062	0,3141	0,0001
158	BCP158	C11 - H124	0,0027	0,0100	0,4746	-0,0007	0,0846
159	BCP159	O87 - H122	0,0158	0,0471	0,0674	-0,0001	0,0006

160	BCP160	C114 - C115	0,3092	-0,7884	0,1992	0,2955	0,0000
161	BCP161	H113 - C119	0,0135	0,0588	6,5678	-0,0030	0,3957
162	BCP162	C118 - H126	0,2810	-1,0621	0,0127	0,3036	0,0000
163	BCP163	C119 - C120	0,2697	-0,6492	0,0717	0,2288	0,0002
164	BCP164	C115 - C116	0,3104	-0,7918	0,2046	0,2980	0,0001
165	BCP165	C114 - C117	0,3082	-0,7838	0,1997	0,2938	0,0000
166	BCP166	C117 - C118	0,3107	-0,7909	0,2176	0,2987	0,0000
167	BCP167	C102 - C120	0,2924	-0,7108	0,1795	0,2655	0,0003
168	BCP168	C116 - C119	0,3011	-0,7450	0,1954	0,2811	0,0003
169	BCP169	C118 - C119	0,3028	-0,7527	0,2037	0,2839	0,0004
170	BCP170	C127 - C128	0,3204	-0,8239	0,2650	0,3174	0,0004
171	BCP171	C120 - C121	0,2886	-0,7010	0,1615	0,2590	0,0001
172	BCP172	C128 - C131	0,2928	-0,7289	0,1752	0,2681	0,0006
173	BCP173	C116 - H122	0,2847	-1,1223	0,0118	0,3146	0,0000
174	BCP174	C114 - H123	0,2816	-1,0701	0,0077	0,3046	0,0000
175	BCP175	C115 - H124	0,2809	-1,0688	0,0111	0,3037	0,0000
176	BCP176	C117 - H125	0,2810	-1,0615	0,0117	0,3034	0,0000
177	BCP177	C121 - C127	0,2947	-0,7301	0,1592	0,2691	0,0002
178	BCP178	H88 - C131	0,0071	0,0238	4,3476	-0,0009	0,0851
179	BCP179	C144 - H147	0,2809	-1,0489	0,0304	0,3022	0,0007
180	BCP180	H133 - H146	0,0157	0,0611	0,5187	-0,0022	0,3015
181	BCP181	C127 - H132	0,2843	-1,0986	0,0057	0,3107	0,0001
182	BCP182	O50 - H134	0,0108	0,0395	0,2088	-0,0010	0,0553
183	BCP183	C129 - H134	0,2863	-1,1249	0,0062	0,3159	0,0001
184	BCP184	C121 - C129	0,2928	-0,7217	0,1543	0,2663	0,0004
185	BCP185	O70 - H139	0,0097	0,0366	0,2440	-0,0010	0,0658
186	BCP186	C44 - H135	0,0029	0,0091	0,8670	-0,0005	0,1694
187	BCP187	C45 - H139	0,0062	0,0177	0,3298	-0,0007	0,0426
188	BCP188	C131 - N136	0,3255	-0,8818	0,1115	0,5312	0,0001
189	BCP189	C129 - C130	0,3188	-0,8149	0,2660	0,3146	0,0005
190	BCP190	O70 - N136	0,0088	0,0319	0,9279	-0,0007	0,0180
191	BCP191	N136 - C144	0,2594	-0,6943	0,0275	0,3307	0,0001
192	BCP192	C130 - C131	0,2917	-0,7247	0,1731	0,2663	0,0008
193	BCP193	C128 - H133	0,2826	-1,0635	0,0236	0,3068	0,0002
194	BCP194	N136 - C137	0,2607	-0,6997	0,0275	0,3351	0,0001
195	BCP195	C138 - H141	0,2732	-0,9841	0,0037	0,2896	0,0002
196	BCP196	C130 - H135	0,2814	-1,0536	0,0247	0,3045	0,0001
197	BCP197	C137 - C138	0,2489	-0,5789	0,0347	0,2013	0,0004
198	BCP198	H135 - H139	0,0158	0,0606	0,4645	-0,0021	0,2805
199	BCP199	C137 - H139	0,2829	-1,0681	0,0295	0,3064	0,0007
200	BCP200	C137 - H140	0,2798	-1,0378	0,0315	0,2998	0,0007
201	BCP201	C138 - H142	0,2728	-0,9826	0,0044	0,2892	0,0002
202	BCP202	C138 - H143	0,2736	-0,9883	0,0040	0,2903	0,0002
203	BCP203	C35 - H150	0,0036	0,0117	3,9530	-0,0006	0,0357
204	BCP204	O56 - H148	0,0023	0,0108	0,1118	-0,0008	0,1066
205	BCP205	C145 - H148	0,2726	-0,9839	0,0061	0,2886	0,0003
206	BCP206	C144 - C145	0,2473	-0,5724	0,0320	0,1994	0,0004
207	BCP207	C144 - H146	0,2811	-1,0478	0,0319	0,3022	0,0007
208	BCP208	C145 - H149	0,2739	-0,9990	0,0054	0,2918	0,0003
209	BCP209	C145 - H150	0,2724	-0,9746	0,0058	0,2878	0,0003

GO2 + BR1

1	BCP1	C1 - C2	0,3147	-0,8109	0,2774	0,3070	0,0011
2	BCP2	C3 - C4	0,3045	-0,7571	0,2006	0,2856	0,0002
3	BCP3	C2 - C3	0,3018	-0,7500	0,1998	0,2814	0,0000
4	BCP4	C1 - C5	0,3046	-0,7750	0,2244	0,2870	0,0010
5	BCP5	C1 - O47	0,2883	-0,2239	0,0070	0,4495	0,0010
6	BCP6	C4 - C6	0,3102	-0,7787	0,2107	0,2960	0,0001
7	BCP7	O47 - H55	0,0230	0,0800	0,0160	-0,0003	0,0404
8	BCP8	C4 - C10	0,2712	-0,6614	0,0515	0,2300	0,0008
9	BCP9	C5 - C23	0,2569	-0,6098	0,0441	0,2104	0,0003
10	BCP10	C5 - C6	0,3092	-0,7655	0,2255	0,2945	0,0001
11	BCP11	C3 - C7	0,2847	-0,7056	0,0986	0,2520	0,0002
12	BCP12	C7 - C8	0,3247	-0,8407	0,2447	0,3249	0,0002
13	BCP13	C8 - C9	0,2896	-0,7140	0,1189	0,2600	0,0003
14	BCP14	C9 - C10	0,2724	-0,6699	0,0370	0,2314	0,0004
15	BCP15	C7 - H72	0,2831	-1,1074	0,0011	0,3100	0,0003
16	BCP16	C8 - C11	0,2688	-0,6517	0,1028	0,2272	0,0003
17	BCP17	C11 - C12	0,2711	-0,6574	0,1099	0,2307	0,0005
18	BCP18	C9 - C13	0,3288	-0,8494	0,2848	0,3324	0,0002
19	BCP19	C12 - C14	0,3220	-0,8126	0,2714	0,3207	0,0003
20	BCP20	C13 - C14	0,2856	-0,6979	0,1082	0,2534	0,0003
21	BCP21	C12 - C15	0,2731	-0,6709	0,0989	0,2336	0,0006
22	BCP22	C15 - C16	0,2726	-0,6710	0,0931	0,2329	0,0003
23	BCP23	C14 - C18	0,2563	-0,6180	0,0200	0,2090	0,0015
24	BCP24	C16 - C17	0,3258	-0,8271	0,2826	0,3272	0,0003
25	BCP25	C17 - C18	0,2529	-0,5942	0,0016	0,2041	0,0008
26	BCP26	C14 - O119	0,0042	0,0135	0,3204	-0,0005	1,0514
27	BCP27	C16 - C19	0,2768	-0,6767	0,0933	0,2382	0,0006
28	BCP28	C19 - H85	0,2868	-1,1361	0,0332	0,3159	0,0003
29	BCP29	C19 - C20	0,2335	-0,4078	0,4069	0,1910	0,0222
30	BCP30	C17 - C22	0,2788	-0,6654	0,1194	0,2429	0,0010
31	BCP31	C20 - O56	0,2640	-0,3930	0,4333	0,3633	0,0048
32	BCP32	C20 - C21	0,2780	-0,6816	0,0911	0,2399	0,0007
33	BCP33	C21 - C22	0,3234	-0,8184	0,2803	0,3226	0,0000
34	BCP34	C6 - C26	0,2598	-0,6295	0,0106	0,2134	0,0006
35	BCP35	C23 - O53	0,2701	-0,5637	0,0292	0,3867	0,0008
36	BCP36	C23 - C24	0,2643	-0,6463	0,0567	0,2202	0,0007
37	BCP37	C25 - C26	0,2606	-0,6234	0,0084	0,2145	0,0002
38	BCP38	C24 - C63	0,2682	-0,6500	0,0658	0,2254	0,0006
39	BCP39	C24 - C25	0,3318	-0,8470	0,3342	0,3399	0,0001
40	BCP40	C26 - C27	0,2512	-0,5838	0,0150	0,2021	0,0017
41	BCP41	C63 - H65	0,2866	-1,1187	0,0328	0,3136	0,0002
42	BCP42	C10 - C28	0,2562	-0,5334	0,1895	0,2197	0,0307
43	BCP43	C10 - O49	0,2349	-0,2345	0,7964	0,2622	0,0044
44	BCP44	C27 - C28	0,2698	-0,6503	0,0548	0,2276	0,0004
45	BCP45	C13 - C30	0,2565	-0,6107	0,0273	0,2096	0,0011
46	BCP46	C28 - C29	0,2407	-0,5465	0,0562	0,1881	0,0002
47	BCP47	C30 - O76	0,2629	-0,5422	0,0549	0,3657	0,0005
48	BCP48	C29 - C30	0,2362	-0,5300	0,0735	0,1828	0,0002
49	BCP49	C29 - O50	0,2828	-0,5745	0,0442	0,4230	0,0012
50	BCP50	C18 - C32	0,2546	-0,5960	0,0046	0,2068	0,0004
51	BCP51	C30 - C31	0,2598	-0,6212	0,0211	0,2137	0,0004
52	BCP52	C31 - C32	0,3285	-0,8246	0,3318	0,3343	0,0003

53	BCP53	C22 - C34	0,2705	-0,6609	0,0638	0,2287	0,0002
54	BCP54	C32 - C33	0,2689	-0,6468	0,0741	0,2270	0,0005
55	BCP55	C34 - O70	0,2510	-0,3476	0,5124	0,3191	0,0042
56	BCP56	C33 - C34	0,2429	-0,4699	0,2502	0,2021	0,0268
57	BCP57	C32 - C97	0,0023	0,0062	2,3622	-0,0003	0,0249
58	BCP58	C34 - C35	0,2682	-0,6516	0,0655	0,2253	0,0005
59	BCP59	C25 - C36	0,2760	-0,6589	0,1023	0,2386	0,0005
60	BCP60	C27 - C38	0,3294	-0,8446	0,2958	0,3347	0,0000
61	BCP61	C36 - C37	0,3310	-0,8539	0,3133	0,3392	0,0002
62	BCP62	C62 - O64	0,2629	-0,3508	0,5185	0,3656	0,0036
63	BCP63	C37 - C38	0,2790	-0,6775	0,0965	0,2443	0,0001
64	BCP64	C38 - C39	0,2724	-0,6714	0,0913	0,2326	0,0010
65	BCP65	C29 - C40	0,2458	-0,5596	0,0491	0,1962	0,0023
66	BCP66	C39 - C40	0,2729	-0,6710	0,1106	0,2334	0,0009
67	BCP67	C41 - C42	0,3219	-0,8283	0,1775	0,3234	0,0050
68	BCP68	C31 - C42	0,2712	-0,6321	0,1008	0,2327	0,0031
69	BCP69	C40 - C41	0,3327	-0,8516	0,2222	0,3483	0,0043
70	BCP70	C33 - C44	0,2720	-0,6599	0,0725	0,2318	0,0002
71	BCP71	C42 - C43	0,2938	-0,7449	0,1673	0,2663	0,0009
72	BCP72	C33 - O70	0,2418	-0,2947	0,6043	0,2887	0,0044
73	BCP73	C43 - C44	0,3240	-0,8455	0,3195	0,3231	0,0012
74	BCP74	C45 - H78	0,2791	-1,0496	0,0187	0,3010	0,0000
75	BCP75	C44 - C45	0,2903	-0,7076	0,1572	0,2619	0,0003
76	BCP76	C35 - C82	0,3300	-0,8544	0,2955	0,3375	0,0001
77	BCP77	C35 - C46	0,2852	-0,7174	0,1419	0,2518	0,0008
78	BCP78	C45 - C46	0,3265	-0,8514	0,3426	0,3321	0,0006
79	BCP79	O47 - H48	0,3558	-2,4381	0,0179	0,6752	0,0007
80	BCP80	C28 - O49	0,2533	-0,3389	0,5150	0,3294	0,0039
81	BCP81	O49 - H51	0,0295	0,1038	0,2375	0,0001	0,1094
82	BCP82	O50 - H51	0,3475	-2,3947	0,0221	0,6665	0,0006
83	BCP83	C11 - O52	0,4028	0,3155	0,0637	0,7036	0,0000
84	BCP84	O53 - H55	0,3558	-2,4450	0,0223	0,6802	0,0006
85	BCP85	C23 - H54	0,2732	-0,9883	0,0369	0,2867	0,0002
86	BCP86	C19 - O56	0,2618	-0,3837	0,4417	0,3578	0,0048
87	BCP87	C20 - H57	0,2856	-1,1169	0,0343	0,3124	0,0003
88	BCP88	C43 - O58	0,3080	-0,2368	0,0124	0,4951	0,0011
89	BCP89	C41 - O58	0,0215	0,0780	3,8137	-0,0011	0,5595
90	BCP90	O58 - H59	0,3422	-2,3374	0,0168	0,6488	0,0006
91	BCP91	C39 - O60	0,4029	0,3627	0,0531	0,7020	0,0000
92	BCP92	C62 - C63	0,2553	-0,5194	0,2511	0,2201	0,0305
93	BCP93	C61 - C62	0,2646	-0,6475	0,0147	0,2201	0,0009
94	BCP94	C36 - C61	0,2550	-0,6021	0,0701	0,2075	0,0009
95	BCP95	C61 - O67	0,2698	-0,5078	0,0097	0,3929	0,0011
96	BCP96	C62 - H66	0,2823	-1,0776	0,0358	0,3046	0,0002
97	BCP97	C63 - O64	0,2534	-0,3358	0,6570	0,3273	0,0030
98	BCP98	O67 - H69	0,3564	-2,3102	0,0229	0,6478	0,0007
99	BCP99	C61 - H68	0,2785	-1,0374	0,0389	0,2975	0,0001
100	BCP100	O70 - H88	0,0254	0,0817	0,0717	0,0002	0,0371
101	BCP101	C2 - H71	0,2771	-1,0204	0,0208	0,2962	0,0000
102	BCP102	C26 - O73	0,2426	-0,4724	0,0256	0,3022	0,0007
103	BCP103	O73 - H74	0,3543	-2,2598	0,0229	0,6353	0,0006
104	BCP104	C37 - H75	0,2836	-1,1088	0,0179	0,3119	0,0001
105	BCP105	C41 - H152	0,0046	0,0163	0,6449	-0,0009	0,0788

106	BCP106	C42 - O143	0,0080	0,0291	3,0956	-0,0013	0,6118
107	BCP107	O76 - H77	0,3406	-2,4008	0,0207	0,6686	0,0008
108	BCP108	C46 - O79	0,2981	-0,2202	0,0132	0,4722	0,0009
109	BCP109	O79 - H80	0,3564	-2,4240	0,0167	0,6721	0,0008
110	BCP110	C21 - C81	0,2722	-0,6653	0,1009	0,2322	0,0001
111	BCP111	C81 - C82	0,2823	-0,7146	0,1051	0,2482	0,0002
112	BCP112	C81 - O83	0,3988	0,2769	0,0542	0,6959	0,0000
113	BCP113	C82 - H84	0,2824	-1,0945	0,0129	0,3086	0,0001
114	BCP114	O52 - O86	0,0148	0,0515	0,1419	-0,0003	0,0043
115	BCP115	C15 - O86	0,4057	0,3689	0,0649	0,7086	0,0000
116	BCP116	C18 - O87	0,2596	-0,5759	0,0181	0,3376	0,0008
117	BCP117	O87 - H88	0,3489	-2,4050	0,0223	0,6685	0,0007
118	BCP118	C81 - N106	0,0102	0,0372	0,3130	-0,0016	0,0589
119	BCP119	O83 - H91	0,0098	0,0345	0,0992	-0,0008	0,0331
120	BCP120	C82 - H109	0,0047	0,0183	1,0722	-0,0011	0,1119
121	BCP121	C90 - N106	0,2552	-0,6489	0,0306	0,3396	0,0004
122	BCP122	C89 - C90	0,2505	-0,5873	0,0243	0,2045	0,0007
123	BCP123	C89 - H91	0,2754	-1,0174	0,0103	0,2949	0,0003
124	BCP124	C89 - H92	0,2742	-1,0001	0,0088	0,2923	0,0002
125	BCP125	C89 - H93	0,2730	-0,9805	0,0100	0,2890	0,0004
126	BCP126	C82 - H101	0,0047	0,0141	0,7181	-0,0006	0,2998
127	BCP127	C94 - C99	0,2826	-0,6892	0,1513	0,2509	0,0010
128	BCP128	C94 - C95	0,3194	-0,8074	0,2917	0,3147	0,0003
129	BCP129	C110 - O14[illegible]	0,0134	0,0472	0,6703	-0,0013	0,0272
130	BCP130	C95 - C96	0,2932	-0,7237	0,1584	0,2668	0,0002
131	BCP131	C22 - C98	0,0068	0,0187	0,8179	-0,0008	0,1436
132	BCP132	C96 - C97	0,2996	-0,7627	0,1911	0,2794	0,0016
133	BCP133	C98 - C99	0,2994	-0,7465	0,2191	0,2787	0,0002
134	BCP134	C99 - N106	0,3275	-0,8831	0,0996	0,5397	0,0005
135	BCP135	C97 - C98	0,3204	-0,8408	0,3186	0,3218	0,0009
136	BCP136	C90 - H108	0,2818	-1,0527	0,0335	0,3035	0,0003
137	BCP137	C100 - H10[illegible]	0,2733	-0,9848	0,0120	0,2901	0,0003
138	BCP138	C94 - C100	0,2515	-0,5812	0,0320	0,2047	0,0005
139	BCP139	C100 - H101	0,2744	-0,9993	0,0111	0,2925	0,0003
140	BCP140	C100 - H10[illegible]	0,2752	-1,0015	0,0087	0,2935	0,0004
141	BCP141	H103 - H10[illegible]	0,0108	0,0432	0,6017	-0,0020	0,3222
142	BCP142	C95 - H104	0,2823	-1,0767	0,0127	0,3065	0,0000
143	BCP143	C98 - H105	0,2781	-1,0353	0,0289	0,2989	0,0001
144	BCP144	N106 - H10[illegible]	0,3387	-1,6116	0,0441	0,4505	0,0000
145	BCP145	C90 - H109	0,2792	-1,0396	0,0353	0,2987	0,0003
146	BCP146	H101 - H10[illegible]	0,0081	0,0379	4,0174	-0,0025	1,0677
147	BCP147	C96 - C110	0,3008	-0,7442	0,1881	0,2797	0,0001
148	BCP148	C110 - C111	0,2990	-0,7351	0,1901	0,2769	0,0002
149	BCP149	C97 - O119	0,2872	-0,0280	0,0151	0,4457	0,0026
150	BCP150	C111 - C11[illegible]	0,2973	-0,7499	0,1945	0,2752	0,0017
151	BCP151	O76 - C113	0,0063	0,0218	3,6730	-0,0008	0,0525
152	BCP152	C111 - C11[illegible]	0,2916	-0,7179	0,1561	0,2641	0,0001
153	BCP153	O73 - H131	0,0100	0,0296	0,0723	-0,0002	0,0072
154	BCP154	C113 - C11[illegible]	0,3230	-0,8239	0,2976	0,3217	0,0002
155	BCP155	O52 - H127	0,0133	0,0370	0,1206	0,0002	0,0115
156	BCP156	C114 - C11[illegible]	0,2859	-0,7061	0,1421	0,2553	0,0006
157	BCP157	C12 - H118	0,0063	0,0217	0,8904	-0,0012	0,2732
158	BCP158	C112 - O11[illegible]	0,2840	-0,0199	0,0161	0,4385	0,0027

159	BCP159	C112 - C116	0,3176	-0,8322	0,2988	0,3172	0,0007
160	BCP160	C115 - C116	0,3022	-0,7589	0,2180	0,2841	0,0002
161	BCP161	H117 - C138	0,0107	0,0393	1,1497	-0,0019	0,1004
162	BCP162	C113 - H117	0,2825	-1,0676	0,0160	0,3063	0,0001
163	BCP163	C116 - H118	0,2803	-1,0631	0,0279	0,3041	0,0000
164	BCP164	C16 - O119	0,0034	0,0116	4,9332	-0,0005	0,6803
165	BCP165	C2 - H124	0,0049	0,0156	0,6259	-0,0008	0,0250
166	BCP166	C115 - N120	0,3294	-0,8804	0,0905	0,5450	0,0004
167	BCP167	C7 - H127	0,0070	0,0260	2,9136	-0,0013	0,5444
168	BCP168	N120 - C121	0,2587	-0,6728	0,0514	0,3449	0,0002
169	BCP169	C122 - H123	0,2726	-0,9761	0,0096	0,2881	0,0003
170	BCP170	C121 - C122	0,2515	-0,5908	0,0252	0,2049	0,0007
171	BCP171	C122 - H124	0,2727	-0,9743	0,0095	0,2883	0,0003
172	BCP172	C122 - H125	0,2749	-1,0101	0,0050	0,2940	0,0001
173	BCP173	C121 - H126	0,2772	-1,0202	0,0363	0,2948	0,0005
174	BCP174	C121 - H127	0,2827	-1,0791	0,0330	0,3072	0,0004
175	BCP175	H128 - C129	0,0136	0,0674	4,8715	-0,0032	0,3988
176	BCP176	N120 - H128	0,3395	-1,6182	0,0448	0,4530	0,0001
177	BCP177	C114 - C129	0,2565	-0,5991	0,0348	0,2113	0,0002
178	BCP178	C129 - H130	0,2744	-0,9965	0,0078	0,2923	0,0003
179	BCP179	C129 - H132	0,2697	-0,9495	0,0118	0,2828	0,0003
180	BCP180	C129 - H131	0,2728	-1,0055	0,0081	0,2914	0,0002
181	BCP181	C133 - C138	0,2995	-0,7284	0,2125	0,2776	0,0005
182	BCP182	C110 - C133	0,2640	-0,6339	0,0424	0,2198	0,0008
183	BCP183	C134 - H142	0,2820	-1,0735	0,0140	0,3057	0,0000
184	BCP184	C134 - C135	0,3101	-0,7922	0,2042	0,2974	0,0000
185	BCP185	C133 - C134	0,3043	-0,7548	0,2188	0,2875	0,0001
186	BCP186	C135 - H139	0,2821	-1,0781	0,0077	0,3059	0,0000
187	BCP187	C135 - C136	0,3105	-0,7950	0,1986	0,2978	0,0000
188	BCP188	C137 - C138	0,3068	-0,7684	0,2042	0,2910	0,0001
189	BCP189	C136 - H140	0,2822	-1,0792	0,0095	0,3062	0,0000
190	BCP190	C136 - C137	0,3113	-0,7978	0,2019	0,2995	0,0000
191	BCP191	C138 - C144	0,2741	-0,6961	0,1087	0,2370	0,0003
192	BCP192	C137 - H141	0,2834	-1,0955	0,0093	0,3094	0,0000
193	BCP193	H77 - O143	0,0368	0,1446	0,0567	-0,0036	0,0044
194	BCP194	O143 - C144	0,4078	0,2348	0,0851	0,7192	0,0003
195	BCP195	C144 - O145	0,3202	-0,2564	0,0103	0,5245	0,0023
196	BCP196	O145 - C147	0,2289	-0,1924	0,0292	0,3128	0,0015
197	BCP197	C37 - H150	0,0065	0,0219	0,4084	-0,0011	0,0892
198	BCP198	O60 - H150	0,0042	0,0172	0,9766	-0,0009	0,1281
199	BCP199	O60 - H152	0,0087	0,0306	0,2365	-0,0008	0,0705
200	BCP200	C147 - H151	0,2815	-1,0676	0,0446	0,3035	0,0006
201	BCP201	C146 - C147	0,2589	-0,6280	0,0324	0,2159	0,0008
202	BCP202	C146 - H149	0,2743	-1,0008	0,0087	0,2923	0,0002
203	BCP203	H130 - H148	0,0004	0,0015	1,0670	-0,0001	0,1315
204	BCP204	C146 - H148	0,2724	-0,9737	0,0077	0,2877	0,0003
205	BCP205	C146 - H150	0,2745	-1,0074	0,0057	0,2937	0,0001
206	BCP206	C147 - H152	0,2854	-1,1194	0,0400	0,3134	0,0005

GO2 + BY2

1	BCP1	C10 - O49	0,2326	-0,2203	0,8116	0,2573	0,0050

2	BCP2	C1 - C2	0,3139	-0,8056	0,2796	0,3057	0,0011
3	BCP3	C3 - C4	0,3064	-0,7645	0,2060	0,2890	0,0002
4	BCP4	C2 - H71	0,2777	-1,0283	0,0200	0,2974	0,0000
5	BCP5	C2 - C3	0,3004	-0,7435	0,1981	0,2789	0,0001
6	BCP6	C1 - C5	0,3042	-0,7736	0,2246	0,2861	0,0010
7	BCP7	C1 - O47	0,2884	-0,2228	0,0113	0,4496	0,0010
8	BCP8	C4 - C6	0,3116	-0,7868	0,2065	0,2984	0,0001
9	BCP9	C4 - C10	0,2717	-0,6633	0,0522	0,2311	0,0009
10	BCP10	C5 - C23	0,2588	-0,6180	0,0392	0,2126	0,0003
11	BCP11	C5 - C6	0,3118	-0,7774	0,2302	0,2994	0,0002
12	BCP12	C3 - C7	0,2839	-0,7022	0,0962	0,2506	0,0002
13	BCP13	C7 - C8	0,3236	-0,8340	0,2444	0,3227	0,0003
14	BCP14	C9 - C10	0,2725	-0,6698	0,0375	0,2316	0,0004
15	BCP15	C8 - C9	0,2882	-0,7060	0,1217	0,2580	0,0002
16	BCP16	C8 - C11	0,2664	-0,6426	0,0995	0,2240	0,0003
17	BCP17	C11 - C12	0,2674	-0,6444	0,0991	0,2254	0,0003
18	BCP18	C9 - C13	0,3242	-0,8250	0,2855	0,3238	0,0002
19	BCP19	C13 - C14	0,2810	-0,6746	0,1148	0,2461	0,0004
20	BCP20	C12 - C15	0,2689	-0,6543	0,0919	0,2275	0,0002
21	BCP21	C12 - C14	0,3220	-0,8097	0,2757	0,3206	0,0001
22	BCP22	O52 - O86	0,0154	0,0533	0,1103	-0,0003	0,0035
23	BCP23	C15 - O86	0,4087	0,4096	0,0746	0,7143	0,0000
24	BCP24	C15 - C16	0,2707	-0,6622	0,0916	0,2302	0,0005
25	BCP25	C14 - C18	0,2547	-0,6099	0,0139	0,2067	0,0011
26	BCP26	C16 - C19	0,2744	-0,6702	0,0737	0,2345	0,0008
27	BCP27	C16 - C17	0,3285	-0,8398	0,2916	0,3330	0,0001
28	BCP28	C17 - C22	0,2776	-0,6608	0,1207	0,2408	0,0009
29	BCP29	C17 - C18	0,2519	-0,5892	0,0092	0,2029	0,0004
30	BCP30	C19 - C20	0,2399	-0,4437	0,3472	0,1994	0,0245
31	BCP31	C20 - C21	0,2762	-0,6767	0,0805	0,2372	0,0006
32	BCP32	C20 - H57	0,2867	-1,1352	0,0332	0,3155	0,0003
33	BCP33	C20 - O56	0,2588	-0,3713	0,5025	0,3464	0,0047
34	BCP34	C21 - C22	0,3257	-0,8282	0,2856	0,3271	0,0001
35	BCP35	C6 - C26	0,2613	-0,6361	0,0141	0,2157	0,0009
36	BCP36	C10 - C28	0,2555	-0,5330	0,1819	0,2188	0,0304
37	BCP37	C23 - C24	0,2641	-0,6445	0,0536	0,2198	0,0007
38	BCP38	C27 - C28	0,2700	-0,6510	0,0543	0,2280	0,0005
39	BCP39	C24 - C25	0,3321	-0,8481	0,3357	0,3406	0,0001
40	BCP40	C26 - C27	0,2526	-0,5888	0,0184	0,2042	0,0017
41	BCP41	C25 - C26	0,2619	-0,6308	0,0110	0,2163	0,0002
42	BCP42	C28 - C29	0,2449	-0,5637	0,0555	0,1933	0,0002
43	BCP43	C13 - C30	0,2516	-0,5839	0,0364	0,2028	0,0009
44	BCP44	C27 - C38	0,3295	-0,8425	0,3004	0,3349	0,0001
45	BCP45	C29 - C30	0,2397	-0,5477	0,0715	0,1874	0,0001
46	BCP46	C18 - C32	0,2553	-0,6001	0,0125	0,2075	0,0004
47	BCP47	C30 - C31	0,2583	-0,6158	0,0373	0,2116	0,0003
48	BCP48	C31 - C32	0,3304	-0,8336	0,3377	0,3380	0,0002
49	BCP49	C22 - C34	0,2711	-0,6624	0,0704	0,2300	0,0004
50	BCP50	C33 - C34	0,2421	-0,4664	0,2396	0,2015	0,0262
51	BCP51	C34 - C35	0,2702	-0,6587	0,0722	0,2285	0,0005
52	BCP52	C32 - C33	0,2699	-0,6508	0,0801	0,2283	0,0004
53	BCP53	C81 - C82	0,2775	-0,6960	0,0939	0,2408	0,0002
54	BCP54	C82 - H84	0,2834	-1,1118	0,0079	0,3113	0,0001

55	BCP55	C35 - C46	0,2825	-0,7083	0,1341	0,2476	0,0008
56	BCP56	C35 - C82	0,3313	-0,8585	0,3017	0,3402	0,0001
57	BCP57	C25 - C36	0,2763	-0,6620	0,0955	0,2391	0,0004
58	BCP58	C36 - C61	0,2550	-0,6020	0,0718	0,2075	0,0010
59	BCP59	C36 - C37	0,3305	-0,8507	0,3141	0,3382	0,0002
60	BCP60	C62 - C63	0,2563	-0,5294	0,2274	0,2213	0,0313
61	BCP61	C38 - C39	0,2711	-0,6685	0,0859	0,2305	0,0003
62	BCP62	C61 - O67	0,2689	-0,5010	0,0067	0,3912	0,0011
63	BCP63	C37 - H75	0,2837	-1,1157	0,0156	0,3128	0,0001
64	BCP64	C37 - C38	0,2777	-0,6727	0,0921	0,2420	0,0001
65	BCP65	C39 - C40	0,2740	-0,6763	0,1128	0,2349	0,0009
66	BCP66	C29 - O50	0,2784	-0,5316	0,0313	0,4148	0,0010
67	BCP67	C29 - C40	0,2491	-0,5721	0,0565	0,2004	0,0021
68	BCP68	C31 - C42	0,2699	-0,6282	0,0941	0,2305	0,0029
69	BCP69	C40 - C41	0,3335	-0,8558	0,2194	0,3500	0,0045
70	BCP70	C41 - C42	0,3235	-0,8366	0,1801	0,3262	0,0054
71	BCP71	C44 - C45	0,2879	-0,6969	0,1591	0,2582	0,0002
72	BCP72	C45 - C46	0,3253	-0,8433	0,3507	0,3304	0,0006
73	BCP73	C33 - C44	0,2720	-0,6588	0,0810	0,2319	0,0002
74	BCP74	C41 - H59	0,0218	0,0753	1,8678	-0,0008	0,3060
75	BCP75	C42 - C43	0,2931	-0,7393	0,1698	0,2653	0,0010
76	BCP76	C46 - O79	0,3018	-0,2114	0,0064	0,4808	0,0010
77	BCP77	C43 - C44	0,3246	-0,8470	0,3234	0,3237	0,0012
78	BCP78	O47 - H48	0,3554	-2,4430	0,0178	0,6761	0,0007
79	BCP79	C28 - O49	0,2491	-0,3146	0,5507	0,3189	0,0042
80	BCP80	C38 - C101	0,0043	0,0097	0,6824	-0,0003	0,7420
81	BCP81	O49 - O50	0,0285	0,1031	0,3201	0,0001	0,6117
82	BCP82	O50 - H51	0,3523	-2,3560	0,0218	0,6594	0,0004
83	BCP83	C11 - O52	0,4082	0,3833	0,0754	0,7138	0,0000
84	BCP84	O47 - H55	0,0196	0,0674	0,0451	-0,0002	0,0492
85	BCP85	C23 - O53	0,2636	-0,5316	0,0320	0,3712	0,0007
86	BCP86	C23 - H54	0,2756	-1,0122	0,0350	0,2918	0,0002
87	BCP87	O53 - H55	0,3543	-2,4186	0,0222	0,6728	0,0005
88	BCP88	C19 - O56	0,2619	-0,3765	0,4712	0,3580	0,0049
89	BCP89	C43 - O58	0,3067	-0,2455	0,0108	0,4920	0,0011
90	BCP90	O58 - H59	0,3412	-2,3197	0,0170	0,6444	0,0006
91	BCP91	C39 - O60	0,4051	0,3851	0,0577	0,7062	0,0000
92	BCP92	C24 - C63	0,2680	-0,6490	0,0655	0,2249	0,0007
93	BCP93	C61 - C62	0,2643	-0,6465	0,0116	0,2198	0,0010
94	BCP94	C62 - O64	0,2576	-0,3120	0,5736	0,3542	0,0036
95	BCP95	C63 - O64	0,2497	-0,3075	0,7073	0,3209	0,0033
96	BCP96	C63 - H65	0,2860	-1,1119	0,0335	0,3121	0,0002
97	BCP97	C62 - H66	0,2831	-1,0875	0,0356	0,3065	0,0002
98	BCP98	C37 - H129	0,0067	0,0172	0,7236	-0,0006	0,7656
99	BCP99	C61 - H68	0,2795	-1,0512	0,0378	0,2999	0,0001
100	BCP100	O67 - H69	0,3580	-2,2864	0,0233	0,6436	0,0007
101	BCP101	C33 - O70	0,2424	-0,2959	0,6141	0,2945	0,0045
102	BCP102	C34 - O70	0,2466	-0,3131	0,5603	0,3111	0,0045
103	BCP103	C7 - H72	0,2836	-1,1200	0,0010	0,3120	0,0003
104	BCP104	C26 - O73	0,2505	-0,5123	0,0234	0,3209	0,0009
105	BCP105	O73 - H74	0,3550	-2,2895	0,0225	0,6424	0,0008
106	BCP106	C30 - O76	0,2624	-0,5146	0,0082	0,3687	0,0010
107	BCP107	O76 - H77	0,3571	-2,3378	0,0224	0,6544	0,0008

108	BCP108	O79 - H80	0,3562	-2,4201	0,0163	0,6710	0,0008
109	BCP109	C45 - H78	0,2783	-1,0406	0,0238	0,2996	0,0000
110	BCP110	C21 - C81	0,2706	-0,6606	0,0935	0,2298	0,0001
111	BCP111	C81 - O83	0,4047	0,3547	0,0699	0,7066	0,0000
112	BCP112	C19 - H85	0,2869	-1,1344	0,0326	0,3156	0,0002
113	BCP113	C18 - O87	0,2525	-0,5370	0,0192	0,3224	0,0006
114	BCP114	O87 - H112	0,0109	0,0316	0,0650	-0,0001	0,0161
115	BCP115	O70 - H88	0,0270	0,0879	0,0724	0,0002	0,0363
116	BCP116	O87 - H88	0,3495	-2,4083	0,0223	0,6699	0,0006
117	BCP117	C89 - C94	0,3187	-0,8175	0,2571	0,3143	0,0003
118	BCP118	O70 - H113	0,0053	0,0201	0,1105	-0,0007	0,0050
119	BCP119	C89 - C90	0,2956	-0,7397	0,1807	0,2724	0,0006
120	BCP120	C90 - C91	0,2947	-0,7340	0,1842	0,2707	0,0006
121	BCP121	C91 - C92	0,3164	-0,8054	0,2610	0,3098	0,0002
122	BCP122	C92 - C93	0,2995	-0,7434	0,1885	0,2775	0,0004
123	BCP123	C93 - C95	0,2858	-0,7110	0,1548	0,2588	0,0002
124	BCP124	C93 - C94	0,2972	-0,7303	0,1835	0,2739	0,0004
125	BCP125	O50 - C95	0,0122	0,0501	1,4210	-0,0015	0,4476
126	BCP126	C95 - N119	0,3307	-0,7550	0,0452	0,5579	0,0000
127	BCP127	O49 - C96	0,0085	0,0292	0,8487	-0,0009	0,0253
128	BCP128	C97 - C98	0,3196	-0,8202	0,2669	0,3160	0,0002
129	BCP129	C95 - C96	0,2875	-0,7136	0,1689	0,2617	0,0002
130	BCP130	C96 - C97	0,2975	-0,7338	0,1815	0,2740	0,0004
131	BCP131	C25 - N121	0,0026	0,0081	0,7301	-0,0003	0,2004
132	BCP132	O64 - H124	0,0104	0,0417	0,2117	-0,0013	0,0795
133	BCP133	C98 - C99	0,2941	-0,7352	0,1734	0,2695	0,0005
134	BCP134	O50 - C100	0,0080	0,0324	2,4370	-0,0015	0,1785
135	BCP135	C100 - H115	0,2841	-1,1028	0,0066	0,3109	0,0001
136	BCP136	C96 - C100	0,2964	-0,7319	0,1788	0,2725	0,0003
137	BCP137	H69 - H129	0,0038	0,0163	0,8395	-0,0010	0,0800
138	BCP138	C100 - C101	0,3180	-0,8129	0,2631	0,3130	0,0003
139	BCP139	C99 - C101	0,2939	-0,7332	0,1769	0,2694	0,0006
140	BCP140	C45 - H105	0,0049	0,0145	0,5159	-0,0006	0,0201
141	BCP141	O70 - H105	0,0032	0,0143	0,8496	-0,0008	0,0188
142	BCP142	C90 - N104	0,3237	-0,8863	0,1118	0,5253	0,0000
143	BCP143	C102 - N104	0,2648	-0,7289	0,0413	0,3450	0,0000
144	BCP144	C103 - N104	0,2660	-0,7366	0,0399	0,3470	0,0000
145	BCP145	C102 - H105	0,2774	-1,0342	0,0353	0,2972	0,0005
146	BCP146	C89 - H113	0,2823	-1,0637	0,0233	0,3064	0,0001
147	BCP147	C102 - H106	0,2767	-1,0247	0,0364	0,2955	0,0005
148	BCP148	C102 - H107	0,2808	-1,0580	0,0320	0,3039	0,0007
149	BCP149	C103 - H109	0,2765	-1,0228	0,0360	0,2950	0,0005
150	BCP150	C103 - H108	0,2811	-1,0628	0,0310	0,3046	0,0007
151	BCP151	C103 - H110	0,2766	-1,0233	0,0358	0,2952	0,0005
152	BCP152	C91 - H111	0,2819	-1,0573	0,0245	0,3053	0,0001
153	BCP153	C94 - H112	0,2831	-1,0868	0,0081	0,3084	0,0002
154	BCP154	C92 - H114	0,2823	-1,0777	0,0076	0,3063	0,0000
155	BCP155	C92 - H115	0,0101	0,0420	1,9038	-0,0025	0,5401
156	BCP156	C97 - H116	0,2801	-1,0446	0,0095	0,3011	0,0001
157	BCP157	C98 - H117	0,2828	-1,0676	0,0228	0,3072	0,0001
158	BCP158	C101 - H118	0,2817	-1,0580	0,0241	0,3051	0,0001
159	BCP159	N119 - H120	0,3350	-1,6415	0,0382	0,4539	0,0000
160	BCP160	O64 - H128	0,0086	0,0352	0,2699	-0,0013	0,1550

161	BCP161	C99 - N121	0,3277	-0,8948	0,1183	0,5363	0,0001
162	BCP162	O53 - H123	0,0088	0,0266	0,0463	-0,0003	0,0048
163	BCP163	N121 - C122	0,2613	-0,6962	0,0279	0,3447	0,0000
164	BCP164	C122 - H125	0,2757	-1,0094	0,0391	0,2930	0,0007
165	BCP165	C122 - H124	0,2837	-1,0952	0,0302	0,3109	0,0007
166	BCP166	C122 - H123	0,2787	-1,0547	0,0360	0,3008	0,0006
167	BCP167	C126 - H127	0,2766	-1,0215	0,0356	0,2951	0,0006
168	BCP168	N121 - C126	0,2652	-0,7295	0,0325	0,3471	0,0000
169	BCP169	C126 - H128	0,2829	-1,0865	0,0280	0,3090	0,0005
170	BCP170	C126 - H129	0,2765	-1,0186	0,0361	0,2948	0,0004
171	BCP171	N119 - H130	0,3349	-1,6419	0,0387	0,4541	0,0000

	rho	Delsqrt rho		
C9 - C124	0,0076	0,0239	0,4135	0,482
C7 - O37	0,4109	0,4467	0,002	
C16 - O38	0,3816	0,0647		
H36 - O38	0,0436	0,1419		
O40 - H44	0,0548	0,1712		
C32 - O40	0,3988	0,3137		
O40 - O79	0,0177	0,0741		
C2 - N133	0,0065	0,0184		
C29 - H117	0,0070	0,0223		
O66 - O67	0,0113	0,0410		
C25 - C95	0,0075	0,0214		
C61 - O66	0,3886	0,2220		
O66 - H73	0,0249	0,0885		
H78 - H80	0,0208	0,0673		
C76 - O77	0,4082	0,4210		
O66 - H108	0,0100	0,0296		
C62 - N97	0,0078	0,0258		
H68 - H108	0,0076	0,0261		
C51 - C90	0,0027	0,0063		
C86 - H104	0,0129	0,0504		
O70 - H98	0,0169	0,0479		
C48 - H99	0,0073	0,0240		
C89 - C124	0,0131	0,0578		
H55 - C112	0,0139	0,0402		
O43 - H144	0,0032	0,0130		
C112 - H130	0,0137	0,0646		
C16 - H137	0,0051	0,0180		
C3 - C125	0,0079	0,0243		
C2 - H136	0,0071	0,0291		
C7 - H128	0,0082	0,0313		
O41 - H127	0,0196	0,0625		
O37 - H136	0,0070	0,0228		
O34 - H135	0,0091	0,0335		
O34 - H138	0,0084	0,0366		
H128 - H136	0,0155	0,0576		
C15 - C119	0,0068	0,0239		
C16 - O38	0,3829	0,0760		
C21 - H103	0,0079	0,0282		
O40 - H44	0,0603	0,1815		
O40 - O79	0,0193	0,0838		
H36 - O38	0,0303	0,0942		
C7 - O37	0,4120	0,4639		
C32 - O40	0,3959	0,2790		
H78 - H80	0,0217	0,0686		
O66 - H69	0,0145	0,0473		
C25 - C120	0,0066	0,0181		
C61 - O66	0,3921	0,2684		
C48 - H138	0,0100	0,0298		
O66 - H73	0,0247	0,0879		
C76 - O77	0,4080	0,4192		
O38 - H85	0,0083	0,0307		
O34 - H83	0,0049	0,0201		

C5 - C92	0,0020	0,0052
H83 - H102	0,0089	0,0409
C17 - H88	0,0054	0,0206
O58 - H88	0,0110	0,0337
H100 - H104	0,0153	0,0599
H98 - H102	0,0145	0,0644
H55 - H103	0,0055	0,0208
O41 - C110	0,0059	0,0200
C3 - H118	0,0108	0,0326
O37 - H117	0,0068	0,0264
H105 - C110	0,0138	0,0581
H103 - H126	0,0129	0,0587
H55 - C121	0,0070	0,0183
C108 - H124	0,0137	0,0596
O41 - H124	0,0104	0,0329
H127 - H131	0,0156	0,0617
C75 - H135	0,0044	0,0130
C75 - H139	0,0039	0,0116
O40 - H133	0,0038	0,0146
O79 - H135	0,0055	0,0221
O70 - H141	0,0053	0,0215
H125 - H138	0,0155	0,0606
O70 - H138	0,0062	0,0273
C10 - C108	0,0061	0,0168
C7 - O37	0,4135	0,4820
C15 - O111	0,0054	0,0181
C22 - C104	0,0036	0,0094
O40 - H44	0,0573	0,1750
C32 - O40	0,3972	0,3087
O40 - O79	0,0176	0,0746
H36 - O38	0,0376	0,1223
C5 - H119	0,0077	0,0253
C16 - O38	0,3830	0,0637
C14 - C90	0,0067	0,0178
O66 - H69	0,0184	0,0571
C61 - O66	0,3857	0,1958
O66 - H73	0,0243	0,0874
C76 - O77	0,4084	0,4315
H78 - H80	0,0219	0,0690
C52 - N98	0,0073	0,0255
O66 - H83	0,0057	0,0219
C61 - N98	0,0064	0,0197
O72 - H99	0,0061	0,0235
O72 - H83	0,0076	0,0284
O66 - H101	0,0074	0,0271
C62 - H93	0,0055	0,0156
O70 - H93	0,0099	0,0351
O41 - H97	0,0101	0,0393
H95 - H100	0,0106	0,0448
H93 - H101	0,0102	0,0446
H55 - C105	0,0052	0,0163
O41 - O111	0,0086	0,0328
C20 - C107	0,0046	0,0125

C29 - H123	0,0047	0,0166
C18 - H123	0,0052	0,0169
C6 - N112	0,0064	0,0189
O38 - H116	0,0073	0,0248
C2 - H119	0,0057	0,0183
O34 - C114	0,0059	0,0268
O34 - H119	0,0063	0,0243
C27 - H123	0,0050	0,0182
C102 - O135	0,0137	0,0470
H55 - O135	0,0227	0,0788
O43 - H143	0,0051	0,0229
O40 - H143	0,0067	0,0275
O79 - H144	0,0075	0,0235
O135 - C136	0,4085	0,2271
O40 - H144	0,0066	0,0272
H36 - O38	0,0365	0,1178
C16 - O38	0,3823	0,0629
C22 - H106	0,0065	0,0217
O40 - H44	0,0557	0,1744
C23 - C83	0,0082	0,0242
C32 - O40	0,3995	0,3212
O40 - O79	0,0189	0,0808
C7 - O37	0,4108	0,4873
H78 - H80	0,0218	0,0682
O66 - H69	0,0160	0,0514
C26 - C82	0,0053	0,0162
C61 - O66	0,3922	0,2700
O66 - H73	0,0246	0,0876
C76 - O77	0,4091	0,4365
C25 - N96	0,0055	0,0163
C46 - H98	0,0052	0,0178
H55 - H103	0,0031	0,0130
O41 - C85	0,0098	0,0355
O41 - H112	0,0173	0,0637
O37 - N111	0,0070	0,0234
C3 - C89	0,0076	0,0254
O37 - H108	0,0057	0,0210
O34 - H109	0,0104	0,0311
O34 - H117	0,0089	0,0269
C5 - C90	0,0065	0,0171
O38 - H117	0,0072	0,0300
C17 - N113	0,0057	0,0156
O70 - H99	0,0124	0,0354
C94 - H105	0,0142	0,0687
C48 - H101	0,0075	0,0280
O70 - H100	0,0054	0,0213
H101 - H103	0,0141	0,0563
H106 - H107	0,0139	0,0610
H108 - H122	0,0156	0,0606
H109 - H117	0,0157	0,0587
O58 - H120	0,0037	0,0163
C18 - H119	0,0046	0,0169
H110 - C118	0,0136	0,0707

C15 - O86	0,4083	0,4136
O49 - O50	0,0250	0,0943
O50 - H113	0,0083	0,0263
C11 - O52	0,4067	0,3643
O52 - O86	0,0154	0,0530
O47 - H55	0,0205	0,0736
C41 - H59	0,0225	0,0724
C39 - O60	0,4023	0,3560
C81 - O83	0,4043	0,3507
O87 - C90	0,0059	0,0226
O70 - H88	0,0227	0,0719
O70 - H115	0,0079	0,0335
O70 - H114	0,0058	0,0260
C44 - H115	0,0077	0,0230
O87 - C95	0,0047	0,0163
C8 - H109	0,0072	0,0251
O49 - C97	0,0109	0,0474
H51 - C100	0,0094	0,0289
C94 - H112	0,0125	0,0493
H110 - C129	0,0125	0,0437
O49 - C118	0,0098	0,0366
O64 - H126	0,0070	0,0297
H69 - N148	0,0192	0,0556
C38 - C123	0,0074	0,0220
O60 - H128	0,0061	0,0221
H128 - H156	0,0143	0,0665
O50 - H127	0,0086	0,0254
O64 - H150	0,0099	0,0342
H75 - H156	0,0049	0,0193
C1 - H137	0,0059	0,0216
O47 - H147	0,0054	0,0191
C5 - H138	0,0062	0,0192
O53 - H137	0,0074	0,0245
C120 - H138	0,0117	0,0553
O53 - H138	0,0036	0,0157
H126 - C149	0,0130	0,0621
O60 - H156	0,0050	0,0176
C41 - H59	0,0224	0,0736
O49 - H51	0,0312	0,1107
O49 - C101	0,0104	0,0398
C11 - O52	0,4070	0,3675
O52 - O86	0,0145	0,0504
O47 - H55	0,0217	0,0756
C39 - O60	0,4034	0,3689
C37 - H107	0,0057	0,0172
C81 - O83	0,4045	0,3500
C15 - O86	0,4075	0,4016
O87 - C121	0,0062	0,0211
O70 - H88	0,0254	0,0833
C22 - H150	0,0035	0,0113
O70 - H150	0,0053	0,0240
O64 - H92	0,0051	0,0208
C38 - H106	0,0044	0,0152

C36 - H106	0,0054	0,0186
O64 - H107	0,0044	0,0202
C38 - N105	0,0036	0,0110
H69 - H107	0,0033	0,0141
H93 - H97	0,0052	0,0202
O60 - H109	0,0042	0,0179
C37 - H109	0,0026	0,0090
H106 - H110	0,0141	0,0614
O50 - C102	0,0115	0,0461
O60 - H108	0,0041	0,0179
H108 - H112	0,0140	0,0644
C103 - H134	0,0132	0,0601
C11 - H124	0,0027	0,0100
O87 - H122	0,0158	0,0471
H113 - C119	0,0135	0,0588
H88 - C131	0,0071	0,0238
H133 - H146	0,0157	0,0611
O50 - H134	0,0108	0,0395
O70 - H139	0,0097	0,0366
C44 - H135	0,0029	0,0091
C45 - H139	0,0062	0,0177
O70 - N136	0,0088	0,0319
H135 - H139	0,0158	0,0606
C35 - H150	0,0036	0,0117
O56 - H148	0,0023	0,0108
O47 - H55	0,0230	0,0800
C14 - O119	0,0042	0,0135
C32 - C97	0,0023	0,0062
O49 - H51	0,0295	0,1038
C11 - O52	0,4028	0,3155
C41 - O58	0,0215	0,0780
C39 - O60	0,4029	0,3627
O70 - H88	0,0254	0,0817
C41 - H152	0,0046	0,0163
C42 - O143	0,0080	0,0291
C81 - O83	0,3988	0,2769
O52 - O86	0,0148	0,0515
C15 - O86	0,4057	0,3689
C81 - N106	0,0102	0,0372
O83 - H91	0,0098	0,0345
C82 - H109	0,0047	0,0183
C82 - H101	0,0047	0,0141
C110 - O143	0,0134	0,0472
C22 - C98	0,0068	0,0187
H103 - H108	0,0108	0,0432
H101 - H109	0,0081	0,0379
O76 - C113	0,0063	0,0218
O73 - H131	0,0100	0,0296
O52 - H127	0,0133	0,0370
C12 - H118	0,0063	0,0217
H117 - C138	0,0107	0,0393
C16 - O119	0,0034	0,0116
C2 - H124	0,0049	0,0156

C7 - H127	0,0070	0,0260
H128 - C129	0,0136	0,0674
H77 - O143	0,0368	0,1446
O143 - C144	0,4078	0,2348
C37 - H150	0,0065	0,0219
O60 - H150	0,0042	0,0172
O60 - H152	0,0087	0,0306
H130 - H148	0,0004	0,0015
O52 - O86	0,0154	0,0533
C15 - O86	0,4087	0,4096
C41 - H59	0,0218	0,0753
C38 - C101	0,0043	0,0097
O49 - O50	0,0285	0,1031
C11 - O52	0,4082	0,3833
O47 - H55	0,0196	0,0674
C39 - O60	0,4051	0,3851
C37 - H129	0,0067	0,0172
C81 - O83	0,4047	0,3547
O87 - H112	0,0109	0,0316
O70 - H88	0,0270	0,0879
O70 - H113	0,0053	0,0201
O50 - C95	0,0122	0,0501
O49 - C96	0,0085	0,0292
C25 - N121	0,0026	0,0081
O64 - H124	0,0104	0,0417
O50 - C100	0,0080	0,0324
H69 - H129	0,0038	0,0163
C45 - H105	0,0049	0,0145
O70 - H105	0,0032	0,0143
C92 - H115	0,0101	0,0420
O64 - H128	0,0086	0,0352
O53 - H123	0,0088	0,0266
